# Continuous evolution of Eurasian avian-like H1N1 swine influenza viruses with pdm/09-derived internal genes enhances pathogenicity in mice

**DOI:** 10.1128/jvi.00430-25

**Published:** 2025-09-08

**Authors:** Riguo Lan, Jizhe Yang, Jixiang Li, Han Li, Xihao Cao, Mengyan Tao, Haoyu Chang, Haili Yu, Qi Tong, Lu Lu, Jinhua Liu, Honglei Sun

**Affiliations:** 1 National Key Laboratory of Veterinary Public Health and Safety, Key Laboratory for Prevention and Control of Avian Influenza and Other Major Poultry Diseases, Ministry of Agriculture and Rural Affairs, College of Veterinary Medicine, China Agricultural University, Beijing, China; 2 The Roslin Institute, University of Edinburgh 15551, Edinburgh, Scotland, United Kingdom; Emory University School of Medicine, Atlanta, Georgia, USA

**Keywords:** swine, Eurasian avian-like H1N1, 2009 pandemic H1N1, evolution, pandemic potential

## Abstract

Swine influenza A virus (swIAV) is an important zoonotic pathogen with the potential to cause human influenza pandemics. Swine are considered “mixing vessels” for generating novel reassortant influenza A viruses. In 2009, a swine-origin reassortant virus (2009 pandemic H1N1, pdm/09 H1N1) spilled over to humans, causing a global influenza pandemic. This virus soon spread back into swine herds and reassorted with the circulating swIAVs. We previously reported that the genotype 4 (G4) reassortant Eurasian avian-like (EA) H1N1 virus, which bore pdm/09- and triple reassortant (TR)-derived internal genes, had been predominant in swine populations of China since 2016, posing a threat to both the swine industry and public health. Here, our ongoing surveillance confirmed that G4 EA H1N1 viruses remained the predominant swIAVs in China from 2019 to 2023 and had reassorted with the co-circulating swIAVs, such as the H3N2 virus, to generate novel reassortant EA H1N2 viruses. Genetic analyses revealed that the pdm/09-derived internal genes of G4 EA H1N1 viruses originated from reassortments between pdm/09 H1N1 and EA H1N1 viruses in 2009–2010 and underwent independent and continuous evolution in the swine host, exhibiting higher evolutionary rates than those of the pdm/09 H1N1 virus circulating in humans. The accelerated evolution of internal genes enhanced the polymerase activity of G4 EA H1N1 viruses in mammalian cells, resulting in increased viral replication and pathogenicity in mice. This study provides evidence for swine in promoting the genetic evolution of influenza A virus and highlights the need for increased attention to novel reassortant viruses in swine.

**IMPORTANCE:**

The emergence of pdm/09 H1N1 virus highlights the role of swine influenza A viruses (swIAVs) in generating novel influenza viruses with pandemic potential. Since 2009, the pdm/09 H1N1 virus has been frequently transmitted to swine and reassorted with the circulating swIAVs, generating many new reassortant viruses bearing pdm/09-derived genes globally. The G4 EA H1N1 viruses, which bore pdm/09-derived internal genes and acquired increased human infectivity, remained the predominant swIAVs in China from 2019 to 2023 and reassorted with the co-circulating swIAVs to generate novel subtype viruses. The internal genes of G4 EA H1N1 viruses originated from the human pdm/09 H1N1 viruses during 2009–2010 and exhibited higher evolutionary rates and greater genetic diversity than those in the human host. This has contributed to increased viral adaptation and pathogenicity in mammals. Therefore, sustained surveillance and immunization efforts are essential to control emerging reassortant swIAVs and protect public health.

## INTRODUCTION

Influenza A virus (IAV) is one of the most prevalent pathogens of both humans and animals. IAVs that have caused pandemics in history were primarily generated through genetic reassortment. Historically, the 1918 Spanish flu, 1957 Asian flu, 1968 Hong Kong flu, and 2009 Mexico flu were all reassortant viruses (1–3). Migratory birds are generally considered reservoir hosts of IAVs, harboring a large pool of virus gene segments that contribute to the emergence of novel reassortant viruses. Swine, on the other hand, are often regarded as “mixing vessels” or at least intermediate hosts that IAVs can utilize to “jump” from poultry to humans (4–6). The pdm/09 H1N1 virus was a reassorted virus with all gene segments originating from swine influenza A viruses (swIAVs). Each gene segment of the pdm/09 H1N1 virus has been evolving in swine for an extended period, with data suggesting that its precursor had been circulating in swine populations for 5–10 years prior to the 2009 pandemic (2). Therefore, continuous surveillance of swIAVs is essential for the preparedness of human pandemics.

Shortly after the pandemic, the pdm/09 H1N1 virus transmitted to swine herds and further reassorted with the circulating swIAVs (7, 8). Since then, the reassortants bearing the pdm/09-derived internal genes have become prevalent in swine populations around the world. In the United States, the matrix (M), nucleoprotein (NP), and polymerase acid (PA) genes of pdm/09 lineage were commonly found in the triple reassortant (TR) swIAVs—a lineage that originated in 1998 through genetic reassortment among human H3N2, classical swine (CS) lineage H1N1, and avian influenza viruses and became widespread in North America (9, 10). Reports show that the TR lineage swIAVs containing the pdm/09-derived M gene have infected more than 300 people since 2011 (11–13). The complete viral ribonucleoprotein (vRNP) genes of pdm/09 H1N1 virus have been introduced into the EA lineage swIAVs in Europe and China, increasing the risk of human infection with these EA lineage reassortant viruses (14–16). Since 2016, the G4 reassortant EA H1N1 virus has become the predominant swIAV in China (17–19). This virus possesses the hemagglutinin (HA) and neuraminidase (NA) genes from the EA H1N1 lineage, the vRNP and M genes from the pdm/09 lineage, and the nonstructural (NS) gene from the TR lineage. It shows efficient infectivity and aerosol transmission in ferrets and has low antigenic cross-reactivity with the human influenza vaccine strain, indicating that this novel reassortant virus poses a threat to human public health (18). Recent studies have revealed that the G4 EA H1N1 virus is still being detected in pig farms and has shown increased pathogenicity in swine (20, 21). Thus, understanding the prevalence, evolution, and biological properties of the G4 EA H1N1 virus is important for the swine industry and public health safety.

In this study, we conducted an extensive swIAV surveillance program in seven provinces of China from 2019 to 2023, revealing that G4 EA H1N1 viruses remain the predominant swIAVs currently circulating in swine populations. Genetic evolution of these viruses was analyzed, and viral characteristics were evaluated. We found that the continuous and faster evolution of pdm/09-derived internal genes in swine host enhances the pathogenicity of G4 EA H1N1 viruses in mice, suggesting an increased risk to public health.

## RESULTS

### G4 EA H1N1 viruses are the predominant swIAVs from 2019 to 2023

**Fig 1:**
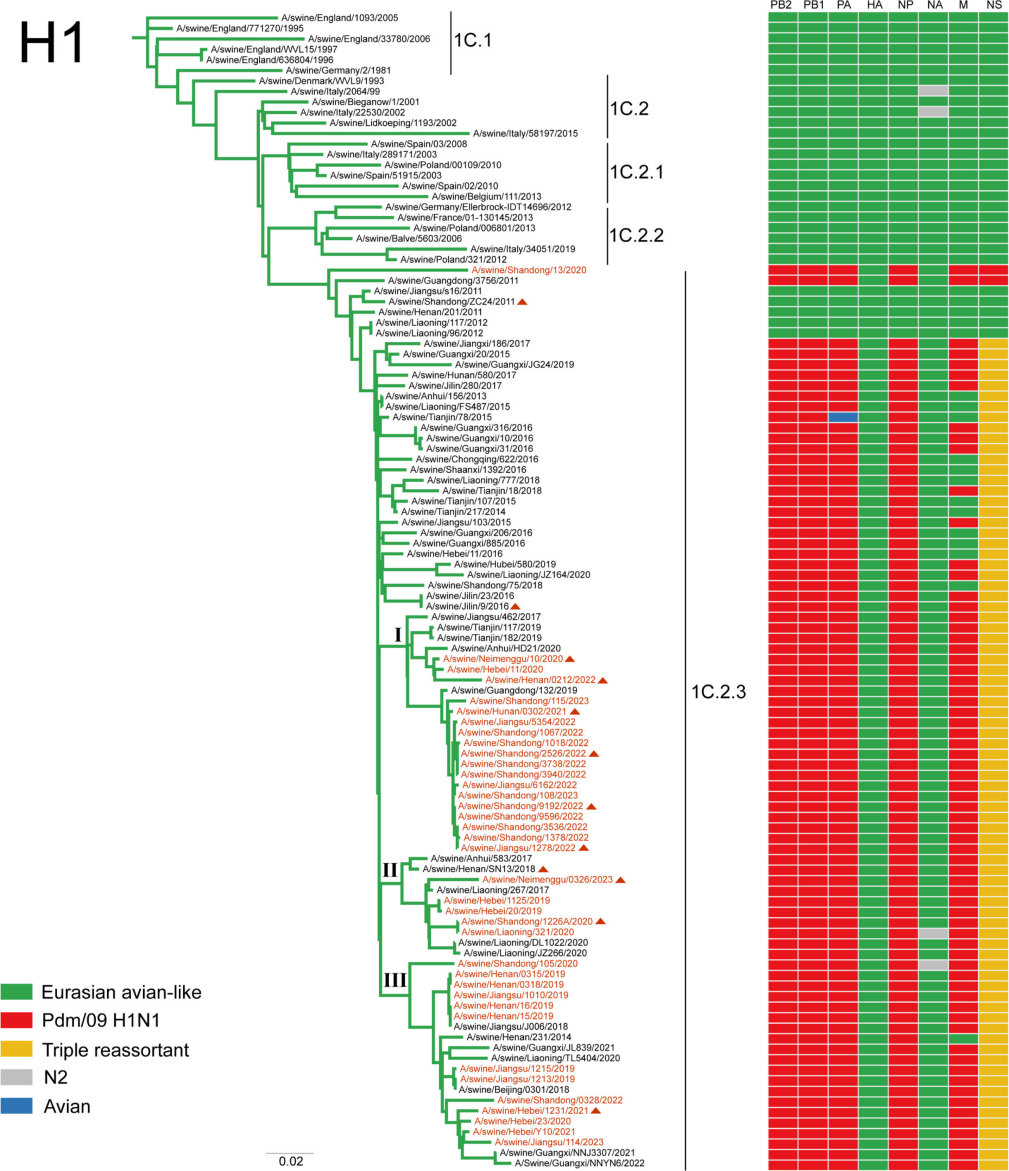
Phylogenetic analysis of the HA gene of EA H1N1 or EA H1N2 swIAVs isolated from 2019 to 2023 in China. Phylogenetic tree of the HA gene was estimated using genetic distances calculated by maximum likelihood under the GTRGAMMA + I model. The sequences of viruses with names listed in black were downloaded from the GISAID or NCBI database. Names of SIVs isolated in this study were marked with red, and three evolutionary branches were marked with I, II, and III. The strains selected for further evaluation of viral characteristics were marked with triangles. Node labels represent bootstrap values. Scale bar indicates estimated genetic distance. Colored boxes show the lineage classification of each gene segment of EA viruses. A detailed phylogenetic analysis of other genes of the isolated viruses is shown in Fig. S1.

To investigate the epidemiological status of swIAVs from 2019 to 2023, we continuously performed both active and passive surveillance, collecting a total of 7,679 nasal swabs and lung samples from pigs at slaughterhouses or farms in seven provinces with high-density swine population. We isolated 42 IAV strains from these samples, yielding an isolation rate of 0.54% (Table S1). Based on sequence analysis of the HA and NA genes of the 42 swIAVs, they were identified as EA H1N1 (*n* = 35), EA H1N2 (*n* = 2), pdm/09 H1N2 (*n* = 1), and H3N2 (*n* = 4) viruses (Table S2). Phylogenetic analysis showed that the 35 EA H1N1 viruses were clustered within clade 1C.2.3. In addition, 34 of them shared the same internal gene constellation, with the polymerase basic 2 (PB2), polymerase basic 1 (PB1), PA, NP, and M genes derived from the pdm/09 H1N1 lineage and the NS gene derived from the TR lineage, indicating that these viruses belonged to the G4 EA H1N1 viruses (Fig. 1; Fig. S1). Although the G4 EA H1N1 viruses isolated during 2020–2023 evolved into three evolutionary branches (I, II, and III) in the HA phylogenetic tree (Fig. 1), the viruses from different branches exhibited strong cross-reactivity with ferret antisera raised against the G4 EA H1N1 viruses isolated in 2018, with hemagglutination inhibition (HI) titers ≥ 1:640 and less than twofold differences from the reference strains (Table S3), indicating that no significant antigenic drift had occurred. Phylogenetic analysis showed that the two EA H1N2 viruses also bore the internal genes from pdm/09 and TR lineage, which were similar to those in G4 EA H1N1 viruses. The NA gene of A/swine/Shandong/105/2020 belonged to the recent human-like H3N2 lineage swIAV, which originated from seasonal human H3N2 viruses and is currently circulating in swine populations in China (22) (Fig. S1). In contrast, the NA gene of A/swine/Liaoning/321/2020 virus belonged to the TR lineage swIAV (Fig. S1). These findings suggest that G4 EA H1N1 viruses remain the predominant genotype circulating in swine populations and dynamically reassort with other swIAV lineages.

### The pdm/09-derived internal genes of the G4 EA H1N1 virus undergo independent and accelerated evolution in the swine host

**Fig 2:**
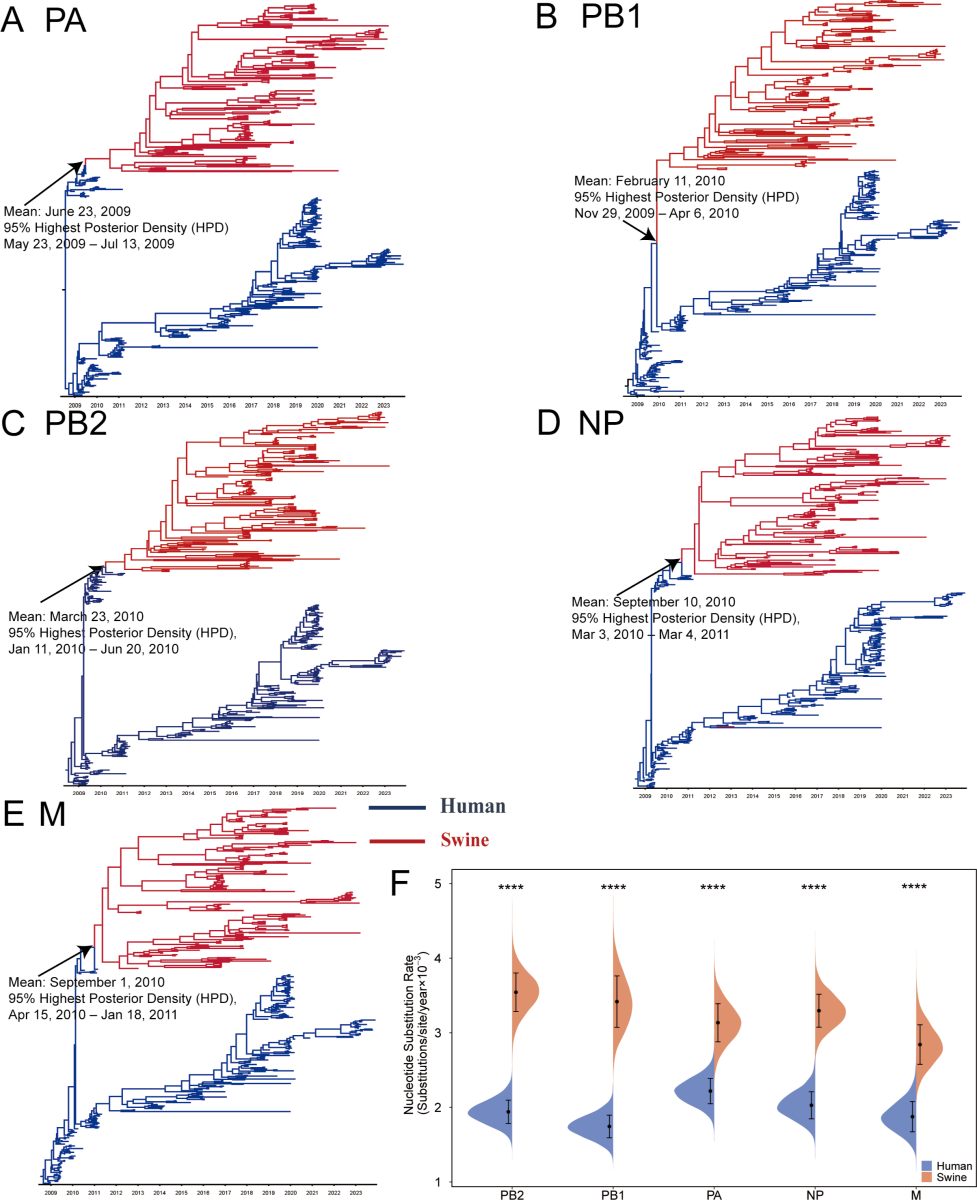
The pdm/09-derived internal genes of G4 EA H1N1 virus originated from reassortments between pdm/09 H1N1 and EA H1N1 viruses in 2009–2010 and underwent continuous evolution in the swine host. (**A through E**) The time-scaled Bayesian MCC trees were inferred for the internal gene sequences (PB2, PB1, PA, NP, and M) of G4 EA H1N1 and pdm/09 H1N1 viruses in China from 2009 to 2023. Branches of human seasonal pdm/09 H1N1 virus are shaded in blue, while branches associated with G4 EA H1N1 viruses from swine are shaded in red. (**F**) Mean evolutionary rate (substitutions/site/year) of human (seasonal pdm/09 H1N1) and swine (G4 EA H1N1) influenza viruses was estimated by BEAST analysis.

Since its emergence in 2013, the G4 EA H1N1 virus has circulated in swine populations for over 10 years (15, 17, 18). During this period, the seasonal pdm/09 H1N1 virus has remained prevalent in humans, with frequent human-to-swine spillover events reported each year (23). However, it remains unclear whether these spillover events have contributed pdm/09-derived internal genes to G4 EA H1N1 virus through dynamic reassortments in swine. To investigate the reassortment events of G4 EA H1N1 virus in swine host, we reconstructed phylogenetic trees of the pdm/09-derived internal genes of human pdm/09 H1N1 and all the G4 EA H1N1 viruses in China. From the phylogenetic trees, each of the internal genes of G4 EA H1N1 viruses was clustered in the clade that was different from human pdm/09 H1N1 viruses, implying that each of these genes was derived from a single reassortment event (Fig. 2A through E). By using a Bayesian molecular clock analysis, we estimated that the time of the most recent common ancestor (TMRCA) of the PB2, PB1, PA, NP, and M genes in swine was approximately between June 2009 and September 2010 (mean, 95% highest posterior density [HPD]) (Fig. 2A through E). The results indicate that shortly after the pdm/09 H1N1 virus re-entered swine populations, its internal genes were reassorted into the EA H1N1 virus and have since undergone evolution within the swine populations.

To evaluate the genetic variation of pdm/09-derived genes in swine and human hosts, the evolutionary rate of each gene was calculated. We observed higher evolution rates for the internal genes of G4 EA H1N1 viruses in swine populations compared to pdm/09 H1N1 viruses sampled from human populations. Specifically, the mean evolutionary rates of PB2, PB1, PA, NP, and M genes in swine host were 3.5436 × 10^−3^, 3.4176 × 10^−3^, 3.1352 × 10^−3^, 3.2958 × 10^−3^, and 2.8417 × 10^−3^ nucleotide substitutions per site per year (subs/site/year) (95% HPD), respectively. These rates were 1.82-, 1.96-, 1.41-, 1.62-, and 1.51-fold higher than those observed in the corresponding genes from human hosts (Fig. 2F; Fig. S4). We then compared the amino acid sequence divergence between the pdm/09 lineage genes in swine and human hosts with A/California/04/2009, one of the earliest human isolates of the pdm/09 H1N1 virus, and transformed the difference of each viral gene into a heat map (Fig. S2). Notably, the internal genes in G4 EA H1N1 viruses showed more heterogeneous patterns, indicating a higher degree of genetic diversity. The expansion of genetic diversity has resulted in the accumulation of more amino acid mutations within key critical functional domains of vRNP genes in G4 EA H1N1 viruses, including the C-terminal domain of PA, the palm and thumb domain of PB1, the 627 domain of PB2, and the RNA binding domain of NP. These amino acid substitutions differed from those observed in human influenza viruses, as evidenced by the absence of four amino acids (V100I, N321K, I330V, and A639T) in the PA protein of G4 EA H1N1 viruses, which were previously identified as markers of the human seasonal pdm/09 H1N1 viruses (24, 25). These findings suggest that swine play a crucial role in driving the genetic evolution of IAVs. The independent and accelerated evolution of pdm/09-derived internal genes in swine contributes to increased viral quasispecies diversity, which may facilitate the emergence of G4 EA H1N1 variants with enhanced pathogenicity to mammals.

### G4 EA H1N1 viruses enhance the polymerase activities in mammalian cells

**Fig 3:**
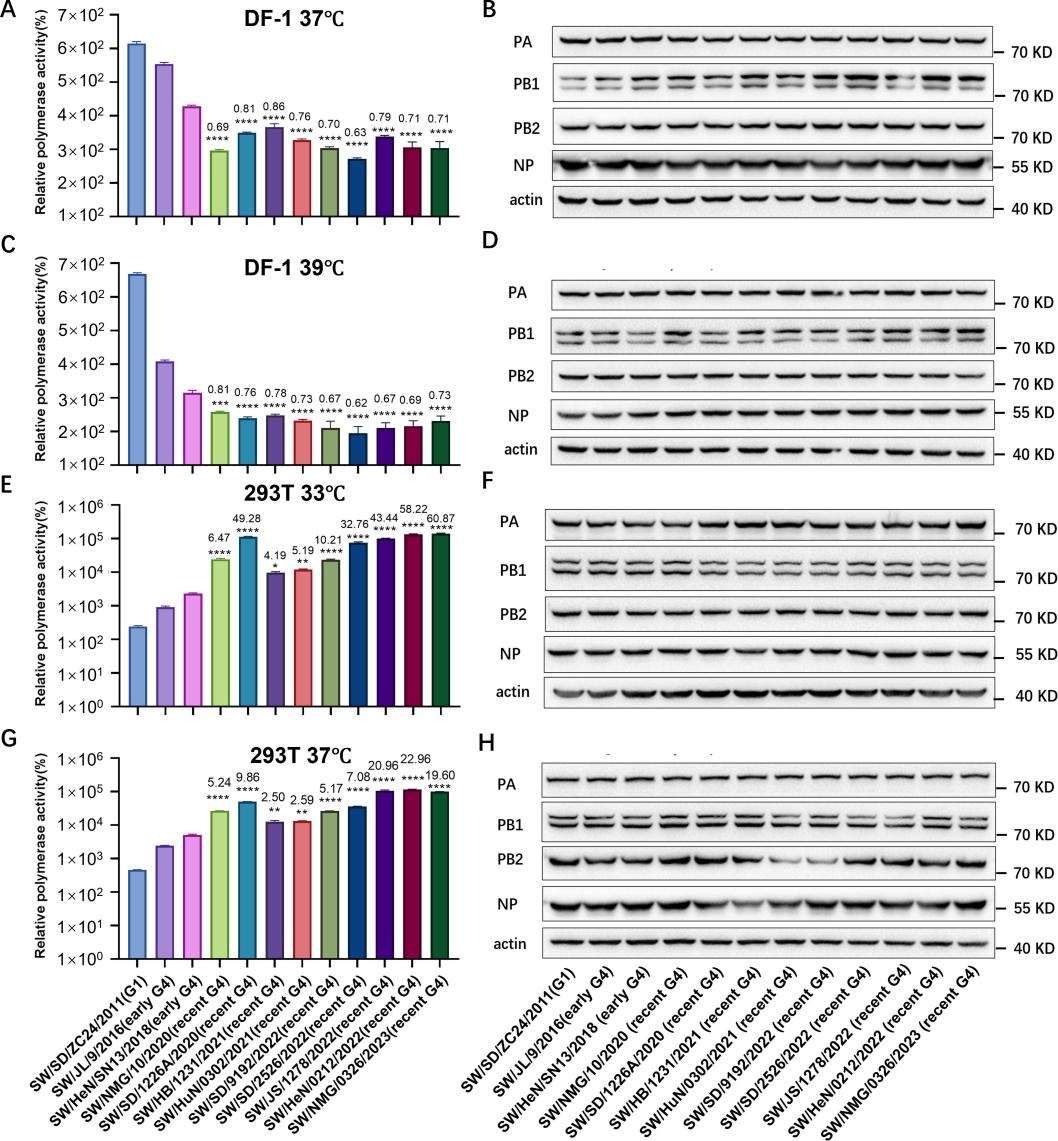
Polymerase activities of EA H1N1 viruses in DF-1 cells or HEK293T cells were determined by minigenome replication assays. Plasmids encoding PB2, PB1, PA, and NP proteins derived from G1 or G4 EA H1N1 viruses were transfected into DF-1 cells or HEK293T cells. At 24 h post-transfection, luciferase activities were measured. Levels of polymerase proteins were detected by western blotting. (**A through D**) Polymerase activities of EA H1N1 viruses in DF-1 cells at 37°C (**A and B**) and 39°C (**C and D**) (the temperature of the avian upper and lower respiratory tract, respectively). (**E through H**) Polymerase activities of EA H1N1 viruses in HEK293T cells at 33°C (**E and F**) and 37°C (**G and H**) (the temperature of the human upper and lower respiratory tract, respectively). Values are expressed as mean ± SD of three independent experiments. Data were analyzed by one-way ANOVA. ns, not significant; asterisks represent significant difference of polymerase activities (*P < 0.05, **P < 0.01, ***P < 0.001, and ****P < 0.0001) between recent G4 EA H1N1 virus and SW/HeN/SN13/2018 virus (this strain exhibited lower polymerase activity in DF-1 cells and higher polymerase activity in HEK293T cells than SW/JL/9/2016), and the number represents the fold changes.

The accumulation of multiple amino acid substitutions in pdm/09-derived vRNP genes may change the viral polymerase activity of G4 EA H1N1 viruses. To assess this, we performed minigenome replication assays in avian and mammalian cells. Based on the isolation time and phylogenetic topology, we selected nine representative viruses isolated from 2020 to 2023 (classified as recent G4 EA H1N1 virus) for further biological characterization (Table 1; Fig. S1). Two G4 EA H1N1 strains (SW/JL/9/2016 and SW/HeN/SN13/2018) isolated before 2019 (termed as early G4 EA H1N1 virus) and a G1 EA H1N1 virus (SW/SD/ZC24/2011, prototypical EA H1N1 virus, all genes originated from EA lineage) were selected for comparison. The results showed that the G1 EA H1N1 virus had higher polymerase activity than G4 EA H1N1 viruses in chicken embryo fibroblast DF-1 cells at 37℃ and 39℃, and the polymerase activity of recent G4 EA H1N1 strains was lower than the early G4 EA H1N1 viruses in DF-1 cells, indicating the viral polymerase activity of G4 EA H1N1 viruses in avian cells was decreased after 2020 (Fig. 3A through D). In contrast, the G1 EA H1N1 virus had the lowest polymerase activity in human embryonic kidney 293T (HEK293T) cells (Fig. 3E through H). Compared to the early strains, recent G4 EA H1N1 viruses isolated after 2020 exhibited a significant increase in polymerase activity in 293T cells (Fig. 3E through H). Specifically, the polymerase activities of these recent G4 EA H1N1 viruses in 293T cells were 4.19- to 60.87-fold higher than those of early G4 EA H1N1 strains at 33℃, and the fold increase ranged from 2.5 to 22.96 at 37°C (Fig. 3E through H). These results indicate that the accumulated mutations in the vRNP genes have progressively enhanced the polymerase activity of G4 EA H1N1 viruses in mammalian cells.

**TABLE 1:** Representative viruses used for analysis of biological characteristics^*a*^

Virus	Abbreviation	Genotype	Classification
A/swine/Shandong/ZC24/2011	SW/SD/ZC24/2011	G1 EA H1N1	Prototypical EA HAN1
A/swine/Jilin/9/2016	SW/JL/9/2016	G4 EA H1N1	Early G4 EA H1N1
A/swine/Henan/SN13/2018	SW/HeN/SN13/2018	G4 EA H1N1	Early G4 EA H1N1
A/swine/Neimenggu/10/2020	SW/NMG/10/2020	G4 EA H1N1	Recent G4 EA H1N1
A/swine/Shandong/1226A/2020	SW/SD/1226A/2020	G4 EA H1N1	Recent G4 EA H1N1
A/swine/Hebei/1231/2021	SW/HB/1231/2021	G4 EA H1N1	Recent G4 EA H1N1
A/swine/Hunan/0302/2021	SW/HuN/0302/2021	G4 EA H1N1	Recent G4 EA H1N1
A/swine/Shandong/9192/2022	SW/SD/9192/2022	G4 EA H1N1	Recent G4 EA H1N1
A/swine/Shandong/2526/2022	SW/SD/2526/2022	G4 EA H1N1	Recent G4 EA H1N1
A/swine/Jiangsu/1278/2022	SW/JS/1278/2022	G4 EA H1N1	Recent G4 EA H1N1
A/swine/Henan/0212/2022	SW/HeN/0212/2022	G4 EA H1N1	Recent G4 EA H1N1
A/swine/Neimenggu/0326/2023	SW/NMG/0326/2023	G4 EA H1N1	Recent G4 EA H1N1

^*a*^ Based on the isolation time and phylogenetic topology (Figure S1), we selected nine representative viruses isolated from 2020 to 2023 (classified as recent G4 EA H1N1 virus) for further biological characterization (Fig. S1). Two G4 EA H1N1 viruses isolated before 2019 (termed as early G4 EA H1N1 virus) and a G1 EA HAN1 virus (prototypical EA H1N1 virus, all genes originated from EA H1N1 lineage) were selected for comparison.

### G4 EA H1N1 viruses show increased infectivity and replication in mammalian cells

**Fig 4:**
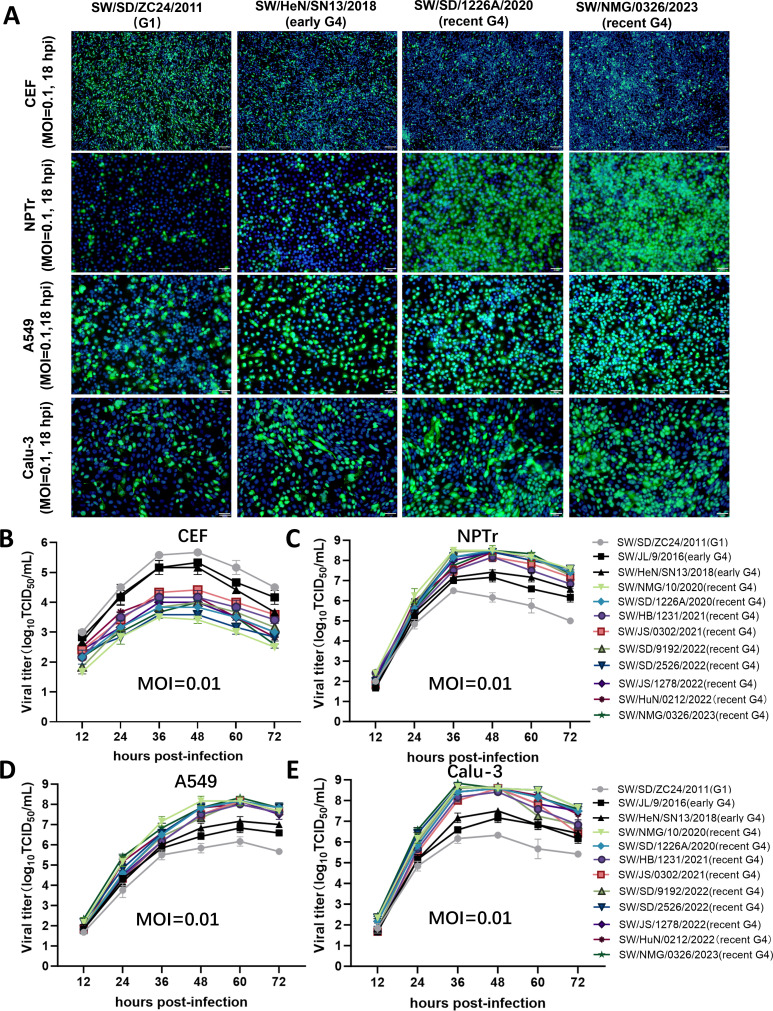
Infection and replication of EA H1N1 viruses in avian or mammalian cells. (A) CEFs, NPTr cells, A549 cells, and Calu-3 cells were infected with the indicated viruses at an MOI of 0.1 for 18 h. Influenza virus NP was detected at indicated time points with fluorescein isothiocyanate-conjugated goat anti-mouse antibody. Nuclei were detected with 4′,6-diamidino-2-phenylindole. Scale bars, 100 μm. A detailed figure and analysis of G4 EA H1N1 virus infection in avian or mammalian cells at 12, 18, and 24 h post-infection are shown in Fig. S3. (B–E) Growth kinetics of G4 EA H1N1 viruses were ascertained in CEFs (B), NPTr cells (C), A549 cells (D), and Calu-3 cells (E) infected with an MOI of 0.01. At indicated time points, cell culture supernatants were collected, and virus titers were determined by TCID_50_ assays on MDCK cells. A detailed significant difference in virus titers at each time point was presented in Fig. S4.

During the swIAV surveillance, all the viruses were successfully isolated using Madin-Darby canine kidney (MDCK) cells, whereas the virus isolation rate in embryonated chicken eggs was only 52% (22/42) after 2020 (Table S1). This finding, combined with the enhanced polymerase activity in mammalian cells and increased pathogenicity in swine (20, 21), suggested that the viral infectivity of the G4 EA H1N1 viruses to mammalian hosts may have changed. Thus, we assessed the infectivity of G4 EA H1N1 viruses in primary chicken embryo fibroblasts (CEFs), newborn pig trachea (NPTr) cells, and human alveolar epithelial cells (A549 and Calu-3) at a multiplicity of infection (MOI) of 0.1. The recent G4 EA H1N1 viruses had fewer infected CEFs than early G4 EA H1N1 strains at 18 hours post-infection (hpi) and 24 hpi (Fig. 4A; Fig. S3A through C and M). The highest percentages of influenza NP-positive CEFs in recent G4 EA H1N1 virus infection were 18.0% at 18 hpi and 28.3% at 24 hpi, while these numbers in early strains were over 42.2% at 18 hpi and over 46.2% at 24 hpi (Fig. 4A; Fig. S3A through C and M). The results showed a gradual decline in the infectivity of G4 EA H1N1 viruses in avian cells. In contrast, the percentages of NP-positive NPTr cells in the recent G4 EA H1N1 virus infection were over 71.6% at 18 hpi and over 93.69% at 24 hpi, significantly higher than those of early G4 EA H1N1 viruses (Fig. 4A; Fig. S3D through F and N). Similarly, these recent viruses also demonstrated enhanced infectivity in human cells. The rates of the NP-positive A549 cells infected by recent G4 EA H1N1 viruses were higher than those observed in early G4 EA H1N1 strains at 12, 18, and 24 hpi (Fig. 4A; Fig. S3G through I and O). In Calu-3 cells, three of the recent strains exhibited increased infectivity compared to the early G4 EA H1N1 strains at 18 hpi, while this trend expanded to all the recent G4 EA H1N1 viruses at 24 hpi (Fig. 4A; Fig. S3J through L and P). These results suggest that the recent G4 EA H1N1 viruses have acquired increased infectivity in mammalian cells.

To examine the replication dynamics of recent G4 EA H1N1 viruses, CEFs, NPTr, A549, and Calu-3 cells were separately infected by the indicated EA H1N1 virus at an MOI of 0.01. In CEFs, the recent G4 EA H1N1 viruses were replicated more poorly than early G4 EA H1N1 viruses, and the peak titers of recent G4 EA H1N1 viruses were lower than the early G4 EA H1N1 strains (Fig. 4B; Fig. S4A). On the contrary, recent G4 EA H1N1 viruses grew to higher levels in NPTr cells, producing more progeny viruses from 36 to 60 hpi than early G4 EA H1N1 viruses (Fig. 4C; Fig. S4B). In human A549 cells, all the recent G4 EA H1N1 viruses had higher titers than the early G4 EA H1N1 viruses at 60 hpi (Fig. 4D; Fig. S4C). Similarly, the recent G4 EA H1N1 viruses replicated more efficiently in Calu-3 cells compared to the early G4 EA H1N1 strains (Fig. 4E; Fig. S4D), as all the recent G4 EA H1N1 viruses reached the highest titer at 36 or 48 hpi and replicated more effectively than the early G4 EA H1N1 viruses at these time points (Fig. 4E; Fig. S4D). Taken together, these findings suggest that, following long-term circulation in the swine host, G4 EA H1N1 viruses have evolved to replicate more efficiently in both swine and human respiratory epithelial cells.

### G4 EA H1N1 viruses show enhanced pathogenicity in mice

**Fig 5:**
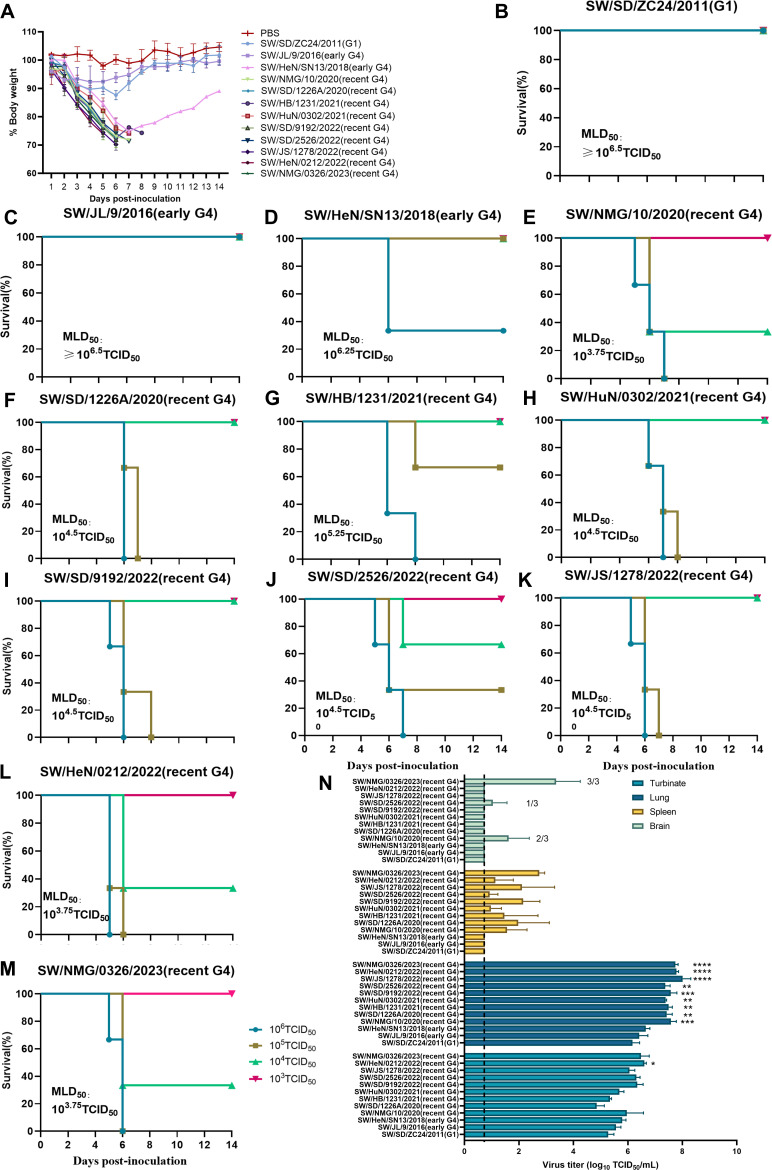
Pathogenicity and replication of EA H1N1 viruses in mice. (**A**) Groups of three mice were i.n. infected with indicated viruses at a dose of 10^6^ TCID_50_, and body weight changes were monitored for 14 days. (**B–M**) Groups of three mice were i.n. infected with indicated viruses at doses of 10^3^–10^6^ TCID_50_, and the survival of mice in each group was monitored for 14 days. (**N**) Mice were infected at 10^6^ TCID_50_ with indicated viruses, and three mice per group were euthanized at 4 dpi. Virus titers in different organs (turbinate, lung, brain, and spleen) were determined by TCID_50_ assay on MDCK cells. Data was analyzed by one-way ANOVA. Values are expressed as mean ± SD of three independent experiments. ns, not significant; asterisks represent significant difference (*P < 0.05, **P < 0.01, ***P < 0.001, and ****P < 0.0001) between recent G4 EA H1N1 virus-infected cells and SW/HeN/SN13/2018 (this early G4 EA H1N1 strain had higher virulence in mice than SW/JL/92016) virus-infected cells. Dashed lines indicate the lower limit of virus detection.

**Fig 6:**
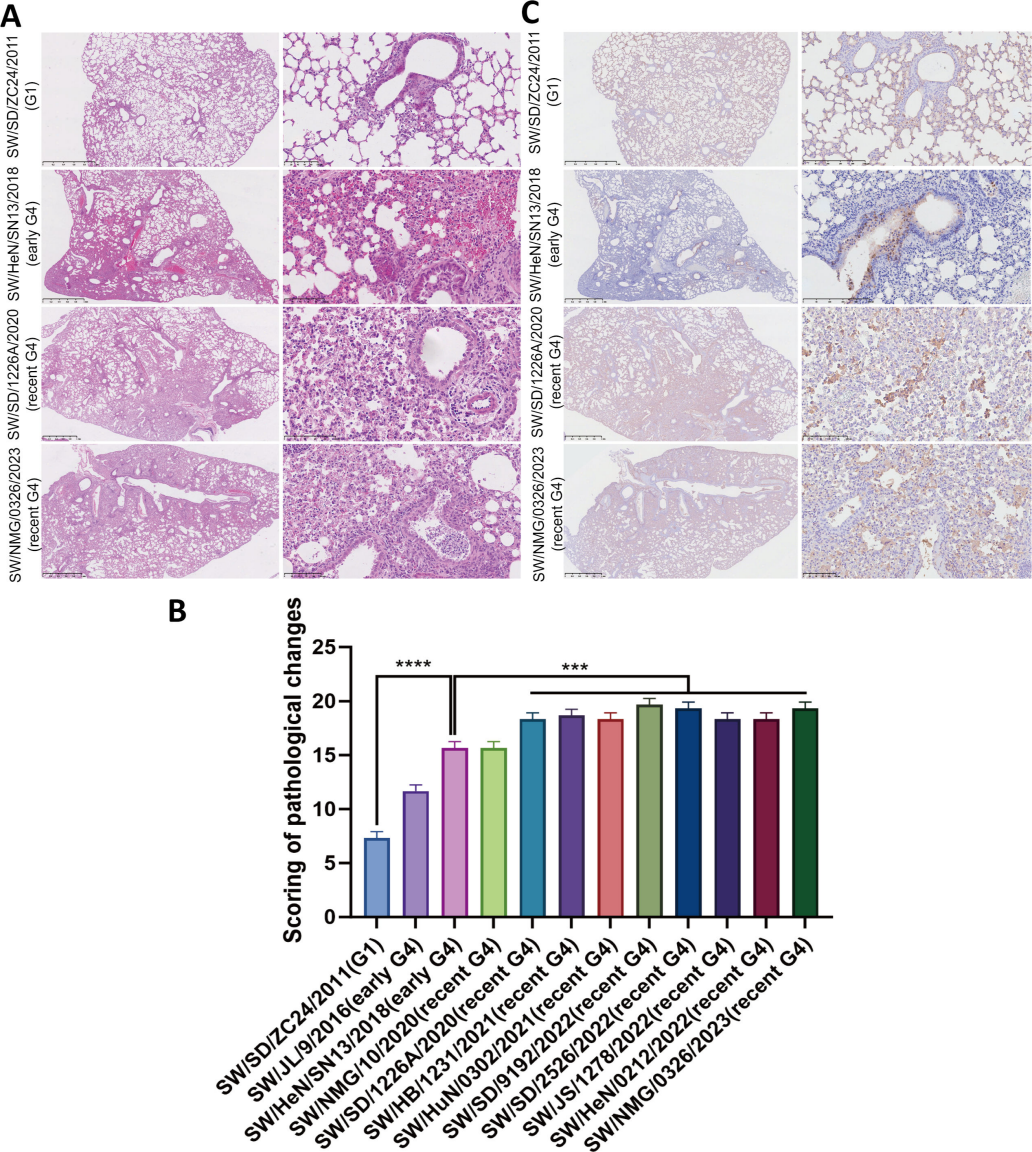
Histopathology in the lungs of EA H1N1 virus-infected mice. Representative histopathological findings in the lung of mice infected with the indicated viruses at 4 dpi. (**A**) The lung sections were stained with hematoxylin and eosin; recent G4 EA H1N1 viruses showed more extensive pulmonary consolidation, peribronchiolitis, and bronchopneumonia. (**B**) Lungs were evaluated for pathological lesions based on a scoring system comprising five parameters. (**C**) The lung sections were detected by immunohistochemistry against influenza viral NP antigen. Scale bars, 100 µm. Data were analyzed by one-way ANOVA. Values are expressed as mean ± SD of three independent scorings. ns, not significant; asterisks represent significant difference (*P < 0.05, **P < 0.01, ***P < 0.001, and ****P < 0.0001).

To evaluate the pathogenicity and replication ability of G4 EA H1N1 viruses in mammalian hosts, BALB/c mice were each intranasally (i.n.) infected with 10^3^–10^6^ 50% tissue culture infectious dose (TCID_50_) of the indicated virus in 50 µL volume and monitored daily over 14 days. More than 25% body weight loss was observed in two of three mice infected with 10⁶ TCID_50_ of the early G4 EA H1N1 virus SW/HeN/SN13/2018, as well as in all mice infected with 10⁶ TCID_50_ of nine recent G4 EA H1N1 viruses over the 14-day period (Fig. 5A). The mouse lethal dose at 50% (MLD_50_) of the recent G4 EA H1N1 viruses ranged from 10^5.25^ to 10^3.75^ TCID_50_, exhibiting higher virulence compared with the early G4 EA H1N1 strains (10^6.25^ TCID_50_) (Fig. 5B through M). To assess virus replication *in vivo*, mice were i.n. infected with 10^6^ TCID_50_ viruses, and turbinate, lung, brain, and spleen were collected for virus titration at 4 days post-inoculation (dpi). The results showed no significant difference in viral titers in the turbinate between early and recent G4 EA H1N1 viruses (Fig. 5N). However, recent G4 EA H1N1 viruses had exhibited higher virus titers (10^7.39^–10^8.01^TCID_50_/mL) in the lungs compared to the early G4 EA H1N1 strains (10^6.45^–10^6.65^TCID_50_/mL )(Fig. 5N), indicating an increased replication capacity of the G4 EA H1N1 viruses in the lower respiratory tract. Furthermore, the recent G4 EA H1N1 viruses were all detected in the spleen of infected mice, and three viruses (SW/NMG/10/2020, SW/SD/2526/2022, and SW/NMG/0326/2023) were found in the brain (Fig. 5N). Histopathological examination revealed that all the G4 EA H1N1 virus infections caused damage in the upper respiratory tract of mice, with the nasal epithelium dropped in the nasal cavity (Fig. S5A). In contrast, the pathological damage caused by the G4 EA H1N1 viruses in the lungs was gradually increased (Fig. S5B). Infection with recent G4 EA H1N1 viruses resulted in more extensive pulmonary consolidation, peribronchiolitis, and bronchopneumonia (characterized by edema and infiltration of inflammatory cells) and was associated with significantly higher histopathology scores compared to the early G4 EA H1N1 viruses (Fig. 6A and B; Fig. S5B). Abundant viral antigen-positive cells were also detected in the infected lung tissues (Fig. 6C; Fig. S5B). These results suggested that, during their long-term circulation in pigs, G4 EA H1N1 viruses have evolved increased pathogenicity in mice, as reflected by enhanced replication efficiency and more severe damage in lungs.

## DISCUSSION

SwIAVs play a crucial role in the emergence of novel human pandemic influenza virus (2). Previous studies have shown that the G4 EA H1N1 virus, the reassortant virus derived from EA H1N1 and pandemic pdm/09 H1N1 viruses, emerged as the predominant swIAVs circulating in China since 2016 (17, 18). Compared to the prototypical G1 EA H1N1 virus, the G4 EA H1N1 virus demonstrates more efficient replication in human airway epithelial cells, exhibits efficient transmissibility in the ferret model, and has a 10.4% seroprevalence among swine-exposed workers (18). This raises concerns about the potential risk of the G4 EA H1N1 virus causing a human pandemic. In this study, we find that G4 EA H1N1 viruses remained the predominant swIAVs in China from 2019 to 2023 and had reassorted with the co-circulated swIAVs, producing a novel H1N2 subtype virus. Time-scaled phylogenetic analysis showed that the pdm/09 lineage internal genes present in G4 EA H1N1 viruses were derived from reassortment events that occurred around 2009–2010. Since then, these genes have undergone continuous evolution in the swine host, with evolutionary rates higher than those observed in the human seasonal pdm/09 H1N1 viruses. The accelerated evolution of pdm/09-derived internal genes has significantly enhanced the polymerase activity of the G4 EA H1N1 viruses, resulting in increased replication efficiency in mammalian cells. The mouse model is widely used to study influenza viruses with pandemic potential (26). Previous studies using this model have revealed the pathogenicity of multiple swIAVs, including pdm/09 H1N1, TR lineage, and EA H1N1 viruses, and demonstrated its effectiveness in evaluating the infectivity and pathogenesis of swIAVs (15, 17, 26, 27). Therefore, we employed the mouse model to assess the *in vivo* pathogenicity of G4 EA H1N1 viruses and found that recent strains exhibited higher replication levels in mouse lungs compared to early G4 EA H1N1 strains, indicating increased pathogenicity of G4 EA H1N1 viruses in mice.

Since 2009, the pdm/09 H1N1 viruses have been frequently transmitted to swine herds and have reassorted with swIAVs around the world, generating a variety of reassortant viruses (8–10). In North America and Europe, the pdm/09 lineage internal genes in reassortant TR and EA lineage swIAVs originated from dynamic reassortment events following multiple reverse-zoonotic transmission of the human pdm/09 H1N1 viruses to swine (10, 16). In 2017, Meng et al. (24) also isolated a highly virulent EA H1N1 strain bearing internal genes from the pdm/09 H1N1 virus in the Guizhou Province of China and discovered that its PA gene carried mutations (V100I, N321K, I330V, and A639T) similar to those observed in the human seasonal pdm/09 H1N1 viruses. These studies suggest that frequent human-to-swine spillovers of seasonal pdm/09 H1N1 viruses may drive the genetic evolution of the internal genes in G4 EA H1N1 viruses. In the present study, genetic analysis has shown that the pdm/09-derived internal genes in G4 EA H1N1 viruses all originated from pdm/09 H1N1 viruses during 2009–2010. We found no evidence of reassortment with internal genes from human seasonal pdm/09 H1N1 viruses in our surveillance after 2013, indicating that the G4 EA H1N1 viruses currently circulating in swine populations have not experienced dynamic reassortment with human pdm/09 H1N1 viruses. Importantly, we identified two reassortant EA H1N2 viruses, which were generated through reassortment between the G4 EA H1N1 virus and the recent human-like H3N2 or TR lineage swIAV. A similar reassortant EA H1N2 virus and a novel H3N1 subtype virus, originating from the reassortments of the recent human-like H3N2 virus and the G4 EA H1N1 virus, have also been reported by others (19, 22). These findings suggest that G4 H1N1 viruses have reassorted with co-circulating swIAV lineages prevalent in swine populations. Beyond other swIAVs, the G4 EA H1N1 virus can also reassort with the incoming avian influenza virus, such as the H9N2 virus (28), which might enhance the pathogenicity and pandemic potential of these reassortant viruses when pdm/09 lineage internal genes from the G4 H1N1 virus are incorporated (29).

Swine serve as important intermediate hosts for the adaptation of IAVs to mammals. Swine possess both α2-3- and α2-6-linked sialosides on the surface of respiratory epithelial cells, allowing the avian influenza virus to shift its HA binding preference from avian to mammalian receptors. Su et al. (30) found that after the EA H1N1 virus transitioned from avian to swine hosts in 1979, its HA receptor binding specificity evolved from recognizing both α2-3- and α2-6-linked sialosides to exclusively recognizing α2-6-linked sialosides. Except for the receptors, circulating in swine could induce adaptive mutations in vRNP genes of the EA H1N1 virus and enable this virus to transmit efficiently in the host (30). By subjecting a recombinant virus with the same gene constellation as the pdm/09 H1N1 virus in swine, Wei et al. (31) also demonstrated that serial passage in swine could rapidly induce adaptive mutations in the internal genes of the recombinant virus, transforming it into a highly virulent and infectious form. Here, we observe that the evolutionary rates of pdm/09-derived internal genes in the G4 EA H1N1 viruses are faster than those of human seasonal pdm/09 H1N1 viruses. The continuous and accelerated evolution leads to greater genetic diversity in vRNP genes, thereby increasing the G4 EA H1N1 virus’s adaptation to the swine host (32). There are more amino acid mutations in the internal genes of the G4 EA H1N1 viruses, and they are different from those in human pdm/09 H1N1 viruses. These substitutions accumulate in multiple functional domains of the vRNP proteins, some of which have been identified as key regions for interactions between viral proteins and host factors (such as the C-terminal domain of PA and the 627 domain of PB2) (33, 34). The generation of pdm/09 H1N1 virus has evidenced that the continuous variation of vRNP genes under the interactions of swine host factors can drive the emergence of IAV with pandemic potential. Human ANP32A poorly supports the polymerase activity of avian influenza viruses, thereby limiting interspecies transmission of IAVs from birds to humans (35). However, the swine ANP32A supports the polymerase activity of avian influenza viruses, allowing the avian-origin PB2 gene to integrate into TR lineage swIAVs and acquire the compensatory mutations for PB2 E627K during circulation in swine, ultimately helping the reassortant virus pdm/09 H1N1 overcome the restriction of human ANP32A (2, 35–38). Moreover, myxovirus resistance protein A (MxA), encoded by the interferon-inducible *Mx1* gene, functions as an important antiviral restriction factor in humans against IAVs (39). The relatively low antiviral activity of swine MxA exerted weak selective pressure on the NP gene of CS lineage H1N1 viruses, leading to the development of partial resistance to human MxA before this segment was introduced into the pdm/09 H1N1 viruses (39–41). Such instances highlight the necessity to closely monitor key mutations in the internal genes of the G4 EA H1N1 viruses to evolve within swine populations. This knowledge will be used to guide more effective surveillance and control strategies for these viruses.

In summary, our findings show that the independent and accelerated evolution of pdm/09-derived internal genes within the swine host has enhanced both the infectivity and pathogenicity of the G4 EA H1N1 virus to mice. These G4 EA H1N1 viruses contribute to the emergence of novel reassortant viruses, thereby increasing the risk to human public health. Consequently, implementing continuous surveillance and proactive immunization strategies is essential to control these viruses. These steps are crucial for managing novel reassortant swIAVs and preventing future human pandemics.

## MATERIALS AND METHODS

### Cells and viruses

DF-1, MDCK, A549, HEK293T, and Calu-3 cells were purchased from American Type Culture Collection (ATCC) (Manassas, VA, USA), and NPTr cells were obtained from Istituto Zooprofilattico Sperimentale della Lombardia e dell'Emilia Romagna (Brescia, Italy). The CEFs were prepared from 9-day-old embryonated specific pathogen-free chicken eggs (Beijing Boehringer Ingelheim Vital Biotechnology Co, Ltd., Beijing, China). These cells were cultured with Dulbecco’s modified Eagle medium (Gibco, New York, USA) supplemented with 10% fetal bovine serum (Gibco, Sydney, Australia) and 1% penicillin-streptomycin solution (MACGENE, Beijing, China) at 37°C with 5% CO_2_. G1 EA H1N1 virus (A/swine/Shandong/ZC24/2011), G4 EA H1N1 viruses (A/swine/Jilin/9/2016, A/swine/Jiangsu/J006/2018 and A/swine/Henan/SN13/2018), and pdm/09 H1N1 virus (A/Beijing/21/2019) were kept by our laboratory. Virus stocks were grown in MDCK cells.

### Virus isolation, identification, and genomic sequencing

From January 2019 to April 2023, both active and passive influenza surveillance in pigs was conducted in seven provinces of China (Henan, Hebei, Hunan, Jiangsu, Shandong, Liaoning, and Neimenggu). A total of 7,679 samples, including nasal swabs from slaughtered pigs or lung tissues from farmed pigs with signs of respiratory disease, were collected. For virus isolation, nasal swabs were taken and placed in 1.0 mL transmission medium (50% [vol/vol] glycerol in phosphate-buffered saline [PBS]) containing antibiotics. Lungs were homogenized in PBS containing antibiotics. All samples were individually inoculated into both embryonated chicken eggs and MDCK cells for virus isolation. Allantoic fluid and culture supernatants were harvested and tested for HA activity.

Viral RNA was extracted by using the QIAamp Viral RNA Minikit (Qiagen, Hilden, Germany), and standard reverse transcriptase PCR (RT-PCR) was performed with segment-specific primers, using the One Step RT-PCR Kit (Takara, Beijing, China). The HA and NA lineages of the viruses were determined by direct sequencing of the PCR products. Samples positive for swIAVs were stored as stock at −80°C until further analysis. All viruses isolated in this study were subjected to full genome sequencing on an ABI ^3730^ genetic analyzer (Applied Biosystems).

### Phylogenetic analyses

All the available reference sequences of multiple lineages of swIAVs were downloaded from databases of the National Center for Biotechnology Information (NCBI) (https://www.ncbi.nlm.nih.gov/genomes/FLU/) and the Global Initiative on Sharing All Influenza Data (GISAID) (https://www.gisaid.org/). We aligned gene segments individually by using MAFFT version 7.27 (https://mafft.cbrc.jp/alignment/ software/) and further edited the data using the MEGA 7.0 version (https://www.megasoftware.net/). IQ-TREE 2.2.0 (http://www.iqtree.org) was used to construct maximum likelihood phylogenies for each segment. The genotypes of swIAVs isolated in this study were determined by assigning each gene segment to specific lineages based on phylogenetic trees constructed using the GTRGAMMA+I nucleotide substitution model as described in a previous study (18). One thousand bootstrap replicates were run to assess the reliability of the trees.

To investigate the reassortment dynamics of pdm/09 lineage internal genes in the swine host, the time-scaled phylogenetic trees based on the internal genes (PB2, PB1, PA, NP, and M) of G4 EA H1N1 viruses and human pdm/09 H1N1 viruses were constructed. All the H1N1 swIAV sequences from 2013 to 2023 downloaded from NCBI and GISAID databases were used for phylogenetic analysis, through which a total of 256 G4 EA H1N1 viruses were identified. These, together with the 34 G4 EA H1N1 viruses identified in this study, were included in further analysis. Similarly, a total of 879 human pdm/09 H1N1 virus sequences collected between 2009 and 2023 were also downloaded from databases. To mitigate sampling bias and overrepresentation of specific traits while preserving genetic diversity in the data set, we used SAMPI (https://github.com/jlcherry/SAMPI) to obtain a homogeneous collection of samples based on region and collection date, while maintaining a manageable data set size for computational efficiency. The time to TMRCA of the PB2, PB1, PA, NP, and M genes of G4 EA H1N1 viruses was estimated using BEAST version 1.10.4 under a molecular clock model based on time-scaled phylogenetic analyses, including swIAVs and the human pdm/09 H1N1 virus (42). Phylogeny was estimated within a Bayesian Markov Chain Monte Carlo (MCMC) framework using the GTR substitution model with gamma-distributed among-site rate heterogeneity and a relaxed uncorrelated lognormal molecular clock model, following previously described methods for swIAVs analysis (43, 44). MCMC chains were run for 200 million iterations and sampled every 20,000 steps to make sure that all parameters converged (effective sample size values greater than 200), and the first 10% of samples were discarded as burn-in. A maximum clade credibility (MCC) tree was generated and summarized by TreeAnnotator (version 1.10.4). The ESS values of the main traces according to Tracer (joint, prior, likelihood, treeModel.rootHeight, age [root], and treeLength), along with the number of trees used to construct the MCC trees, are presented in Table S5.

### Average nucleotide variation analysis

To assess the evolutionary rates of the internal genes of G4 EA H1N1 swIAVs and human pdm/09 H1N1 viruses, average nucleotide variation frequencies of these two lineage viruses were compared. We also used SAMPI to obtain a representative yet computationally manageable data set from 290 swine viruses or 879 human viruses as described above. Further analysis employed a GTR substitution model with an uncorrelated relaxed clock and a constant GMRF Bayesian Skyride tree prior as reported (45). The MCMC chain was run for 200 million generations, sampling every 20,000 steps and discarding 10% as burn-in. Tracer version 1.7, implemented in BEAST, was used to check the analysis for convergence. The results of three runs were combined using the LogCombiner version 1.8.0.

### Amino acid variation analysis

The PB2, PB1, PA, NP, and M genes of the G4 EA H1N1 viruses and human pdm/09 H1N1 viruses were translated into amino acids using Mega version 7.0. The amino acid sites that differ from the reference virus (A/California/04/2009) were marked. The pheatmap package in R was used to generate a heatmap.

### Antigenic analyses

The antigenic characterization of representative EA H1N1 viruses was compared using HI assays. Ferret antisera against the pdm/09 H1N1 virus (A/Beijing/21/2019), G1 EA H1N1 virus (A/Swine/Shandong/ZC24/2011), and G4 EA H1N1 viruses (A/swine/Jiangsu/J006/2018 and A/swine/Henan/SN13/2018) were produced as previously described (18). Briefly, 6-month-old male ferrets, serologically negative for currently circulating influenza viruses and >1.0 kg (ranging from 1.10 to 1.80 kg) in weight, were purchased from Angora LTD (Jiangsu, China). Ferrets were anesthetized with ketamine (20 mg/kg of body weight, Hospira, Melbourne, Australia) and xylazine (1 mg/kg of body weight, Merck, Darmstadt, Germany) and i.n. inoculated with 10^6^ TCID_50_ of the indicated virus diluted in 0.5 mL volume PBS. Three weeks later, ferrets were euthanized, and antisera were collected for HI assays. HI tests were performed in accordance with WHO guidelines as described previously (46). Briefly, ferret antisera were pretreated with receptor-destroying enzyme ([RDE], Seiken, Japan) (antisera: RDE = 1:4) and incubated together for 18 h at 37°C, then transferred to 56°C for 30 min. RDE-treated antisera in twofold dilutions starting at 1:10 and four HA units of indicated viruses were tested in V-bottom 96-well reaction plates with 1% chicken red blood cells. Assays were done in duplicate.

### Infection and growth kinetics of viruses in avian or mammalian cells

The CEFs, NPTr, A549, and Calu-3 cells were infected with indicated viruses at an MOI of 0.1. The inoculum was removed after 1 h, and cells were further incubated at 37°C. The culture cells were fixed in 4% formaldehyde (Beyotime, Beijing, China) at 12, 18, or 24 hpi. After fixation of 30 min, the cultures were permeabilized with 0.5% Triton X-100 (Beyotime, Beijing, China) for 30 min, incubated with blocking buffer (Beyotime, Beijing, China) for 30 min at 37°C, and immunostained with mouse anti-NP IgG antibody (1:5,000; ab20343, Abcam, Beijing, China) at 4°C overnight. The cultures were then incubated with fluorescein-isothiocyanate-labeled goat anti-mouse IgG antibody (1:400; 5230-0427, KPL, Gaithersburg, USA) and co-stained with 4′,6-diamidino-2-phenylindole solution (Solarbio, Beijing, China). Then, cell cultures were imaged using a fluorescence microscope (Ti–U-Nikon, Tokyo, Japan) with a video documentation system.

To assess the growth kinetics of viruses, CEFs, NPTr, A549, and Calu-3 cells were cultured in 6-well plates and infected with indicated viruses at an MOI of 0.01. After a 2-h incubation period, the cells were washed three times with PBS and incubated at 37°C in 5% CO_2_. Supernatants were collected at 12, 24, 36, 48, 60, and 72 hpi, and viral titers were determined by TCID_50_ assays.

### Mouse challenge studies

Groups of three 6-week-old female BALB/c mice were anesthetized with Zoletil 50 (tiletamine-zolazepam [Virbac, Carros, France], 20 μg/g of body weight) and i.n. inoculated with 50 µL of the indicated virus in 10-fold serial dilutions from 10^3^ to 10^6^ TCID_50_. The mice in each group were monitored daily for 14 days for weight loss and mortality to determine MLD_50_. Mice that lost > 25% of their initial body weight were euthanized. MLD_50_ values were calculated according to the Reed and Muench method. To monitor virus replication, three mice infected with 10^6^ TCID_50_ viruses from each group were euthanized at 4 dpi. Nasal turbinate, lung, brain, and spleen samples were collected for virus titration. The turbinate and lung tissues were also used for histopathological examination.

### Histopathology and immunohistochemistry analysis

Tissues were fixed in 10% buffered formalin, embedded in paraffin, sectioned, and stained with hematoxylin and eosin. Pathological changes in the sections of the lung were scored by a veterinary pathologist blinded to the infected group. Five parameters were each scored on a five-point scale of 0–4 and then summed to give a total slide score ranging from 0 to 20. Scoring criteria (Table S6) were based on a previously published method with modifications (47).

The tissue sections were also deparaffinized with xylene and rehydrated with ethanol gradients and water. Endogenous peroxidase activity was blocked using 3% hydrogen peroxide in methanol. PBS containing 0.05% Tween-20 was used to wash lung tissue sections between steps. Lung sections were incubated with a mouse monoclonal primary antibody (AA5H; Abcam, Cambridge, UK) at 1:500 dilution at 4°C overnight in a humidified chamber. Sections were subsequently incubated with the HRP-conjugated goat anti-mouse secondary antibody (12-349; Millipore, Billerica, MA, USA) for 60 min at RT. Immunodetection was performed using an HRP reaction kit (diaminobenzidine tetrahydrochloride; Sigma, St. Louis, MO, USA).

### Polymerase activity assay

Viral polymerase activity was determined using a luciferase reporting assay as previously described (31). Briefly, the PB2, PB1, NP, and PA genes from the indicated virus were separately cloned into the pcDNA3.1(+) plasmid. DF-1 cells or HEK293T cells were co-transfected with PB2, PB1, NP, and PA expression plasmids (125 ng), together with the firefly luciferase reporter plasmid p-Luci (10 ng), and internal control pRL-TK (2.5 ng) at the indicated temperature, using JetPRIME (Polyplus, New York, USA) according to the manufacturer’s instructions. At 24 h post-transfection, cell lysates were prepared using the Dual-Luciferase Reporter Assay System (Promega, Madison, USA), and the luciferase activities were measured on a GloMax 96 microplate luminometer. Polymerase proteins were detected by western blotting. Three independent experiments were performed.

### Statistical analysis

Image J software was used for quantitative image analysis. All statistical analyses were performed using GraphPad Prism software version 8.0 (GraphPad Software Inc., San Diego, CA, USA). A one-way analysis of variance was used for comparisons among multiple groups, whereas a two-way ANOVA was used when two independent factors were involved. Differences were considered statistically significant at a *P*-value of < 0.05.

## Data Availability

The sequences generated in this study have been deposited in the GISAID database, with their accession numbers listed in Table S7. The accession numbers of the sequences used in the time-scaled phylogenetic analyses are provided in Table S8.

## SUPPLEMENTAL MATERIAL

Fig. S1 and S2 jvi.00430-25-s0001.pdf Evolution of reassortant Eurasian avian-like H1N1 virus.

Fig. S3 jvi.00430-25-s0002.pdf Characteristics of reassortant Eurasian avian-like H1N1 virus.

Additional supplemental material jvi.00430-25-s0003.pdf Figures S4 and S5; Tables S1 to S4.

Tables S5 and S6 jvi.00430-25-s0004.pdf Parameters for Bayesian molecular clock analysis and parameters used for scoring of histopathology lesions in lung.

Tables S7 and S8 jvi.00430-25-s0005.xlsx Accession numbers of sequences generated in this study and the sequences used in time-scaled phylogenetic analyses.

ASM does not own the copyrights to Supplemental Material that may be linked to, or accessed through, an article. The authors have granted ASM a non-exclusive, world-wide license to publish the Supplemental Material files. Please contact the corresponding author directly for reuse.
