# Supplementary material for: Continuous evolution of Eurasian avian-like H1N1 swine influenza viruses with pdm/09-derived internal genes enhances pathogenicity in mice
Source: J Virol. 2025 Sep 8;99(10):e00430-25. doi: 10.1128/jvi.00430-25 (PMC12548388; doi:10.1128/jvi.00430-25)
Supplement: Fig. S1 and S2 — Evolution of reassortant Eurasian avian-like H1N1 virus. [file jvi.00430-25-s0001.pdf]

Supplementary Materials

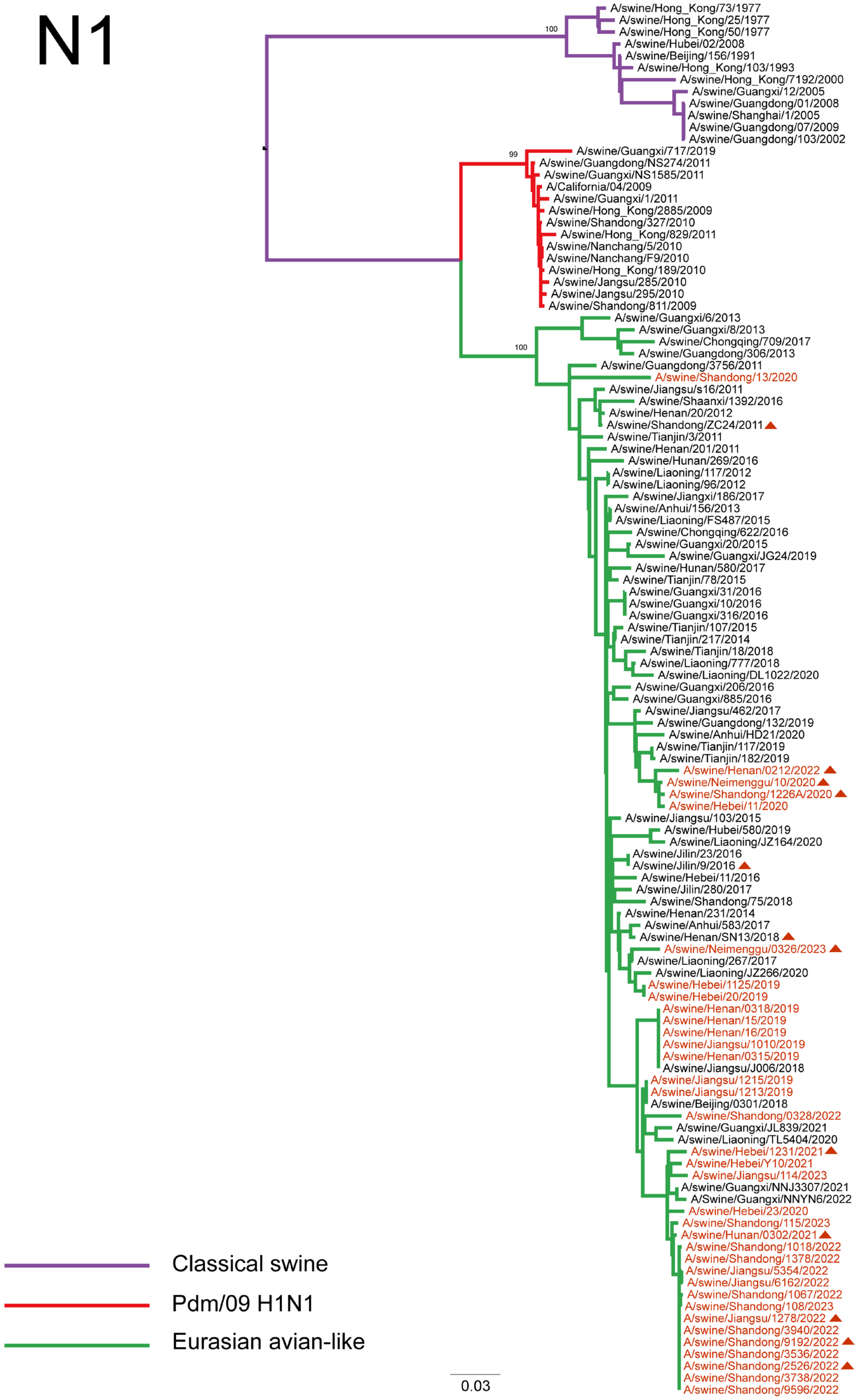

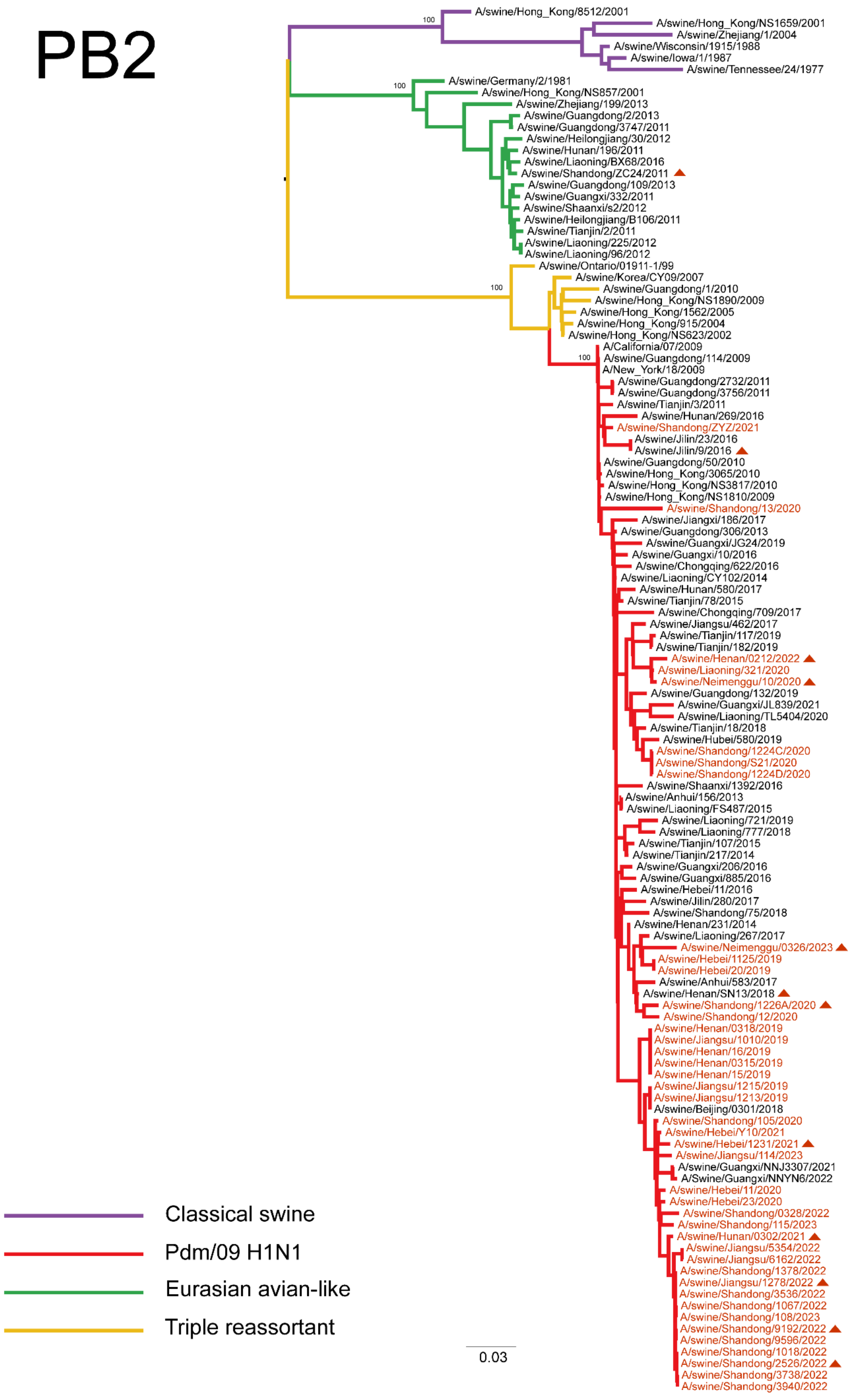
PB2
100
A/swine/Hong_Kong/8512/2001
A/swine/Hong_Kong/NS1659/2001
A/swine/Zhejiang/1/2004
A/swine/Wisconsin/1915/1988
A/swine/Iowa/1/1987
A/swine/Tennessee/24/1977
100
A/swine/Germany/2/1981
A/swine/Hong_Kong/NS857/2001
A/swine/Zhejiang/199/2013
A/swine/Guangdong/2/2013
A/swine/Guangdong/3747/2011
A/swine/Heilongjiang/30/2012
A/swine/Hunan/196/2011
A/swine/Liaoning/BX68/2016
A/swine/Shandong/ZC24/2011
A/swine/Guangdong/109/2013
A/swine/Guangxi/332/2011
A/swine/Shaanxi/s2/2012
A/swine/Heilongjiang/B106/2011
A/swine/Tianjin/2/2011
A/swine/Liaoning/225/2012
A/swine/Liaoning/96/2012
100
A/swine/Ontario/01911-1/99
A/swine/Korea/CY09/2007
A/swine/Guangdong/1/2010
A/swine/Hong_Kong/NS1890/2009
A/swine/Hong_Kong/1562/2005
A/swine/Hong_Kong/915/2004
A/swine/Hong_Kong/NS623/2002
100
A/California/07/2009
A/swine/Guangdong/114/2009
A/New_York/18/2009
A/swine/Guangdong/2732/2011
A/swine/Guangdong/3756/2011
A/swine/Tianjin/3/2011
A/swine/Hunan/269/2016
A/swine/Shandong/ZYZ/2021
A/swine/Jilin/23/2016
A/swine/Jilin/9/2016
A/swine/Guangdong/50/2010
A/swine/Hong_Kong/3065/2010
A/swine/Hong_Kong/NS3817/2010
A/swine/Hong_Kong/NS1810/2009
A/swine/Shandong/13/2020
A/swine/Jiangxi/186/2017
A/swine/Guangdong/306/2013
A/swine/Guangxi/JG24/2019
A/swine/Guangxi/10/2016
A/swine/Chongqing/622/2016
A/swine/Liaoning/CY102/2014
A/swine/Hunan/580/2017
A/swine/Tianjin/78/2015
A/swine/Chongqing/709/2017
A/swine/Jiangsu/462/2017
A/swine/Tianjin/117/2019
A/swine/Tianjin/182/2019
A/swine/Henan/0212/2022
A/swine/Liaoning/321/2020
A/swine/Neimenggu/10/2020
A/swine/Guangdong/132/2019
A/swine/Guangxi/JL839/2021
A/swine/Liaoning/TL5404/2020
A/swine/Tianjin/18/2018
A/swine/Hubei/580/2019
A/swine/Shandong/1224C/2020
A/swine/Shandong/S21/2020
A/swine/Shandong/1224D/2020
A/swine/Shaanxi/1392/2016
A/swine/Anhui/156/2013
A/swine/Liaoning/FS487/2015
A/swine/Liaoning/721/2019
A/swine/Liaoning/777/2018
A/swine/Tianjin/107/2015
A/swine/Tianjin/217/2014
A/swine/Guangxi/206/2016
A/swine/Guangxi/885/2016
A/swine/Hebei/11/2016
A/swine/Jilin/280/2017
A/swine/Shandong/75/2018
A/swine/Henan/231/2014
A/swine/Liaoning/267/2017
A/swine/Neimenggu/0326/2023
A/swine/Hebei/1125/2019
A/swine/Hebei/20/2019
A/swine/Anhui/583/2017
A/swine/Henan/SN13/2018
A/swine/Shandong/1226A/2020
A/swine/Shandong/12/2020
A/swine/Henan/0318/2019
A/swine/Jiangsu/1010/2019
A/swine/Henan/16/2019
A/swine/Henan/0315/2019
A/swine/Henan/15/2019
A/swine/Jiangsu/1215/2019
A/swine/Jiangsu/1213/2019
A/swine/Beijing/0301/2018
A/swine/Shandong/105/2020
A/swine/Hebei/Y10/2021
A/swine/Hebei/1231/2021
A/swine/Jiangsu/114/2023
A/swine/Guangxi/NNJ3307/2021
A/Swine/Guangxi/NNYN6/2022
A/swine/Hebei/11/2020
A/swine/Hebei/23/2020
A/swine/Shandong/0328/2022
A/swine/Shandong/115/2023
A/swine/Hunan/0302/2021
A/swine/Jiangsu/5354/2022
A/swine/Jiangsu/6162/2022
A/swine/Shandong/1378/2022
A/swine/Jiangsu/1278/2022
A/swine/Shandong/3536/2022
A/swine/Shandong/1067/2022
A/swine/Shandong/108/2023
A/swine/Shandong/9192/2022
A/swine/Shandong/9596/2022
A/swine/Shandong/1018/2022
A/swine/Shandong/2526/2022
A/swine/Shandong/3738/2022
A/swine/Shandong/3940/2022
Classical swine
Pdm/09 H1N1
Eurasian avian-like
Triple reassortant
0.03

PB1

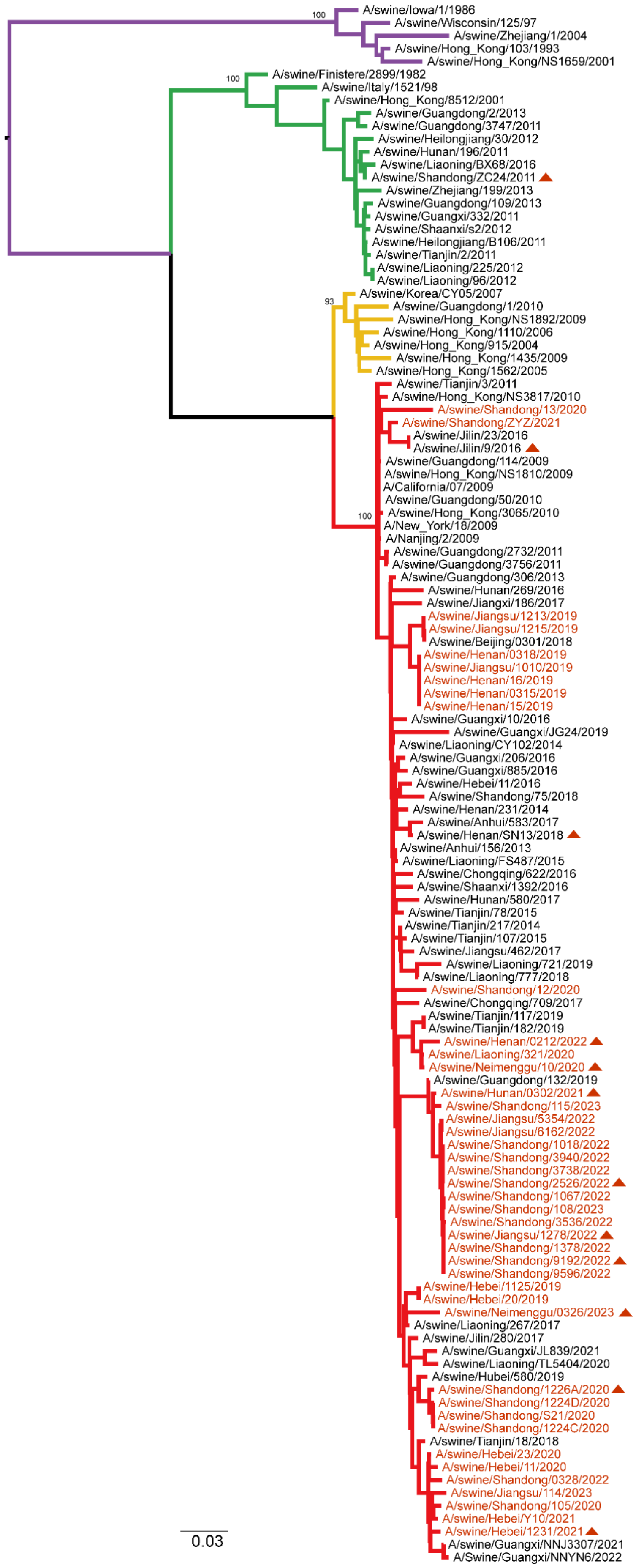

100
A/swine/Iowa/1/1986
A/swine/Wisconsin/125/97
A/swine/Zhejiang/1/2004
A/swine/Hong_Kong/103/1993
A/swine/Hong_Kong/NS1659/2001
100
A/swine/Finistere/2899/1982
A/swine/Italy/1521/98
A/swine/Hong_Kong/8512/2001
A/swine/Guangdong/2/2013
A/swine/Guangdong/3747/2011
A/swine/Heilongjiang/30/2012
A/swine/Hunan/196/2011
A/swine/Liaoning/BX68/2016
A/swine/Shandong/ZC24/2011
A/swine/Zhejiang/199/2013
A/swine/Guangdong/109/2013
A/swine/Guangxi/332/2011
A/swine/Shaanxi/s2/2012
A/swine/Heilongjiang/B106/2011
A/swine/Tianjin/2/2011
A/swine/Liaoning/225/2012
A/swine/Liaoning/96/2012
93
A/swine/Korea/CY05/2007
A/swine/Guangdong/1/2010
A/swine/Hong_Kong/NS1892/2009
A/swine/Hong_Kong/1110/2006
A/swine/Hong_Kong/915/2004
A/swine/Hong_Kong/1435/2009
A/swine/Hong_Kong/1562/2005
A/swine/Tianjin/3/2011
A/swine/Hong_Kong/NS3817/2010
A/swine/Shandong/13/2020
A/swine/Shandong/ZYZ/2021
A/swine/Jilin/23/2016
A/swine/Jilin/9/2016
A/swine/Guangdong/114/2009
A/swine/Hong_Kong/NS1810/2009
A/California/07/2009
A/swine/Guangdong/50/2010
100
A/swine/Hong_Kong/3065/2010
A/New_York/18/2009
A/Nanjing/2/2009
A/swine/Guangdong/2732/2011
A/swine/Guangdong/3756/2011
A/swine/Guangdong/306/2013
A/swine/Hunan/269/2016
A/swine/Jiangxi/186/2017
A/swine/Jiangsu/1213/2019
A/swine/Jiangsu/1215/2019
A/swine/Beijing/0301/2018
A/swine/Henan/0318/2019
A/swine/Jiangsu/1010/2019
A/swine/Henan/16/2019
A/swine/Henan/0315/2019
A/swine/Henan/15/2019
A/swine/Guangxi/10/2016
A/swine/Guangxi/JG24/2019
A/swine/Liaoning/CY102/2014
A/swine/Guangxi/206/2016
A/swine/Guangxi/885/2016
A/swine/Hebei/11/2016
A/swine/Shandong/75/2018
A/swine/Henan/231/2014
A/swine/Anhui/583/2017
A/swine/Henan/SN13/2018
A/swine/Anhui/156/2013
A/swine/Liaoning/FS487/2015
A/swine/Chongqing/622/2016
A/swine/Shaanxi/1392/2016
A/swine/Hunan/580/2017
A/swine/Tianjin/78/2015
A/swine/Tianjin/217/2014
A/swine/Tianjin/107/2015
A/swine/Jiangsu/462/2017
A/swine/Liaoning/721/2019
A/swine/Liaoning/777/2018
A/swine/Shandong/12/2020
A/swine/Chongqing/709/2017
A/swine/Tianjin/117/2019
A/swine/Tianjin/182/2019
A/swine/Henan/0212/2022
A/swine/Liaoning/321/2020
A/swine/Neimenggu/10/2020
A/swine/Guangdong/132/2019
A/swine/Hunan/0302/2021
A/swine/Shandong/115/2023
A/swine/Jiangsu/5354/2022
A/swine/Jiangsu/6162/2022
A/swine/Shandong/1018/2022
A/swine/Shandong/3940/2022
A/swine/Shandong/3738/2022
A/swine/Shandong/2526/2022
A/swine/Shandong/1067/2022
A/swine/Shandong/108/2023
A/swine/Shandong/3536/2022
A/swine/Jiangsu/1278/2022
A/swine/Shandong/1378/2022
A/swine/Shandong/9192/2022
A/swine/Shandong/9596/2022
A/swine/Hebei/1125/2019
A/swine/Hebei/20/2019
A/swine/Neimenggu/0326/2023
A/swine/Liaoning/267/2017
A/swine/Jilin/280/2017
A/swine/Guangxi/JL839/2021
A/swine/Liaoning/TL5404/2020
A/swine/Hubei/580/2019
A/swine/Shandong/1226A/2020
A/swine/Shandong/1224D/2020
A/swine/Shandong/S21/2020
A/swine/Shandong/1224C/2020
A/swine/Tianjin/18/2018
A/swine/Hebei/23/2020
A/swine/Hebei/11/2020
A/swine/Shandong/0328/2022
A/swine/Jiangsu/114/2023
A/swine/Shandong/105/2020
A/swine/Hebei/Y10/2021
A/swine/Hebei/1231/2021
A/swine/Guangxi/NNJ3307/2021
A/Swine/Guangxi/NNYN6/2022
0.03

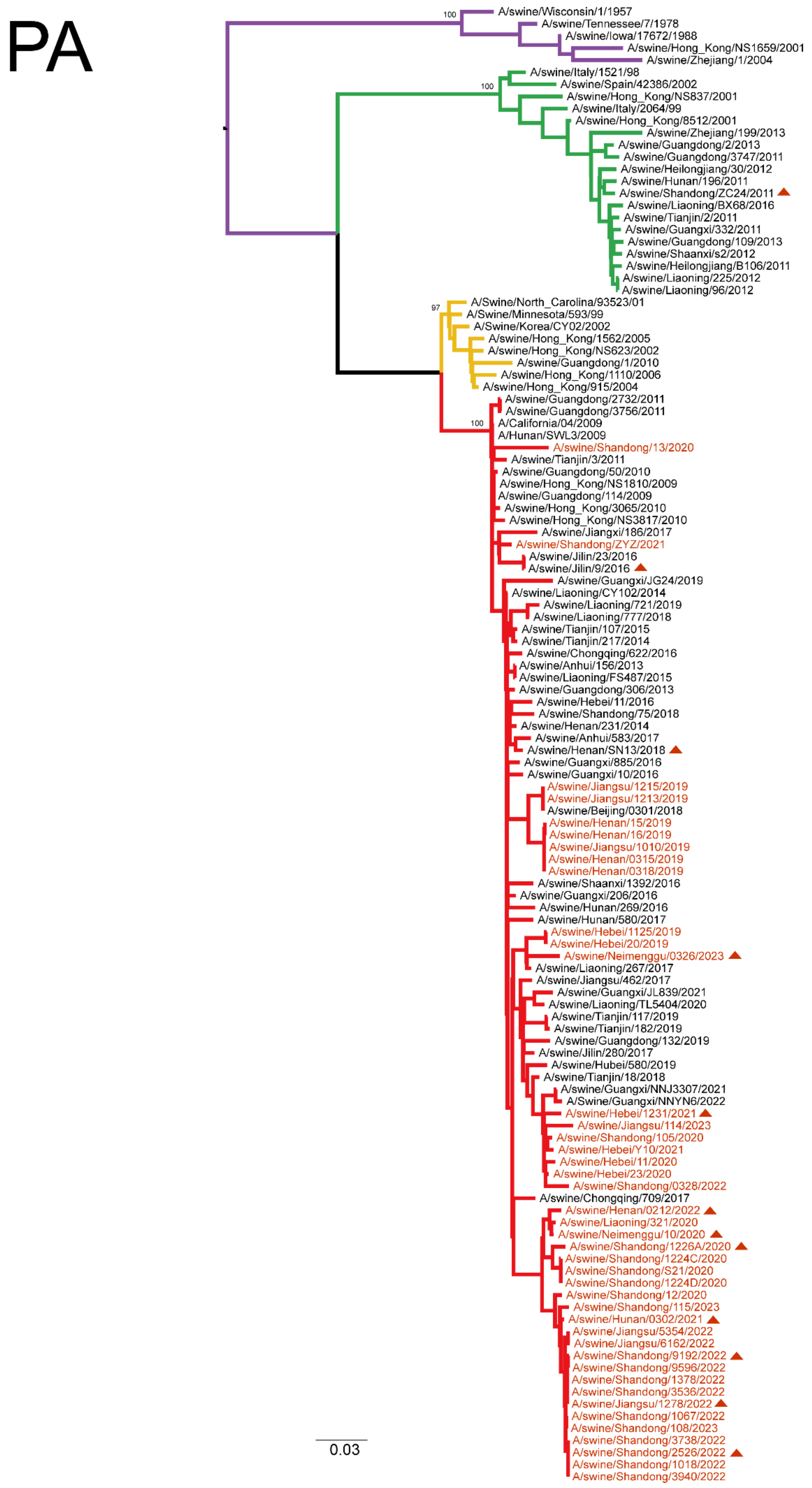
PA
100
A/swine/Wisconsin/1/1957
A/swine/Tennessee/7/1978
A/swine/Iowa/17672/1988
A/swine/Hong_Kong/NS1659/2001
A/swine/Zhejiang/1/2004
100
A/swine/Italy/1521/98
A/swine/Spain/42386/2002
A/swine/Hong_Kong/NS837/2001
A/swine/Italy/2064/99
A/swine/Hong_Kong/8512/2001
A/swine/Zhejiang/199/2013
A/swine/Guangdong/2/2013
A/swine/Guangdong/3747/2011
A/swine/Heilongjiang/30/2012
A/swine/Hunan/196/2011
A/swine/Shandong/ZC24/2011
A/swine/Liaoning/BX68/2016
A/swine/Tianjin/2/2011
A/swine/Guangxi/332/2011
A/swine/Guangdong/109/2013
A/swine/Shaanxi/s2/2012
A/swine/Heilongjiang/B106/2011
A/swine/Liaoning/225/2012
A/swine/Liaoning/96/2012
97
A/Swine/North_Carolina/93523/01
A/Swine/Minnesota/593/99
A/Swine/Korea/CY02/2002
A/swine/Hong_Kong/1562/2005
A/swine/Hong_Kong/NS623/2002
A/swine/Guangdong/1/2010
A/swine/Hong_Kong/1110/2006
A/swine/Hong_Kong/915/2004
A/swine/Guangdong/2732/2011
A/swine/Guangdong/3756/2011
100
A/California/04/2009
A/Hunan/SWL3/2009
A/swine/Shandong/13/2020
A/swine/Tianjin/3/2011
A/swine/Guangdong/50/2010
A/swine/Hong_Kong/NS1810/2009
A/swine/Guangdong/114/2009
A/swine/Hong_Kong/3065/2010
A/swine/Hong_Kong/NS3817/2010
A/swine/Jiangxi/186/2017
A/swine/Shandong/ZYZ/2021
A/swine/Jilin/23/2016
A/swine/Jilin/9/2016
A/swine/Guangxi/JG24/2019
A/swine/Liaoning/CY102/2014
A/swine/Liaoning/721/2019
A/swine/Liaoning/777/2018
A/swine/Tianjin/107/2015
A/swine/Tianjin/217/2014
A/swine/Chongqing/622/2016
A/swine/Anhui/156/2013
A/swine/Liaoning/FS487/2015
A/swine/Guangdong/306/2013
A/swine/Hebei/11/2016
A/swine/Shandong/75/2018
A/swine/Henan/231/2014
A/swine/Anhui/583/2017
A/swine/Henan/SN13/2018
A/swine/Guangxi/885/2016
A/swine/Guangxi/10/2016
A/swine/Jiangsu/1215/2019
A/swine/Jiangsu/1213/2019
A/swine/Beijing/0301/2018
A/swine/Henan/15/2019
A/swine/Henan/16/2019
A/swine/Jiangsu/1010/2019
A/swine/Henan/0315/2019
A/swine/Henan/0318/2019
A/swine/Shaanxi/1392/2016
A/swine/Guangxi/206/2016
A/swine/Hunan/269/2016
A/swine/Hunan/580/2017
A/swine/Hebei/1125/2019
A/swine/Hebei/20/2019
A/swine/Neimenggu/0326/2023
A/swine/Liaoning/267/2017
A/swine/Jiangsu/462/2017
A/swine/Guangxi/JL839/2021
A/swine/Liaoning/TL5404/2020
A/swine/Tianjin/117/2019
A/swine/Tianjin/182/2019
A/swine/Guangdong/132/2019
A/swine/Jilin/280/2017
A/swine/Hubei/580/2019
A/swine/Tianjin/18/2018
A/swine/Guangxi/NNJ3307/2021
A/Swine/Guangxi/NNYN6/2022
A/swine/Hebei/1231/2021
A/swine/Jiangsu/114/2023
A/swine/Shandong/105/2020
A/swine/Hebei/Y10/2021
A/swine/Hebei/11/2020
A/swine/Hebei/23/2020
A/swine/Shandong/0328/2022
A/swine/Chongqing/709/2017
A/swine/Henan/0212/2022
A/swine/Liaoning/321/2020
A/swine/Neimenggu/10/2020
A/swine/Shandong/1226A/2020
A/swine/Shandong/1224C/2020
A/swine/Shandong/S21/2020
A/swine/Shandong/1224D/2020
A/swine/Shandong/12/2020
A/swine/Shandong/115/2023
A/swine/Hunan/0302/2021
A/swine/Jiangsu/5354/2022
A/swine/Jiangsu/6162/2022
A/swine/Shandong/9192/2022
A/swine/Shandong/9596/2022
A/swine/Shandong/1378/2022
A/swine/Shandong/3536/2022
A/swine/Jiangsu/1278/2022
A/swine/Shandong/1067/2022
A/swine/Shandong/108/2023
A/swine/Shandong/3738/2022
A/swine/Shandong/2526/2022
A/swine/Shandong/1018/2022
A/swine/Shandong/3940/2022
0.03

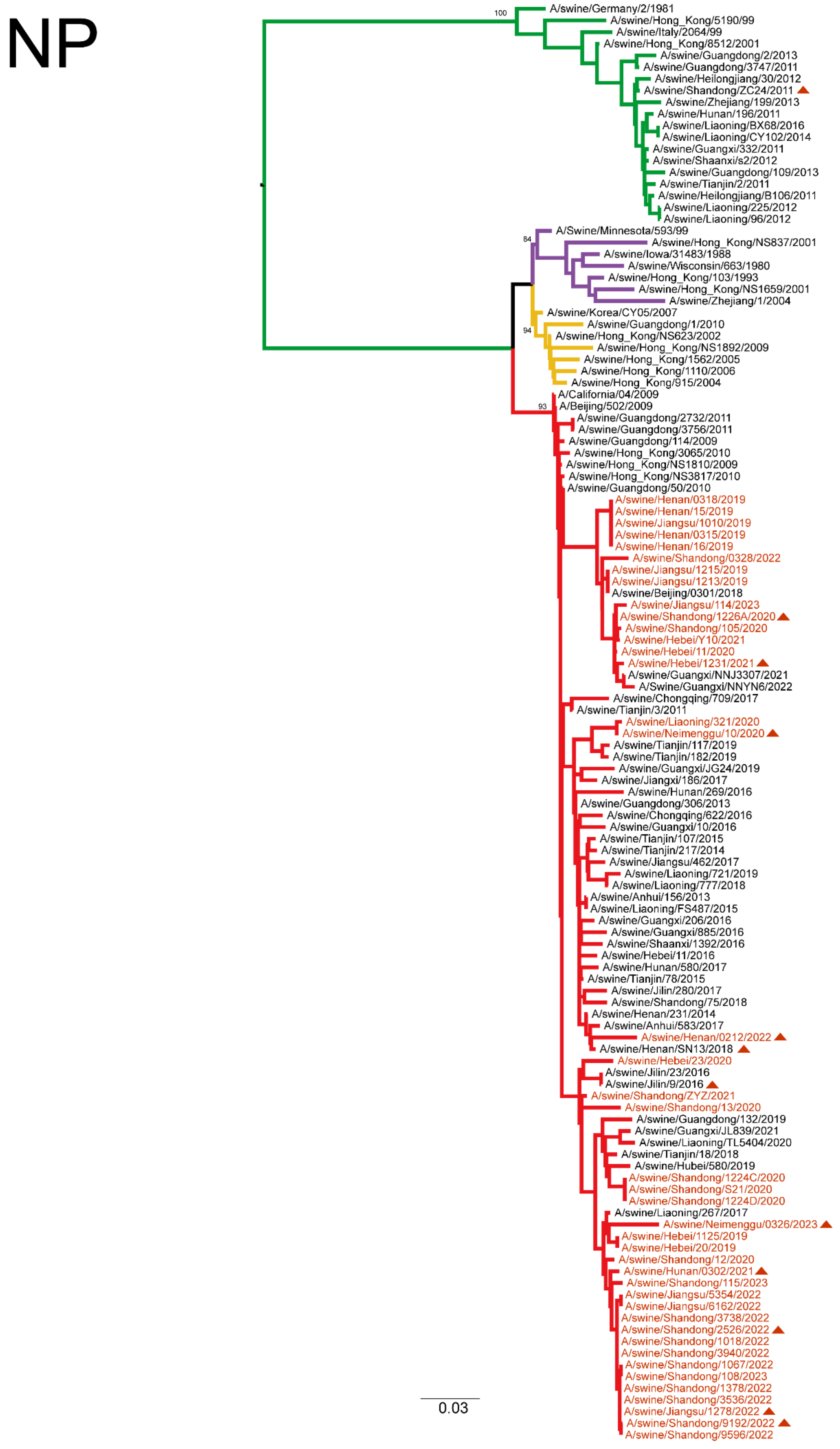
NP
100
A/swine/Germany/2/1981
A/swine/Hong_Kong/5190/99
A/swine/Italy/2064/99
A/swine/Hong_Kong/8512/2001
A/swine/Guangdong/2/2013
A/swine/Guangdong/3747/2011
A/swine/Heilongjiang/30/2012
A/swine/Shandong/ZC24/2011
A/swine/Zhejiang/199/2013
A/swine/Hunan/196/2011
A/swine/Liaoning/BX68/2016
A/swine/Liaoning/CY102/2014
A/swine/Guangxi/332/2011
A/swine/Shaanxi/s2/2012
A/swine/Guangdong/109/2013
A/swine/Tianjin/2/2011
A/swine/Heilongjiang/B106/2011
A/swine/Liaoning/225/2012
A/swine/Liaoning/96/2012
84
A/Swine/Minnesota/593/99
A/swine/Hong_Kong/NS837/2001
A/swine/Iowa/31483/1988
A/swine/Wisconsin/663/1980
A/swine/Hong_Kong/103/1993
A/swine/Hong_Kong/NS1659/2001
A/swine/Zhejiang/1/2004
A/swine/Korea/CY05/2007
94
A/swine/Guangdong/1/2010
A/swine/Hong_Kong/NS623/2002
A/swine/Hong_Kong/NS1892/2009
A/swine/Hong_Kong/1562/2005
A/swine/Hong_Kong/1110/2006
A/swine/Hong_Kong/915/2004
A/California/04/2009
93
A/Beijing/502/2009
A/swine/Guangdong/2732/2011
A/swine/Guangdong/3756/2011
A/swine/Guangdong/114/2009
A/swine/Hong_Kong/3065/2010
A/swine/Hong_Kong/NS1810/2009
A/swine/Hong_Kong/NS3817/2010
A/swine/Guangdong/50/2010
A/swine/Henan/0318/2019
A/swine/Henan/15/2019
A/swine/Jiangsu/1010/2019
A/swine/Henan/0315/2019
A/swine/Henan/16/2019
A/swine/Shandong/0328/2022
A/swine/Jiangsu/1215/2019
A/swine/Jiangsu/1213/2019
A/swine/Beijing/0301/2018
A/swine/Jiangsu/114/2023
A/swine/Shandong/1226A/2020
A/swine/Shandong/105/2020
A/swine/Hebei/Y10/2021
A/swine/Hebei/11/2020
A/swine/Hebei/1231/2021
A/swine/Guangxi/NNJ3307/2021
A/Swine/Guangxi/NNYN6/2022
A/swine/Chongqing/709/2017
A/swine/Tianjin/3/2011
A/swine/Liaoning/321/2020
A/swine/Neimenggu/10/2020
A/swine/Tianjin/117/2019
A/swine/Tianjin/182/2019
A/swine/Guangxi/JG24/2019
A/swine/Jiangxi/186/2017
A/swine/Hunan/269/2016
A/swine/Guangdong/306/2013
A/swine/Chongqing/622/2016
A/swine/Guangxi/10/2016
A/swine/Tianjin/107/2015
A/swine/Tianjin/217/2014
A/swine/Jiangsu/462/2017
A/swine/Liaoning/721/2019
A/swine/Liaoning/777/2018
A/swine/Anhui/156/2013
A/swine/Liaoning/FS487/2015
A/swine/Guangxi/206/2016
A/swine/Guangxi/885/2016
A/swine/Shaanxi/1392/2016
A/swine/Hebei/11/2016
A/swine/Hunan/580/2017
A/swine/Tianjin/78/2015
A/swine/Jilin/280/2017
A/swine/Shandong/75/2018
A/swine/Henan/231/2014
A/swine/Anhui/583/2017
A/swine/Henan/0212/2022
A/swine/Henan/SN13/2018
A/swine/Hebei/23/2020
A/swine/Jilin/23/2016
A/swine/Jilin/9/2016
A/swine/Shandong/ZYZ/2021
A/swine/Shandong/13/2020
A/swine/Guangdong/132/2019
A/swine/Guangxi/JL839/2021
A/swine/Liaoning/TL5404/2020
A/swine/Tianjin/18/2018
A/swine/Hubei/580/2019
A/swine/Shandong/1224C/2020
A/swine/Shandong/S21/2020
A/swine/Shandong/1224D/2020
A/swine/Liaoning/267/2017
A/swine/Neimenggu/0326/2023
A/swine/Hebei/1125/2019
A/swine/Hebei/20/2019
A/swine/Shandong/12/2020
A/swine/Hunan/0302/2021
A/swine/Shandong/115/2023
A/swine/Jiangsu/5354/2022
A/swine/Jiangsu/6162/2022
A/swine/Shandong/3738/2022
A/swine/Shandong/2526/2022
A/swine/Shandong/1018/2022
A/swine/Shandong/3940/2022
A/swine/Shandong/1067/2022
A/swine/Shandong/108/2023
A/swine/Shandong/1378/2022
A/swine/Shandong/3536/2022
A/swine/Jiangsu/1278/2022
A/swine/Shandong/9192/2022
A/swine/Shandong/9596/2022
0.03

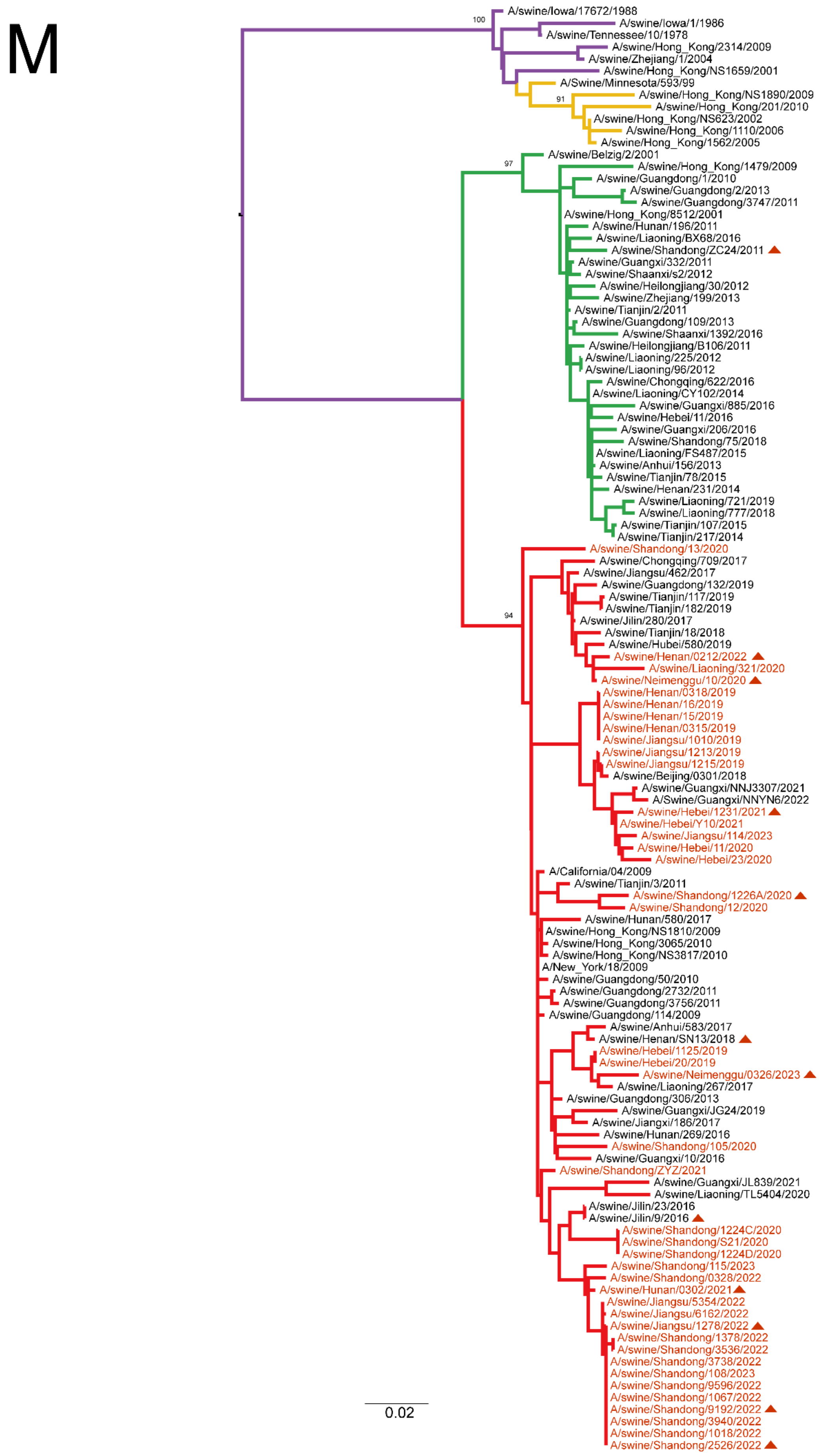
M
100
A/swine/Iowa/17672/1988
A/swine/Iowa/1/1986
A/swine/Tennessee/10/1978
A/swine/Hong_Kong/2314/2009
A/swine/Zhejiang/1/2004
A/swine/Hong_Kong/NS1659/2001
A/Swine/Minnesota/593/99
91
A/swine/Hong_Kong/NS1890/2009
A/swine/Hong_Kong/201/2010
A/swine/Hong_Kong/NS623/2002
A/swine/Hong_Kong/1110/2006
A/swine/Hong_Kong/1562/2005
97
A/swine/Belzig/2/2001
A/swine/Hong_Kong/1479/2009
A/swine/Guangdong/1/2010
A/swine/Guangdong/2/2013
A/swine/Guangdong/3747/2011
A/swine/Hong_Kong/8512/2001
A/swine/Hunan/196/2011
A/swine/Liaoning/BX68/2016
A/swine/Shandong/ZC24/2011
A/swine/Guangxi/332/2011
A/swine/Shaanxi/s2/2012
A/swine/Heilongjiang/30/2012
A/swine/Zhejiang/199/2013
A/swine/Tianjin/2/2011
A/swine/Guangdong/109/2013
A/swine/Shaanxi/1392/2016
A/swine/Heilongjiang/B106/2011
A/swine/Liaoning/225/2012
A/swine/Liaoning/96/2012
A/swine/Chongqing/622/2016
A/swine/Liaoning/CY102/2014
A/swine/Guangxi/885/2016
A/swine/Hebei/11/2016
A/swine/Guangxi/206/2016
A/swine/Shandong/75/2018
A/swine/Liaoning/FS487/2015
A/swine/Anhui/156/2013
A/swine/Tianjin/78/2015
A/swine/Henan/231/2014
A/swine/Liaoning/721/2019
A/swine/Liaoning/777/2018
A/swine/Tianjin/107/2015
A/swine/Tianjin/217/2014
A/swine/Shandong/13/2020
A/swine/Chongqing/709/2017
A/swine/Jiangsu/462/2017
A/swine/Guangdong/132/2019
A/swine/Tianjin/117/2019
A/swine/Tianjin/182/2019
94
A/swine/Jilin/280/2017
A/swine/Tianjin/18/2018
A/swine/Hubei/580/2019
A/swine/Henan/0212/2022
A/swine/Liaoning/321/2020
A/swine/Neimenggu/10/2020
A/swine/Henan/0318/2019
A/swine/Henan/16/2019
A/swine/Henan/15/2019
A/swine/Henan/0315/2019
A/swine/Jiangsu/1010/2019
A/swine/Jiangsu/1213/2019
A/swine/Jiangsu/1215/2019
A/swine/Beijing/0301/2018
A/swine/Guangxi/NNJ3307/2021
A/Swine/Guangxi/NNYN6/2022
A/swine/Hebei/1231/2021
A/swine/Hebei/Y10/2021
A/swine/Jiangsu/114/2023
A/swine/Hebei/11/2020
A/swine/Hebei/23/2020
A/California/04/2009
A/swine/Tianjin/3/2011
A/swine/Shandong/1226A/2020
A/swine/Shandong/12/2020
A/swine/Hunan/580/2017
A/swine/Hong_Kong/NS1810/2009
A/swine/Hong_Kong/3065/2010
A/swine/Hong_Kong/NS3817/2010
A/New_York/18/2009
A/swine/Guangdong/50/2010
A/swine/Guangdong/2732/2011
A/swine/Guangdong/3756/2011
A/swine/Guangdong/114/2009
A/swine/Anhui/583/2017
A/swine/Henan/SN13/2018
A/swine/Hebei/1125/2019
A/swine/Hebei/20/2019
A/swine/Neimenggu/0326/2023
A/swine/Liaoning/267/2017
A/swine/Guangdong/306/2013
A/swine/Guangxi/JG24/2019
A/swine/Jiangxi/186/2017
A/swine/Hunan/269/2016
A/swine/Shandong/105/2020
A/swine/Guangxi/10/2016
A/swine/Shandong/ZYZ/2021
A/swine/Guangxi/JL839/2021
A/swine/Liaoning/TL5404/2020
A/swine/Jilin/23/2016
A/swine/Jilin/9/2016
A/swine/Shandong/1224C/2020
A/swine/Shandong/S21/2020
A/swine/Shandong/1224D/2020
A/swine/Shandong/115/2023
A/swine/Shandong/0328/2022
A/swine/Hunan/0302/2021
A/swine/Jiangsu/5354/2022
A/swine/Jiangsu/6162/2022
A/swine/Jiangsu/1278/2022
A/swine/Shandong/1378/2022
A/swine/Shandong/3536/2022
A/swine/Shandong/3738/2022
A/swine/Shandong/108/2023
A/swine/Shandong/9596/2022
A/swine/Shandong/1067/2022
A/swine/Shandong/9192/2022
A/swine/Shandong/3940/2022
A/swine/Shandong/1018/2022
A/swine/Shandong/2526/2022
0.02

NS

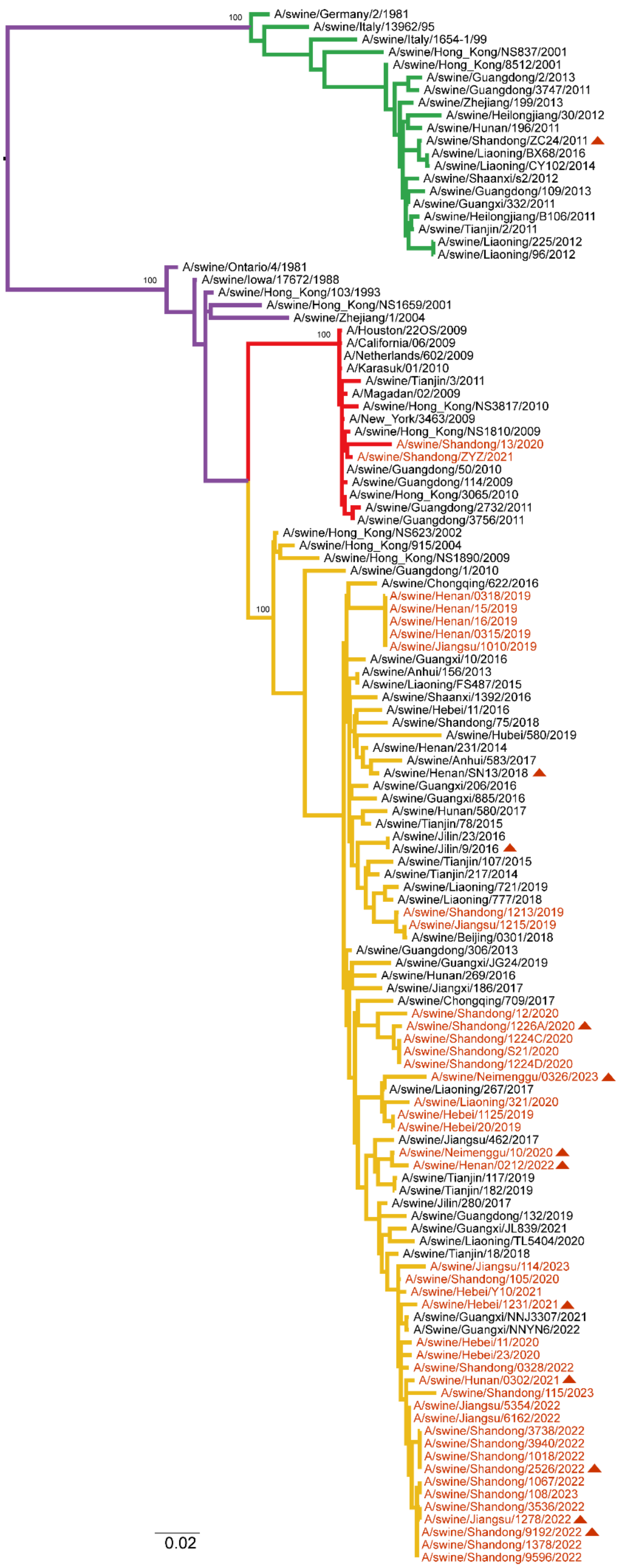
100
A/swine/Germany/2/1981
A/swine/Italy/13962/95
A/swine/Italy/1654-1/99
A/swine/Hong_Kong/NS837/2001
A/swine/Hong_Kong/8512/2001
A/swine/Guangdong/2/2013
A/swine/Guangdong/3747/2011
A/swine/Zhejiang/199/2013
A/swine/Heilongjiang/30/2012
A/swine/Hunan/196/2011
A/swine/Shandong/ZC24/2011
A/swine/Liaoning/BX68/2016
A/swine/Liaoning/CY102/2014
A/swine/Shaanxi/s2/2012
A/swine/Guangdong/109/2013
A/swine/Guangxi/332/2011
A/swine/Heilongjiang/B106/2011
A/swine/Tianjin/2/2011
A/swine/Liaoning/225/2012
A/swine/Liaoning/96/2012
A/swine/Ontario/4/1981
100
A/swine/Iowa/17672/1988
A/swine/Hong_Kong/103/1993
A/swine/Hong_Kong/NS1659/2001
A/swine/Zhejiang/1/2004
100
A/Houston/22OS/2009
A/California/06/2009
A/Netherlands/602/2009
A/Karasuk/01/2010
A/swine/Tianjin/3/2011
A/Magadan/02/2009
A/swine/Hong_Kong/NS3817/2010
A/New_York/3463/2009
A/swine/Hong_Kong/NS1810/2009
A/swine/Shandong/13/2020
A/swine/Shandong/ZYZ/2021
A/swine/Guangdong/50/2010
A/swine/Guangdong/114/2009
A/swine/Hong_Kong/3065/2010
A/swine/Guangdong/2732/2011
A/swine/Guangdong/3756/2011
A/swine/Hong_Kong/NS623/2002
A/swine/Hong_Kong/915/2004
A/swine/Hong_Kong/NS1890/2009
A/swine/Guangdong/1/2010
A/swine/Chongqing/622/2016
100
A/swine/Henan/0318/2019
A/swine/Henan/15/2019
A/swine/Henan/16/2019
A/swine/Henan/0315/2019
A/swine/Jiangsu/1010/2019
A/swine/Guangxi/10/2016
A/swine/Anhui/156/2013
A/swine/Liaoning/FS487/2015
A/swine/Shaanxi/1392/2016
A/swine/Hebei/11/2016
A/swine/Shandong/75/2018
A/swine/Hubei/580/2019
A/swine/Henan/231/2014
A/swine/Anhui/583/2017
A/swine/Henan/SN13/2018
A/swine/Guangxi/206/2016
A/swine/Guangxi/885/2016
A/swine/Hunan/580/2017
A/swine/Tianjin/78/2015
A/swine/Jilin/23/2016
A/swine/Jilin/9/2016
A/swine/Tianjin/107/2015
A/swine/Tianjin/217/2014
A/swine/Liaoning/721/2019
A/swine/Liaoning/777/2018
A/swine/Shandong/1213/2019
A/swine/Jiangsu/1215/2019
A/swine/Beijing/0301/2018
A/swine/Guangdong/306/2013
A/swine/Guangxi/JG24/2019
A/swine/Hunan/269/2016
A/swine/Jiangxi/186/2017
A/swine/Chongqing/709/2017
A/swine/Shandong/12/2020
A/swine/Shandong/1226A/2020
A/swine/Shandong/1224C/2020
A/swine/Shandong/S21/2020
A/swine/Shandong/1224D/2020
A/swine/Neimenggu/0326/2023
A/swine/Liaoning/267/2017
A/swine/Liaoning/321/2020
A/swine/Hebei/1125/2019
A/swine/Hebei/20/2019
A/swine/Jiangsu/462/2017
A/swine/Neimenggu/10/2020
A/swine/Henan/0212/2022
A/swine/Tianjin/117/2019
A/swine/Tianjin/182/2019
A/swine/Jilin/280/2017
A/swine/Guangdong/132/2019
A/swine/Guangxi/JL839/2021
A/swine/Liaoning/TL5404/2020
A/swine/Tianjin/18/2018
A/swine/Jiangsu/114/2023
A/swine/Shandong/105/2020
A/swine/Hebei/Y10/2021
A/swine/Hebei/1231/2021
A/swine/Guangxi/NNJ3307/2021
A/Swine/Guangxi/NNYN6/2022
A/swine/Hebei/11/2020
A/swine/Hebei/23/2020
A/swine/Shandong/0328/2022
A/swine/Hunan/0302/2021
A/swine/Shandong/115/2023
A/swine/Jiangsu/5354/2022
A/swine/Jiangsu/6162/2022
A/swine/Shandong/3738/2022
A/swine/Shandong/3940/2022
A/swine/Shandong/1018/2022
A/swine/Shandong/2526/2022
A/swine/Shandong/1067/2022
A/swine/Shandong/108/2023
A/swine/Shandong/3536/2022
A/swine/Jiangsu/1278/2022
A/swine/Shandong/9192/2022
A/swine/Shandong/1378/2022
A/swine/Shandong/9596/2022
0.02

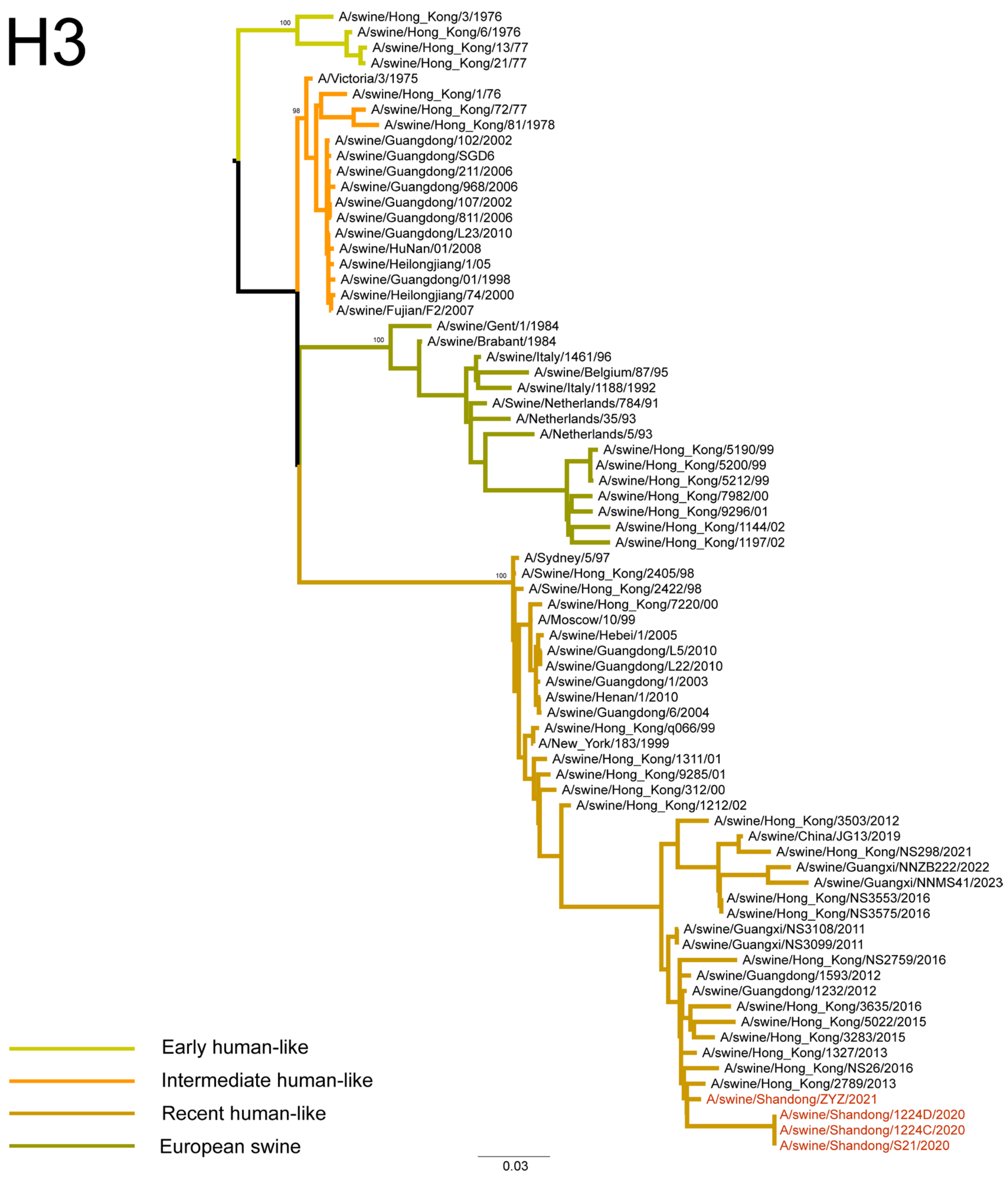
H3
100
A/swine/Hong_Kong/3/1976
A/swine/Hong_Kong/6/1976
A/swine/Hong_Kong/13/77
A/swine/Hong_Kong/21/77
A/Victoria/3/1975
98
A/swine/Hong_Kong/1/76
A/swine/Hong_Kong/72/77
A/swine/Hong_Kong/81/1978
A/swine/Guangdong/102/2002
A/swine/Guangdong/SGD6
A/swine/Guangdong/211/2006
A/swine/Guangdong/968/2006
A/swine/Guangdong/107/2002
A/swine/Guangdong/811/2006
A/swine/Guangdong/L23/2010
A/swine/HuNan/01/2008
A/swine/Heilongjiang/1/05
A/swine/Guangdong/01/1998
A/swine/Heilongjiang/74/2000
A/swine/Fujian/F2/2007
A/swine/Gent/1/1984
100
A/swine/Brabant/1984
A/swine/Italy/1461/96
A/swine/Belgium/87/95
A/swine/Italy/1188/1992
A/Swine/Netherlands/784/91
A/Netherlands/35/93
A/Netherlands/5/93
A/swine/Hong_Kong/5190/99
A/swine/Hong_Kong/5200/99
A/swine/Hong_Kong/5212/99
A/swine/Hong_Kong/7982/00
A/swine/Hong_Kong/9296/01
A/swine/Hong_Kong/1144/02
A/swine/Hong_Kong/1197/02
A/Sydney/5/97
100
A/Swine/Hong_Kong/2405/98
A/Swine/Hong_Kong/2422/98
A/swine/Hong_Kong/7220/00
A/Moscow/10/99
A/swine/Hebei/1/2005
A/swine/Guangdong/L5/2010
A/swine/Guangdong/L22/2010
A/swine/Guangdong/1/2003
A/swine/Henan/1/2010
A/swine/Guangdong/6/2004
A/swine/Hong_Kong/q066/99
A/New_York/183/1999
A/swine/Hong_Kong/1311/01
A/swine/Hong_Kong/9285/01
A/swine/Hong_Kong/312/00
A/swine/Hong_Kong/1212/02
A/swine/Hong_Kong/3503/2012
A/swine/China/JG13/2019
A/swine/Hong_Kong/NS298/2021
A/swine/Guangxi/NNZB222/2022
A/swine/Guangxi/NNMS41/2023
A/swine/Hong_Kong/NS3553/2016
A/swine/Hong_Kong/NS3575/2016
A/swine/Guangxi/NS3108/2011
A/swine/Guangxi/NS3099/2011
A/swine/Hong_Kong/NS2759/2016
A/swine/Guangdong/1593/2012
A/swine/Guangdong/1232/2012
A/swine/Hong_Kong/3635/2016
A/swine/Hong_Kong/5022/2015
A/swine/Hong_Kong/3283/2015
A/swine/Hong_Kong/1327/2013
A/swine/Hong_Kong/NS26/2016
A/swine/Hong_Kong/2789/2013
A/swine/Shandong/ZYZ/2021
A/swine/Shandong/1224D/2020
A/swine/Shandong/1224C/2020
A/swine/Shandong/S21/2020
Early human-like
Intermediate human-like
Recent human-like
European swine
0.03

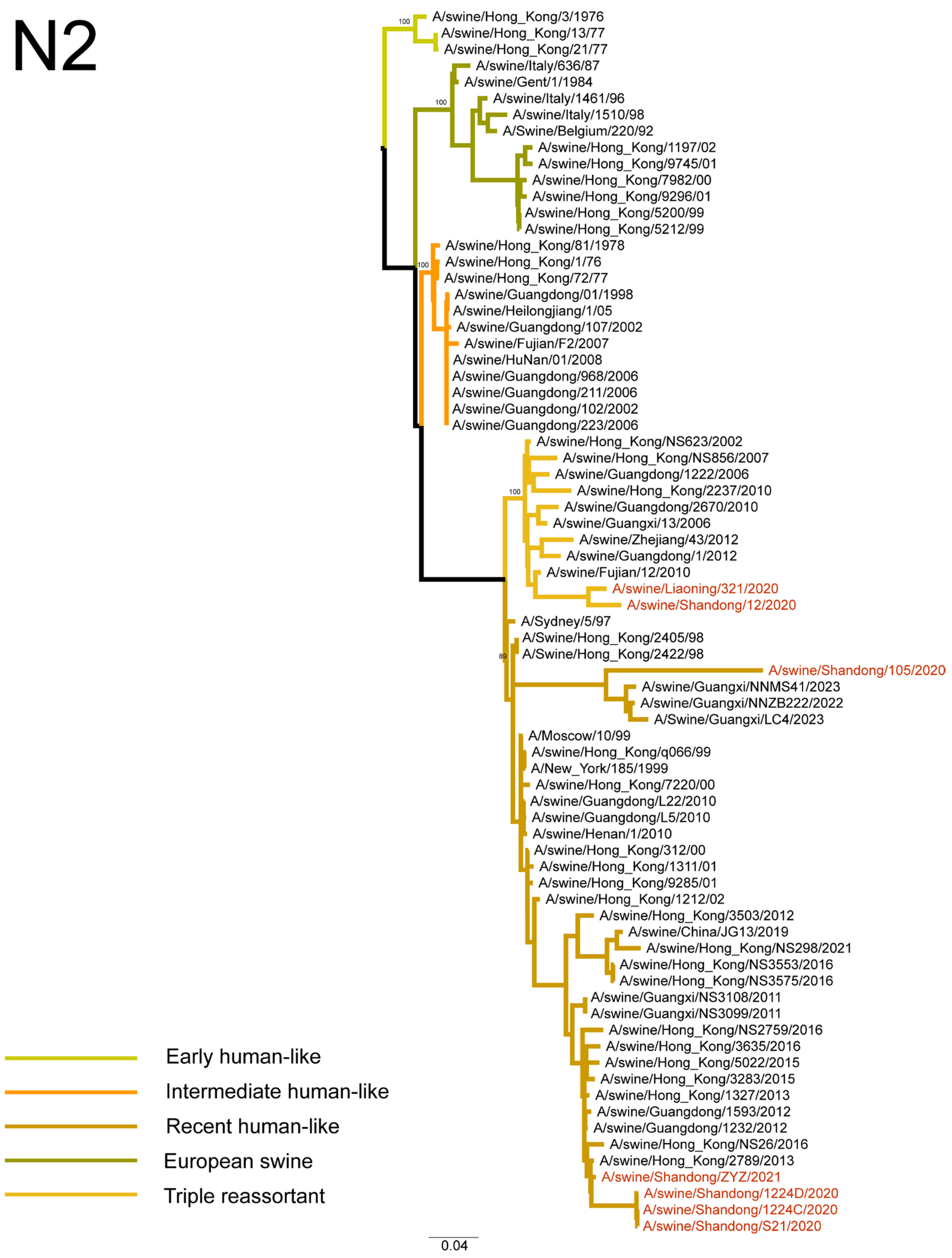

Figure.S1. Phylogenetic analysis of the genes of EA viruses or H3N2 viruses isolated in this study. Phylogenetic trees were estimated using genetic distances calculated by maximum likelihood under the GTRGAMMA + I model. The sequences of viruses with names listed in black were downloaded from the GISAID or NCBI database. Names of SIVs isolated in this study was marked with red and the strains selected for further evaluation of viral characteristics were marked with triangles. Node labels represent bootstrap values. Scale bar indicates estimated genetic distance.

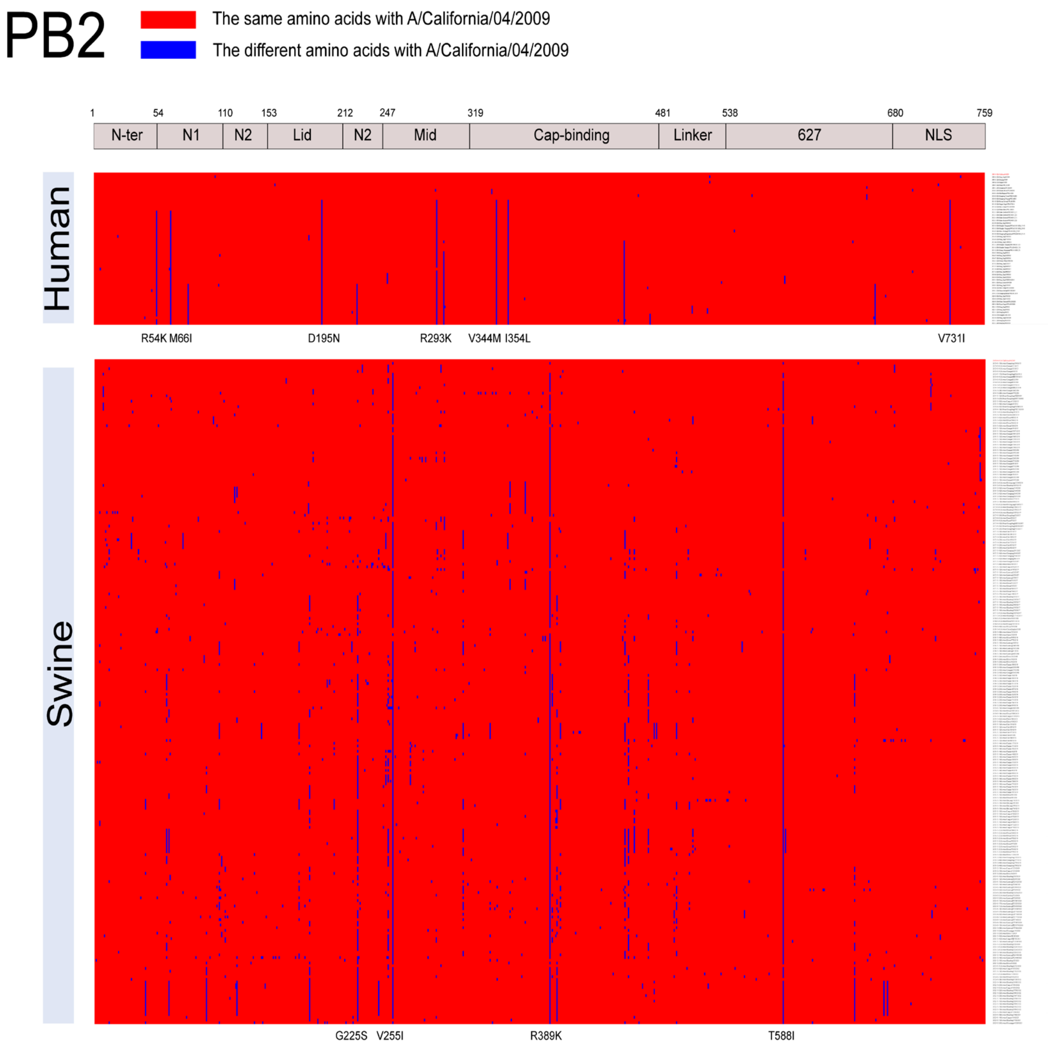

PB2
The same amino acids with A/California/04/2009
The different amino acids with A/California/04/2009
1
54
110
153
212
247
319
481
538
680
759
N-ter
N1
N2
Lid
N2
Mid
Cap-binding
Linker
627
NLS
Human
R54K M66I
D195N
R293K
V344M I354L
V731I
Swine
G225S
V255I
R389K
T588I

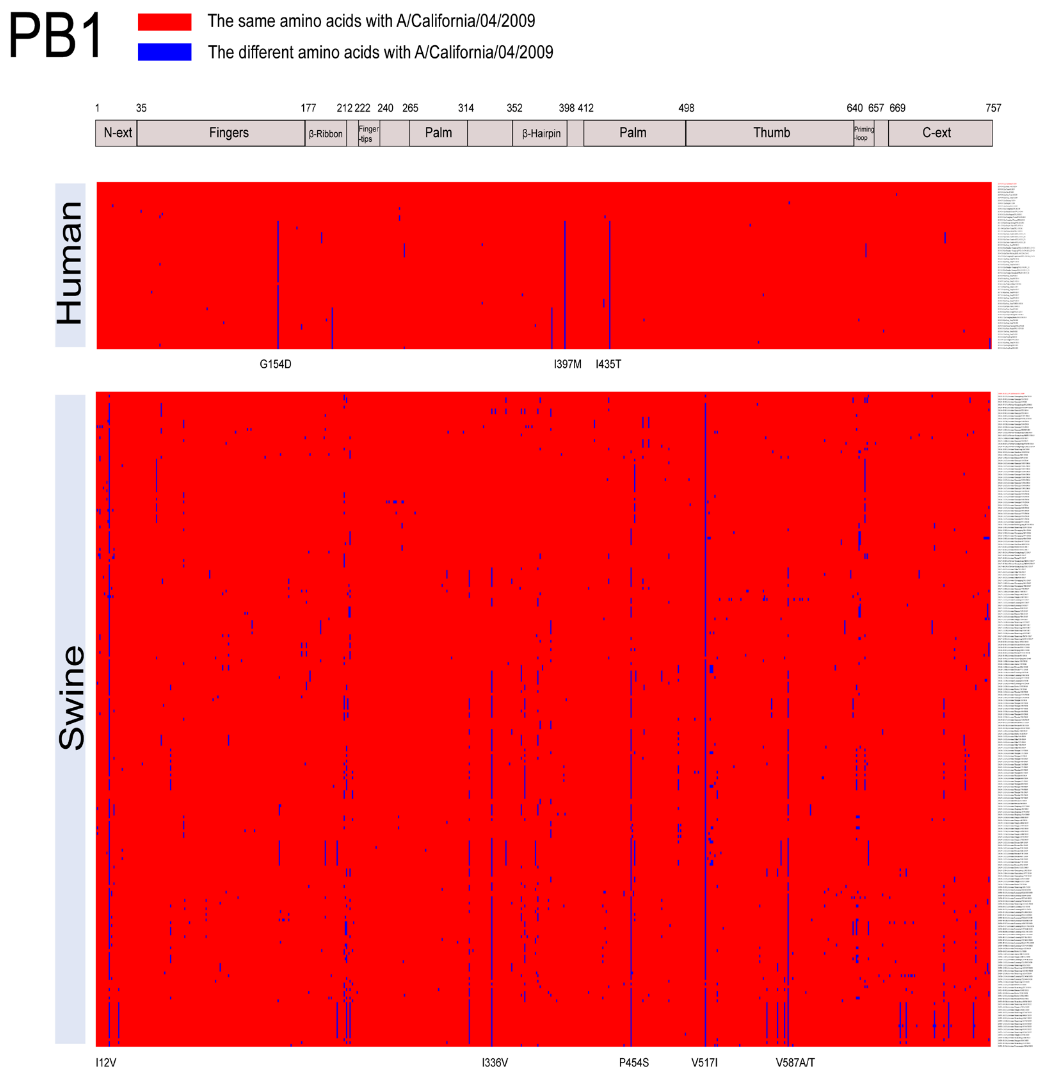
PB1
The same amino acids with A/California/04/2009
The different amino acids with A/California/04/2009
1
35
177
212
222
240
265
314
352
398
412
498
640
657
669
757
N-ext
Fingers
β-Ribbon
Finger-tips
Palm
β-Hairpin
Palm
Thumb
Priming-loop
C-ext
Human
G154D
I397M
I435T
Swine
I12V
I336V
P454S
V517I
V587A/T

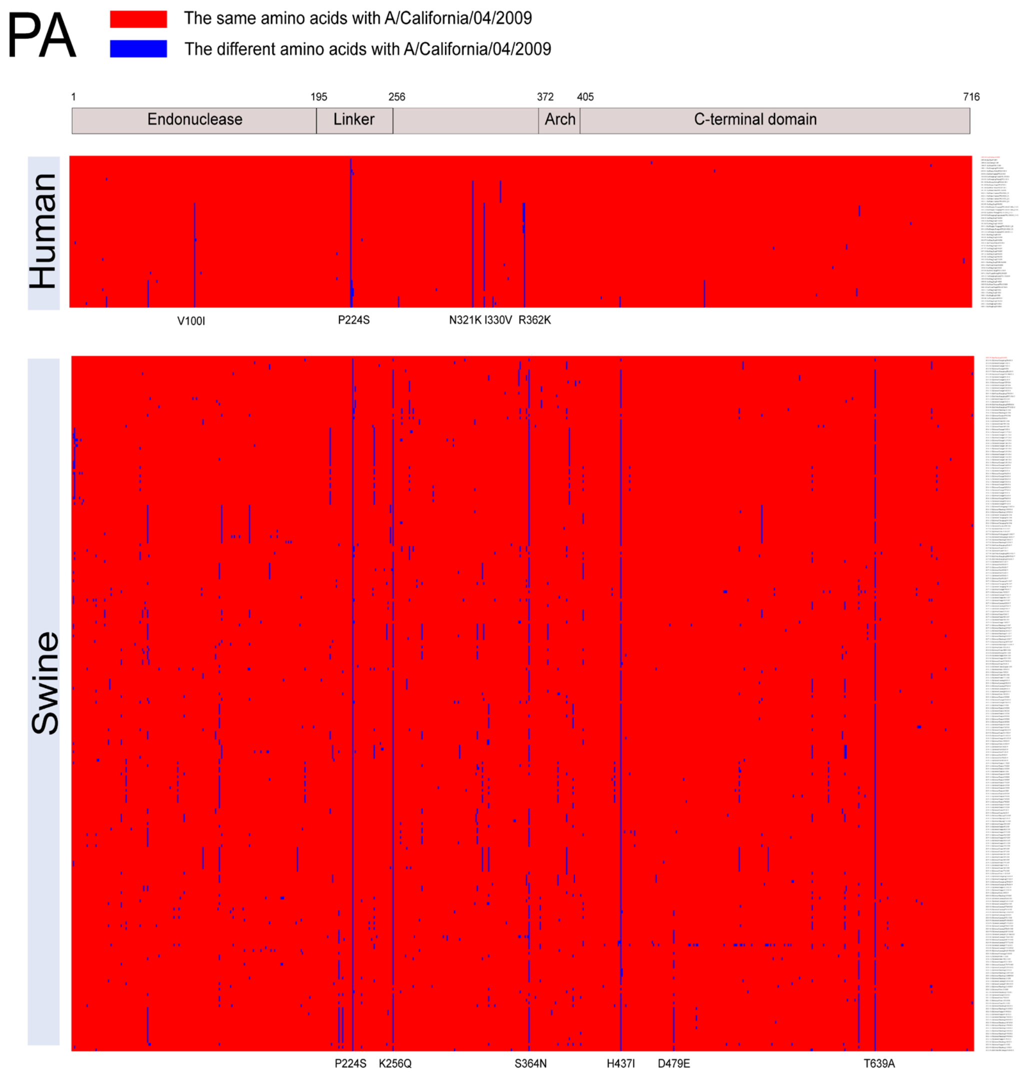
PA
The same amino acids with A/California/04/2009
The different amino acids with A/California/04/2009
1
195
256
372
405
716
Endonuclease
Linker
Arch
C-terminal domain
Human
V100I
P224S
N321K I330V
R362K
Swine
P224S
K256Q
S364N
H437I
D479E
T639A

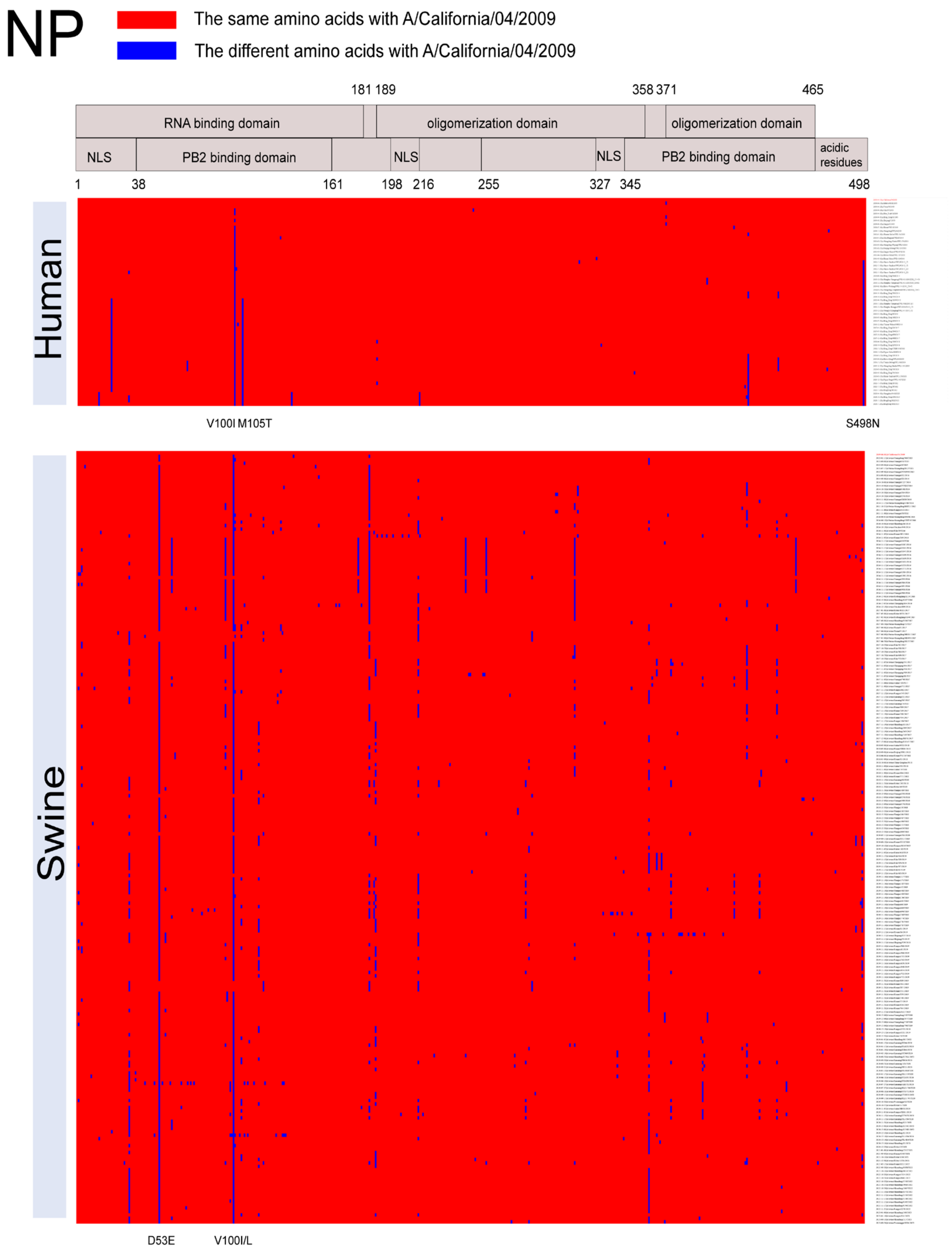

NP
The same amino acids with A/California/04/2009
The different amino acids with A/California/04/2009
181 189
358 371
465
RNA binding domain
oligomerization domain
oligomerization domain
NLS
PB2 binding domain
NLS
NLS
PB2 binding domain
acidic residues
1
38
161
198
216
255
327
345
498
Human
V100I M105T
S498N
Swine
D53E
V100I/L

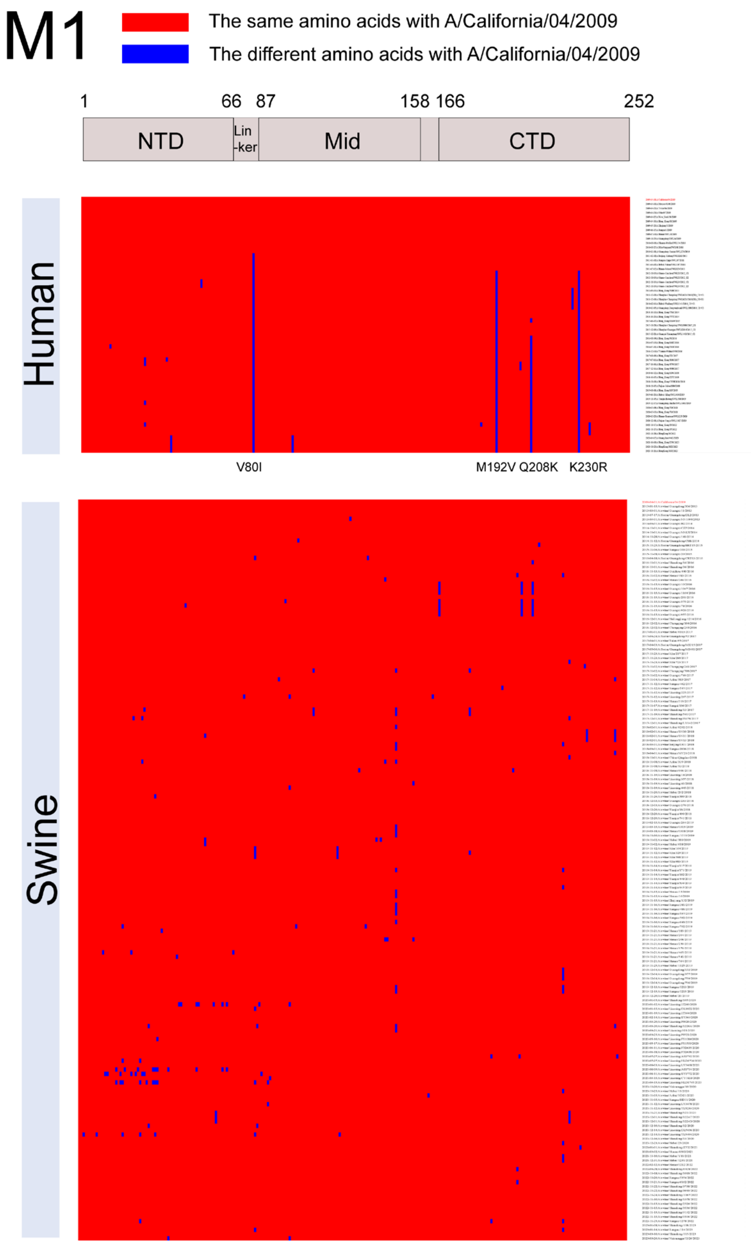
M1
The same amino acids with A/California/04/2009
The different amino acids with A/California/04/2009
1
66
87
158
166
252
NTD
Lin
-ker
Mid
CTD
Human
V80I
M192V Q208K
K230R
Swine

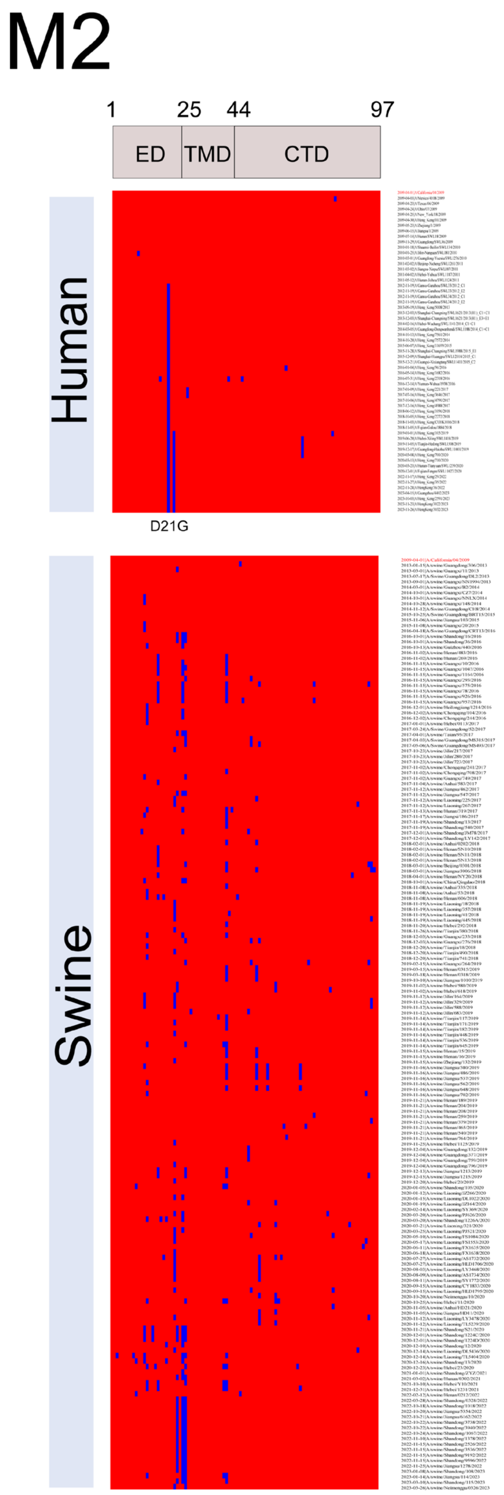

Figure S2. Heat maps of amino acid variations in the PB2, PB1, PA, NP, and M genes of G4 EA H1N1 and human pdm/09 H1N1 viruses. The red color in the heat map represents the same amino acid mutation as the reference virus (A/California/04/2009) at the corresponding site, and the blue color represents the amino acid mutation that is different from the ancestor virus.
