# Supplementary material for: Continuous evolution of Eurasian avian-like H1N1 swine influenza viruses with pdm/09-derived internal genes enhances pathogenicity in mice
Source: J Virol. 2025 Sep 8;99(10):e00430-25. doi: 10.1128/jvi.00430-25 (PMC12548388; doi:10.1128/jvi.00430-25)
Supplement: Fig. S3 — Characteristics of reassortant Eurasian avian-like H1N1 virus. [file jvi.00430-25-s0002.pdf]

Supplementary Materials

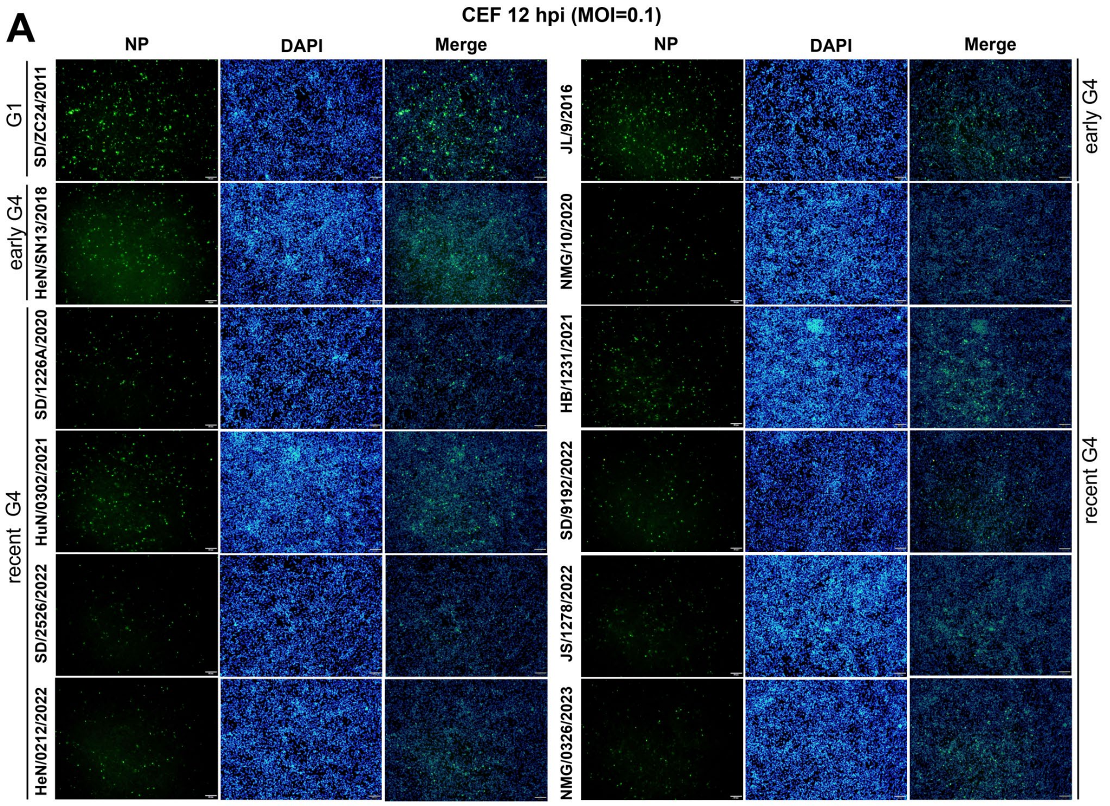

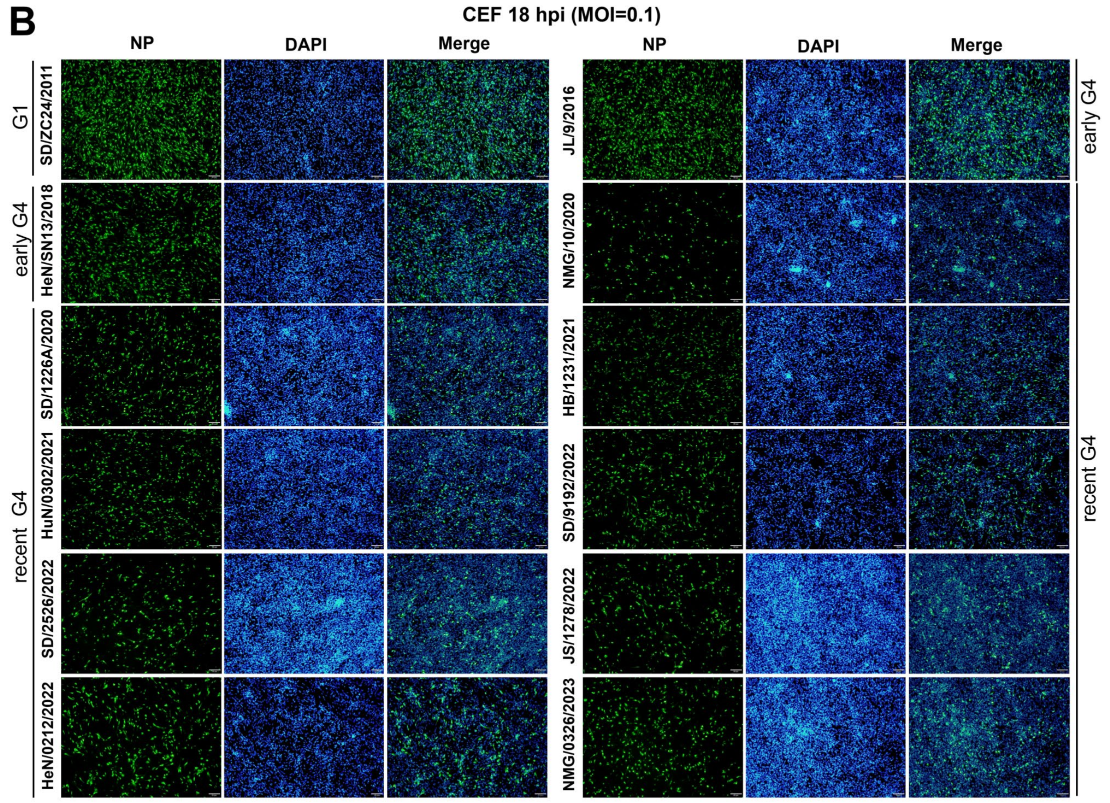

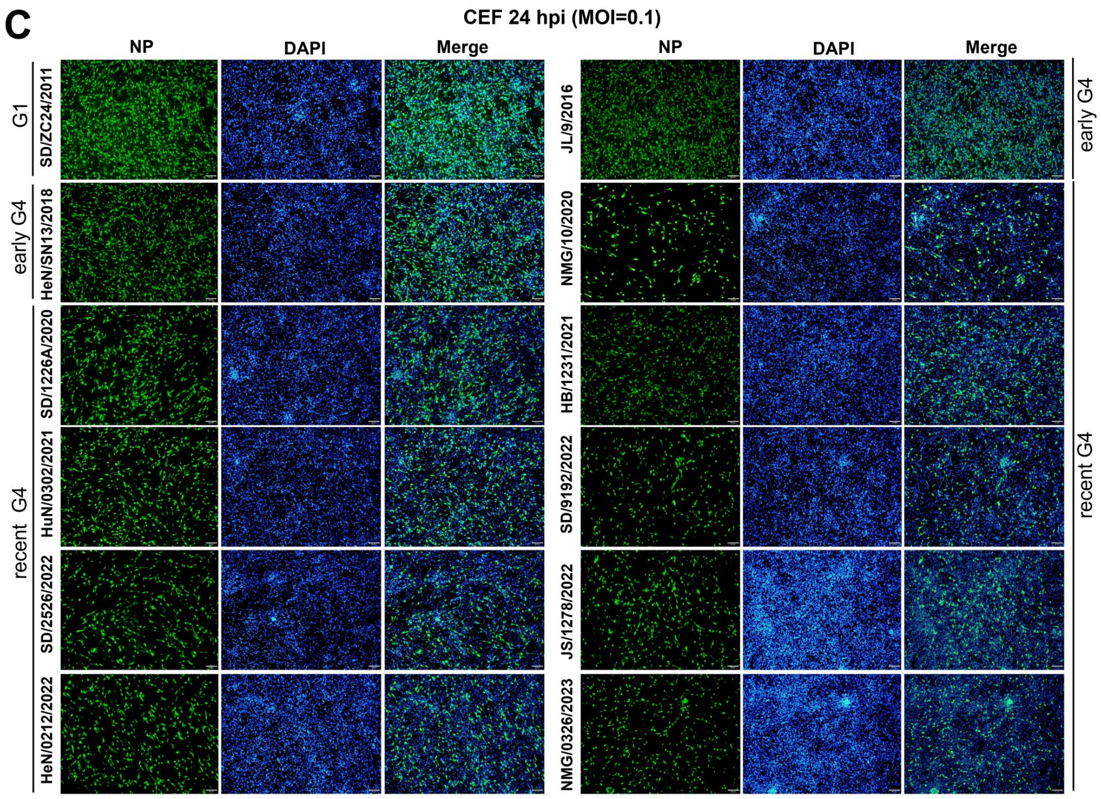
C
CEF 24 hpi (MOI=0.1)
NP
DAPI
Merge
G1
SD/ZC24/2011
early G4
HeN/SN13/2018
recent G4
SD/1226A/2020
HuN/0302/2021
SD/2526/2022
HeN/0212/2022
JL/9/2016
NMG/10/2020
HB/1231/2021
SD/9192/2022
JS/1278/2022
NMG/0326/2023

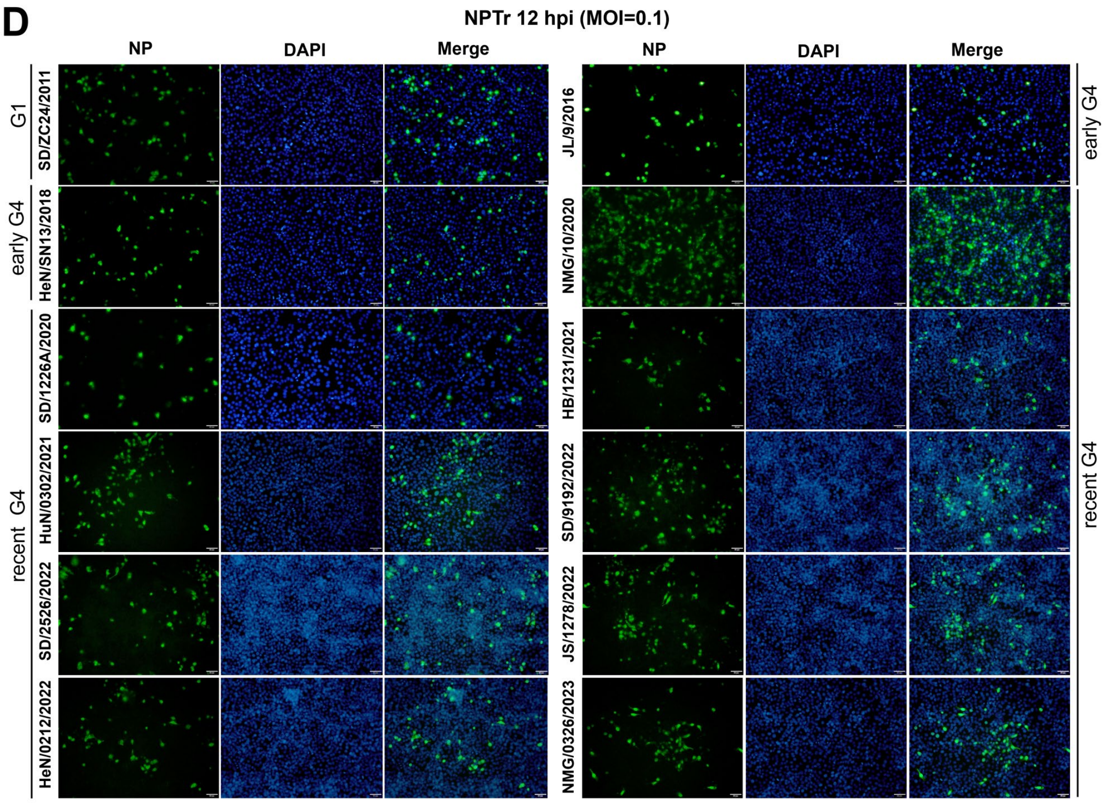
D
NPTr 12 hpi (MOI=0.1)
NP
DAPI
Merge
G1
SD/ZC24/2011
early G4
HeN/SN13/2018
recent G4
SD/1226A/2020
HuN/0302/2021
SD/2526/2022
HeN/0212/2022
JL/9/2016
NMG/10/2020
HB/1231/2021
SD/9192/2022
JS/1278/2022
NMG/0326/2023

E

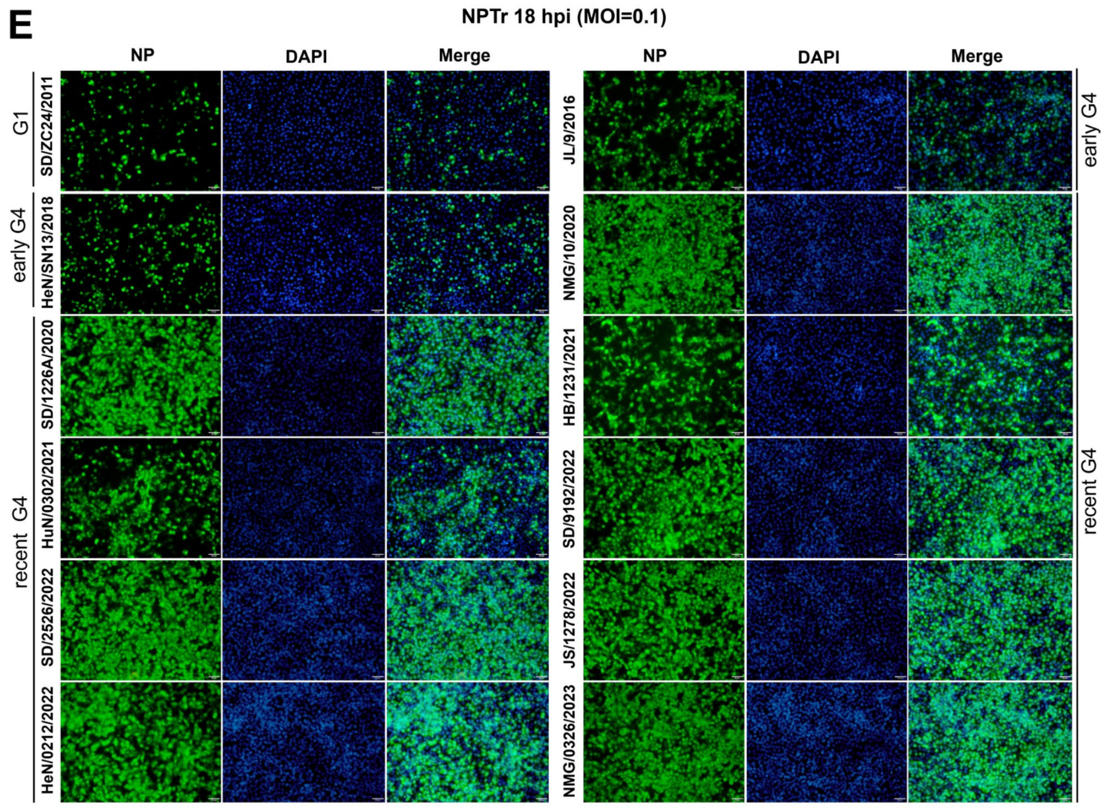
NPTr 18 hpi (MOI=0.1)
NP
DAPI
Merge
NP
DAPI
Merge
G1
SD/ZC24/2011
early G4
HeN/SN13/2018
recent G4
SD/1226A/2020
HuN/0302/2021
SD/2526/2022
HeN/0212/2022
JL/9/2016
early G4
NMG/10/2020
HB/1231/2021
SD/9192/2022
JS/1278/2022
NMG/0326/2023
recent G4

F

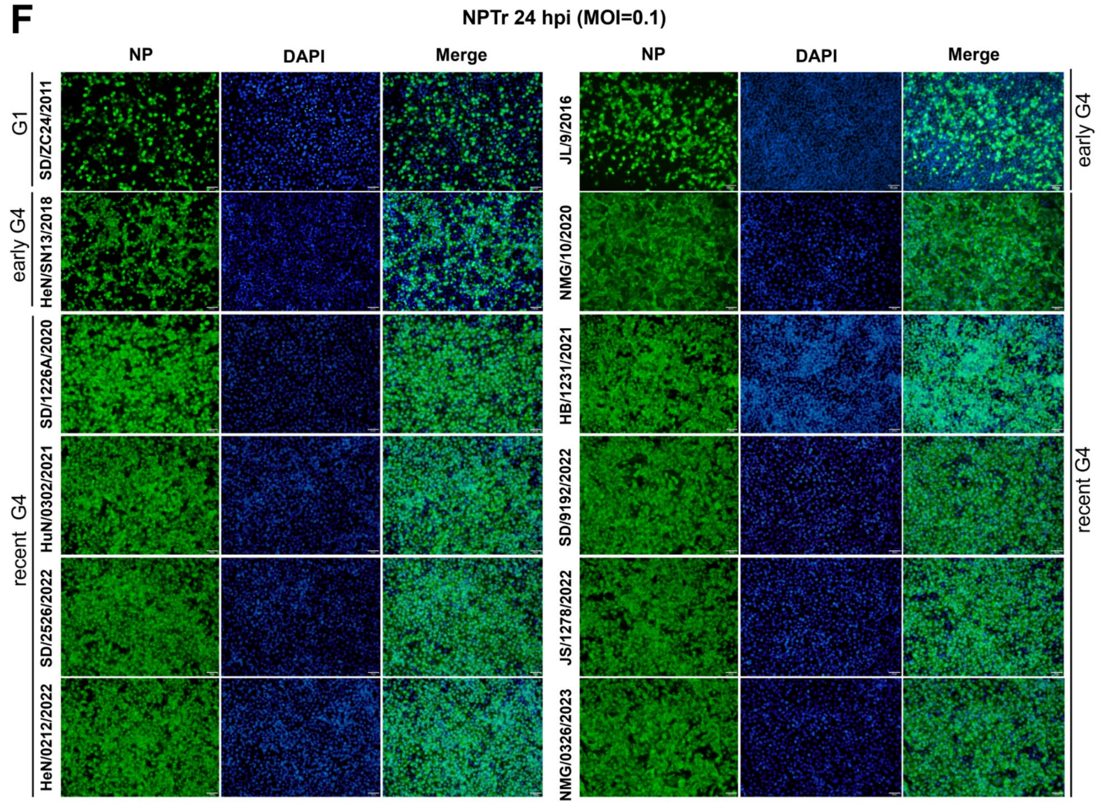
NPTr 24 hpi (MOI=0.1)
NP
DAPI
Merge
NP
DAPI
Merge
G1
SD/ZC24/2011
early G4
HeN/SN13/2018
recent G4
SD/1226A/2020
HuN/0302/2021
SD/2526/2022
HeN/0212/2022
JL/9/2016
early G4
NMG/10/2020
HB/1231/2021
SD/9192/2022
JS/1278/2022
NMG/0326/2023
recent G4

G

A549 12 hpi (MOI=0.1)

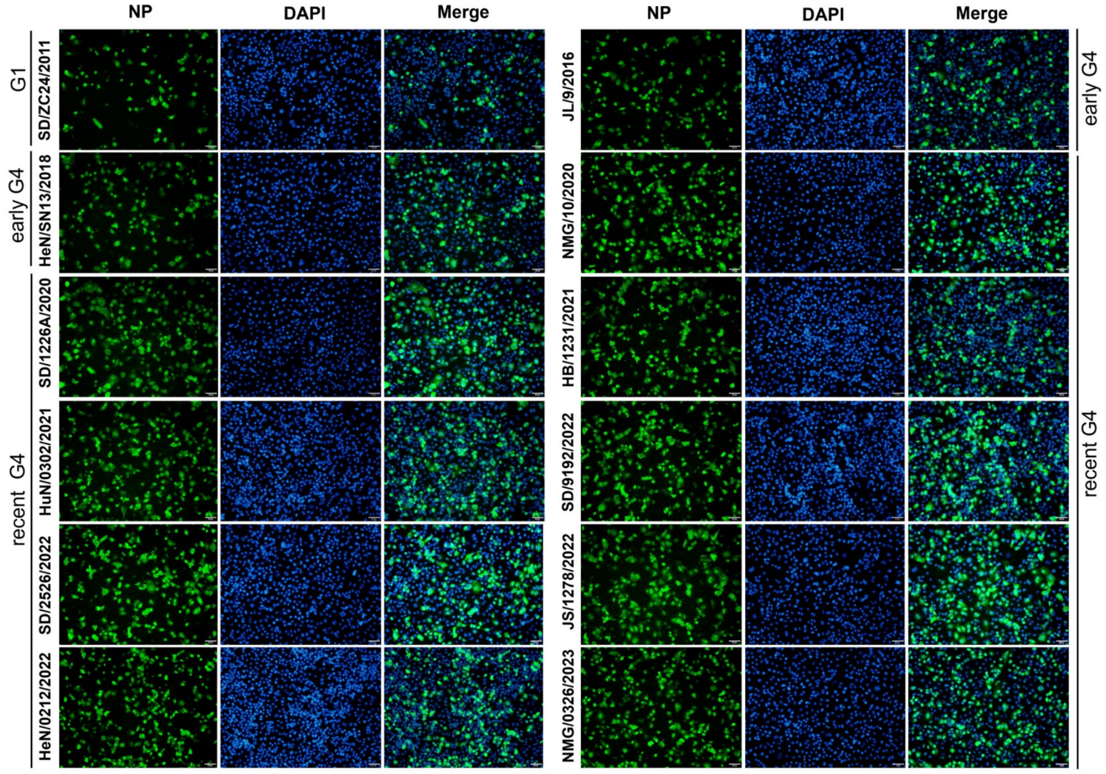

H

A549 18 hpi (MOI=0.1)

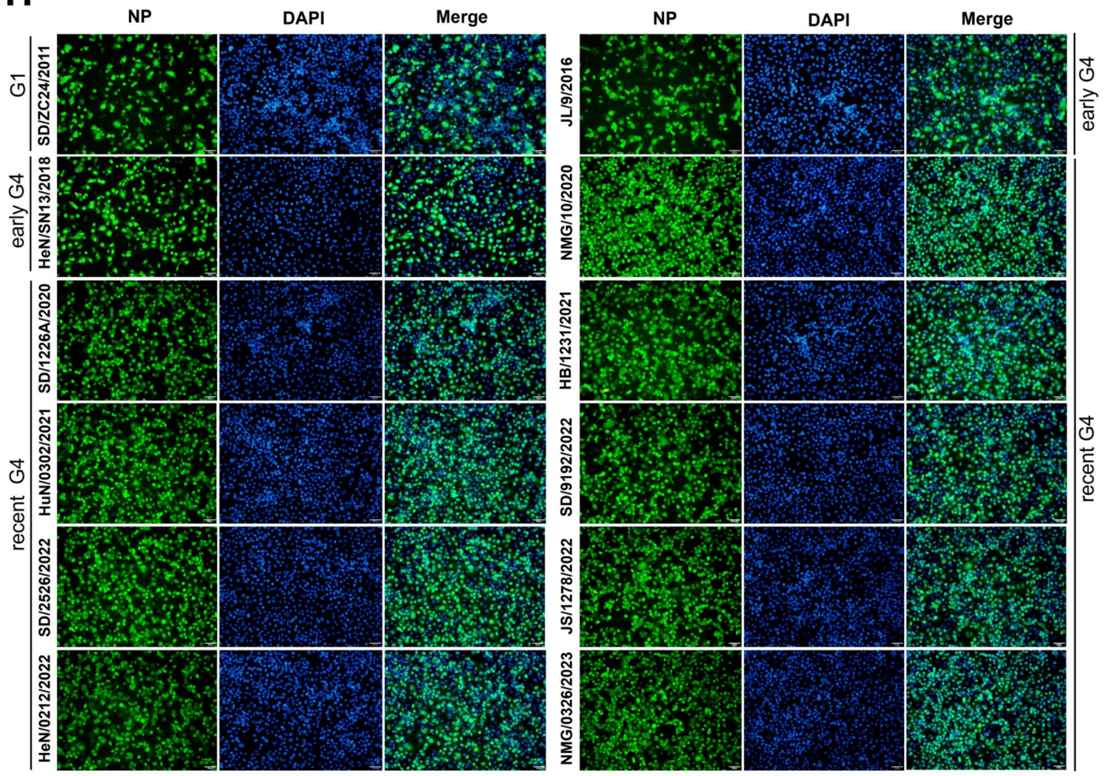

I

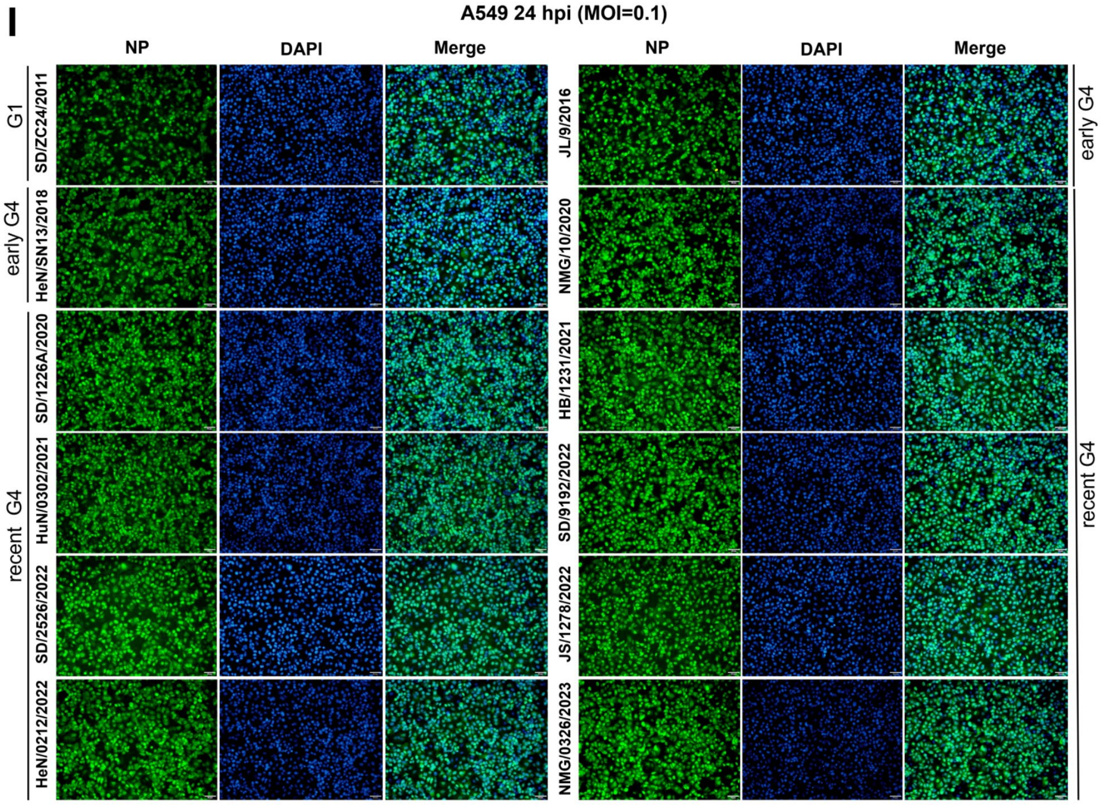
A549 24 hpi (MOI=0.1)
NP
DAPI
Merge
G1
early G4
recent G4
SD/ZC24/2011
HeN/SN13/2018
SD/1226A/2020
HuN/0302/2021
SD/2526/2022
HeN/0212/2022
JL/9/2016
NMG/10/2020
HB/1231/2021
SD/9192/2022
JS/1278/2022
NMG/0326/2023

J

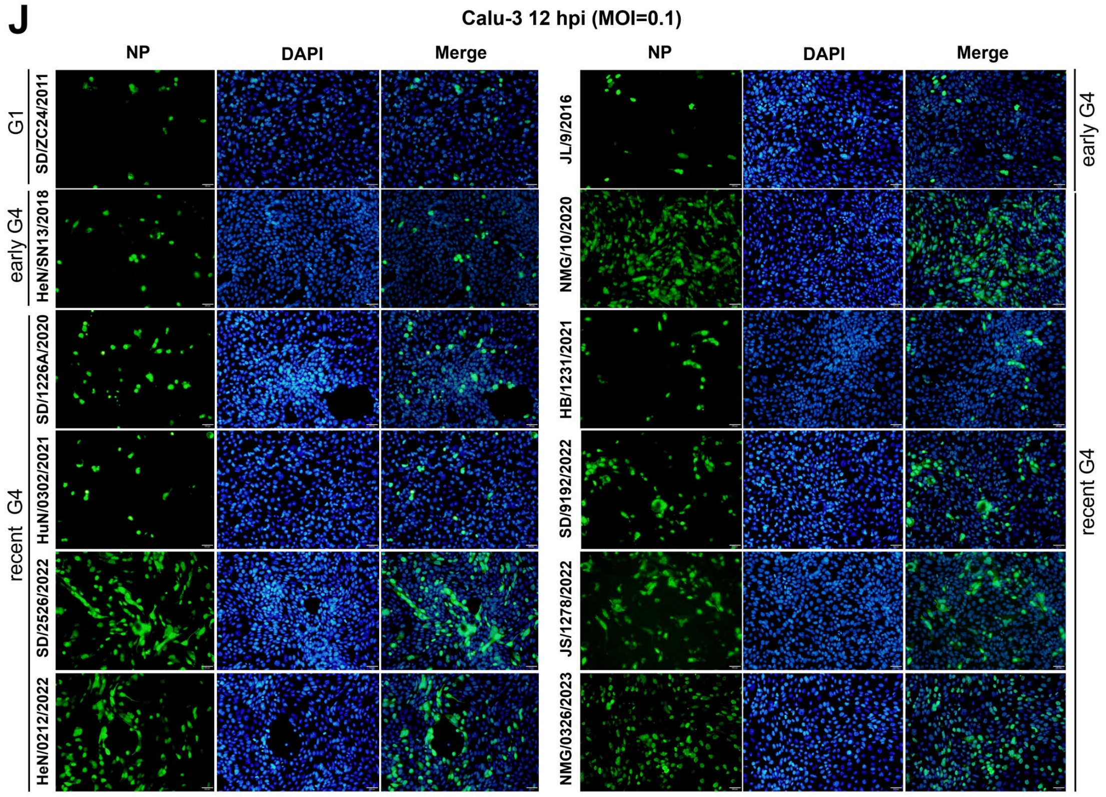
Calu-3 12 hpi (MOI=0.1)
NP
DAPI
Merge
G1
early G4
recent G4
SD/ZC24/2011
HeN/SN13/2018
SD/1226A/2020
HuN/0302/2021
SD/2526/2022
HeN/0212/2022
JL/9/2016
NMG/10/2020
HB/1231/2021
SD/9192/2022
JS/1278/2022
NMG/0326/2023

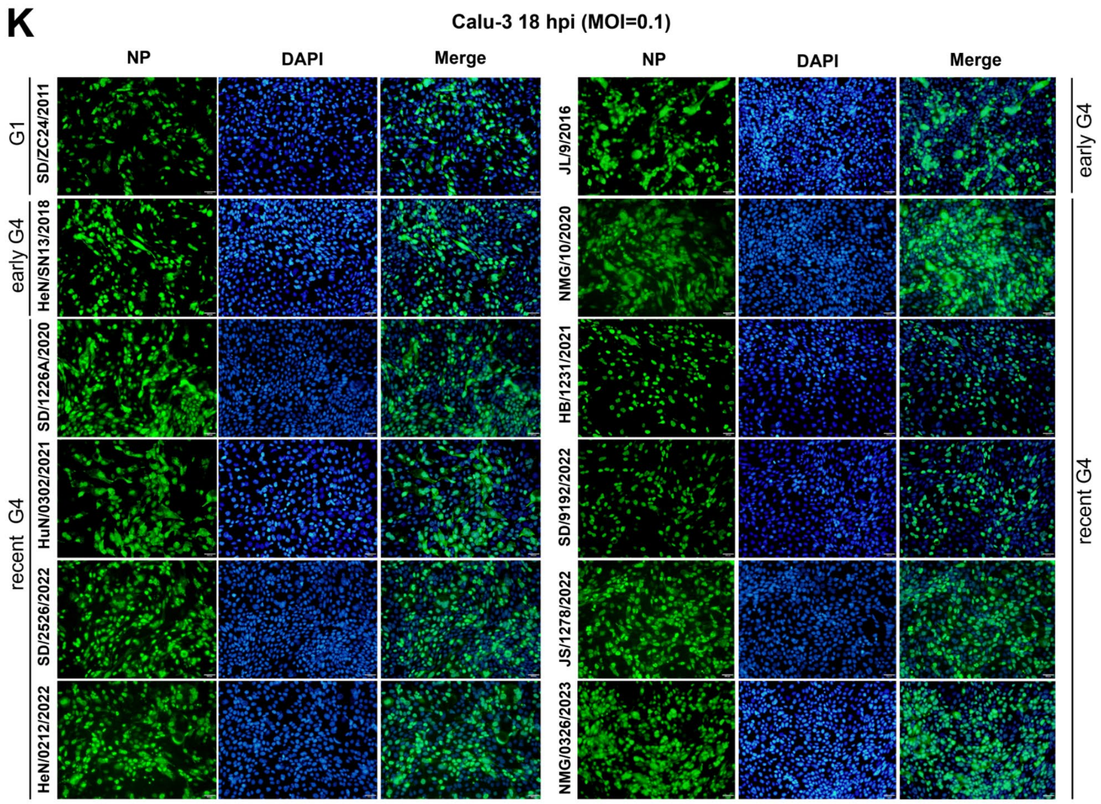
K
Calu-3 18 hpi (MOI=0.1)
NP
DAPI
Merge
NP
DAPI
Merge
G1
SD/ZC24/2011
early G4
HeN/SN13/2018
recent G4
SD/1226A/2020
HuN/0302/2021
SD/2526/2022
HeN/0212/2022
JL/9/2016
early G4
NMG/10/2020
HB/1231/2021
SD/9192/2022
JS/1278/2022
NMG/0326/2023
recent G4

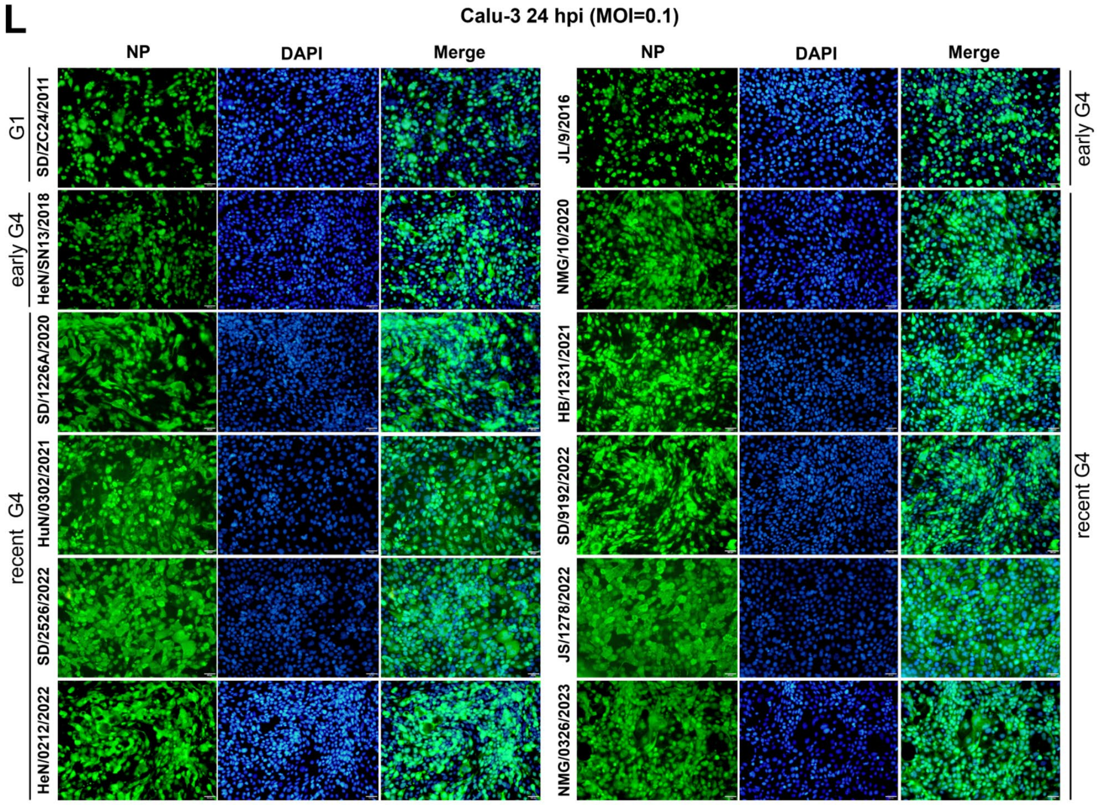
L
Calu-3 24 hpi (MOI=0.1)
NP
DAPI
Merge
NP
DAPI
Merge
G1
SD/ZC24/2011
early G4
HeN/SN13/2018
recent G4
SD/1226A/2020
HuN/0302/2021
SD/2526/2022
HeN/0212/2022
JL/9/2016
early G4
NMG/10/2020
HB/1231/2021
SD/9192/2022
JS/1278/2022
NMG/0326/2023
recent G4

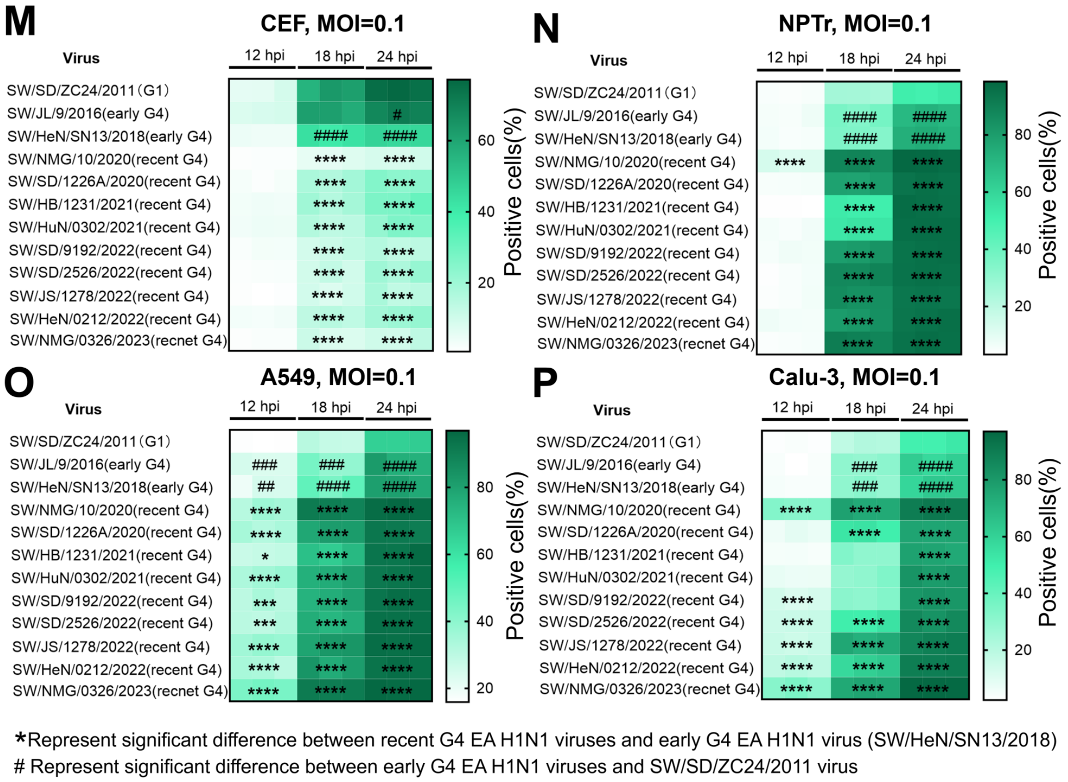

Figure.S3. Infection of G4 EA H1N1 viruses in avian or mammalian cells.

CEFs(A-C), NPTr cells (D-F), A549 cells (G-I), and Calu-3 cells (J-L) were infected with the indicated viruses at an MOI of 0.1 for 12h, 18h and 24h. Influenza virus NP was detected at indicated time points with fluorescein isothiocyanate (FITC)-conjugated goat anti-mouse antibody. Nuclei were detected with DAPI. Scale bars, 100 µm.

(M-P) Percentages of influenza NP-positive cells per DAPI-positive cells were calculated. Values are expressed as mean ± SD of three randomly selected fields. Data at each time point was analyzed by one-way ANOVA. ns, not significant; the * represent significant difference (*$p < 0.05$, **$p < 0.01$, ***$p < 0.001$, ****$p < 0.0001$) between recent G4 EA H1N1 virus-infected cells and SW/HeN/SN13/2018 (the virus that infect fewer CEFs and more mammalian cells in two early G4 EA H1N1 strains) virus-infected cells. The # represent significant difference ($^{\#}p < 0.05$, $^{\#\#}p < 0.01$, $^{\#\#\#}p < 0.001$, $^{\#\#\#\#}p < 0.0001$) between early G4 EA H1N1 viruses and G1 EA H1N1 virus (SW/SD/ZC24/2011).
