# Supplementary material for: Continuous evolution of Eurasian avian-like H1N1 swine influenza viruses with pdm/09-derived internal genes enhances pathogenicity in mice
Source: J Virol. 2025 Sep 8;99(10):e00430-25. doi: 10.1128/jvi.00430-25 (PMC12548388; doi:10.1128/jvi.00430-25)
Supplement: Additional supplemental material — Figures S4 and S5; Tables S1 to S4. [file jvi.00430-25-s0003.pdf]

Supplementary Materials

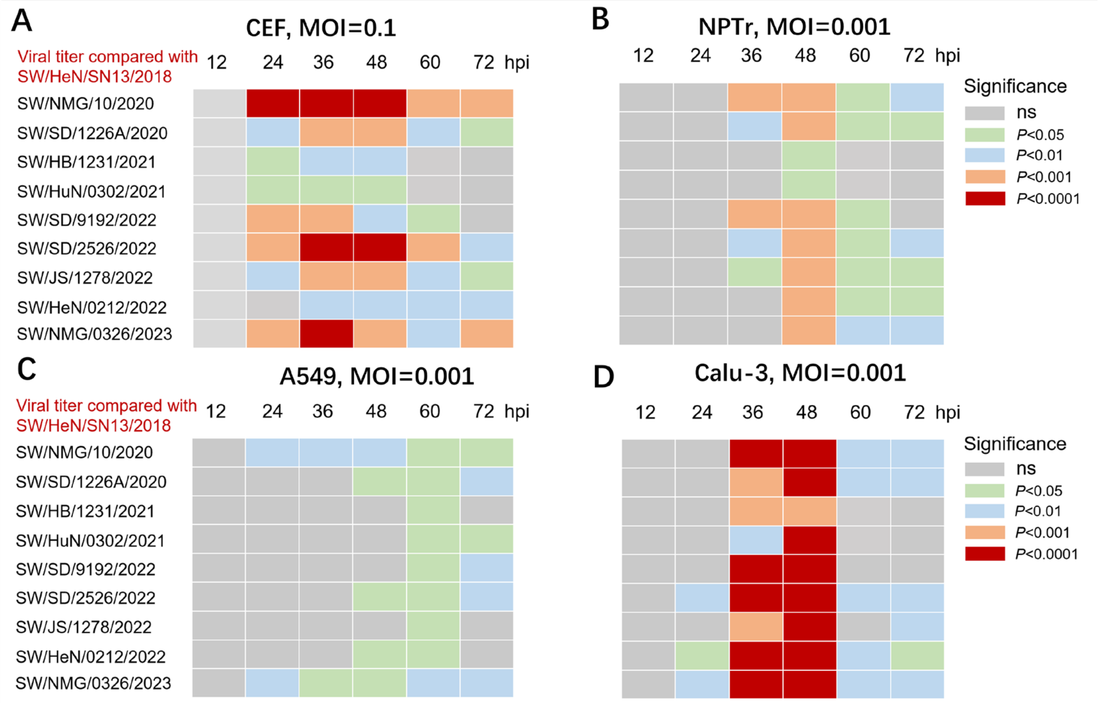

Figure.S4. Comparison of viral titers between the early and recent G4 EA H1N1 strains in avian or mammalian cells. Growth kinetics of EA H1N1 viruses were ascertained in CEFs, NPTr cells, A549 cells, and Calu-3 cells infected with an MOI of 0.01. At indicated time points, cell culture supernatants were collected and virus titers were determined by $TCID_{50}$ assays on MDCK cells. Values are expressed as mean ± SD of three randomly selected fields. Data were analyzed by two-way ANOVA. The significant difference ($p < 0.05$, $p < 0.01$, $p < 0.001$, $p < 0.0001$, ns, not significant) between recent G4 EA H1N1 virus-infected cells and SW/HeN/SN13/2018 (this early G4 EA H1N1 has lower titers in CEFs and higher titers in mammalian cells) virus-infected cells at each time point were marked with different color in the heatmap.

A

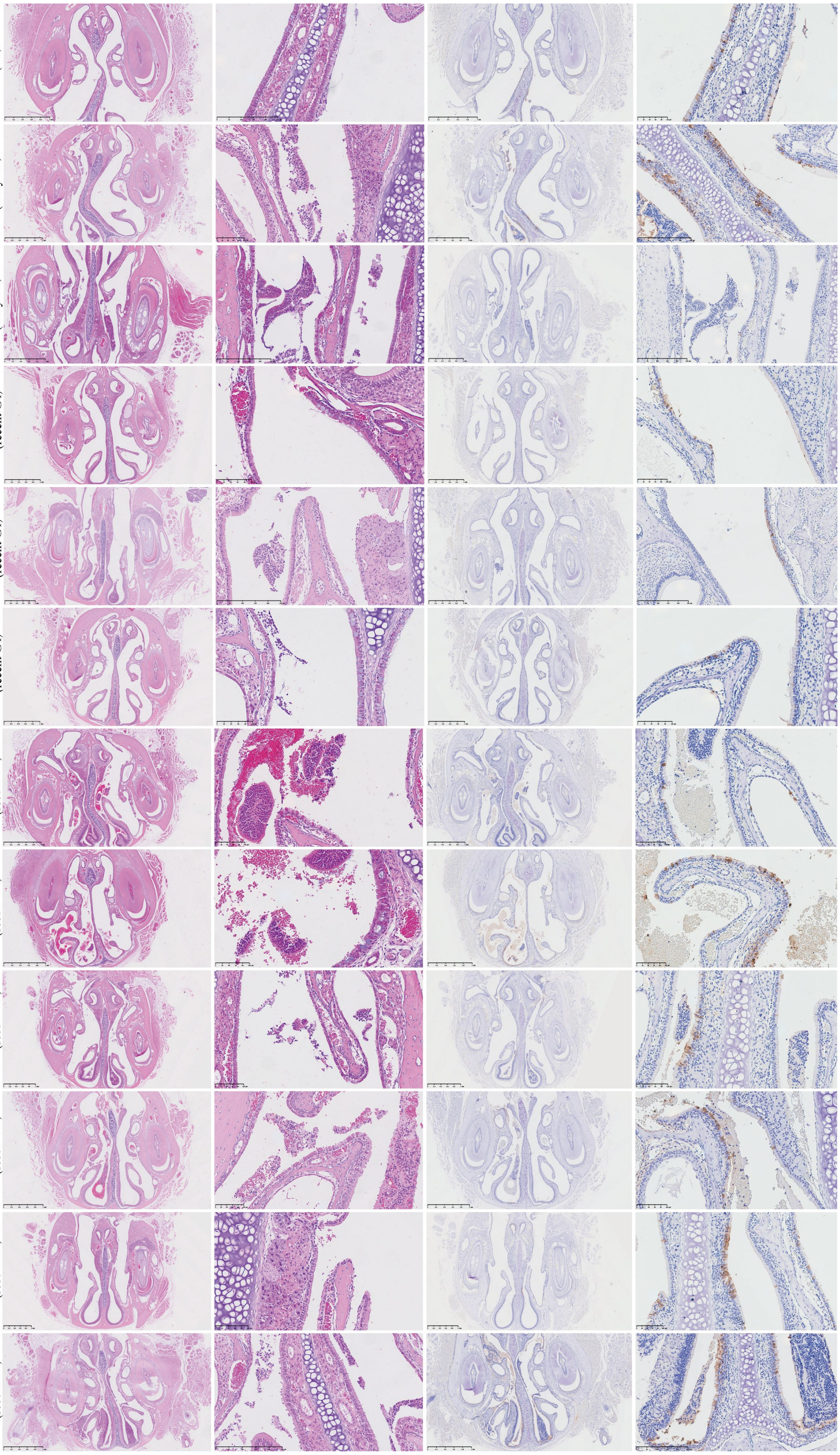
SW/SD/ZC24/2011 (G1)
SW/JL/9/2016 (early G4)
SW/HeN/SN13/2018 (early G4)
SW/NMG/10/2020 (recent G4)
SW/SD/1226A/2020 (recent G4)
SW/HB/1231/2021 (recent G4)
SW/HuN/0302/2021 (recent G4)
SW/SD/9192/2022 (recent G4)
SW/SD/2526/2022 (recent G4)
SW/JS/1278/2022 (recent G4)
SW/HeN/0212/2022 (recent G4)
SW/HeN/0326/2023 (recent G4)

B

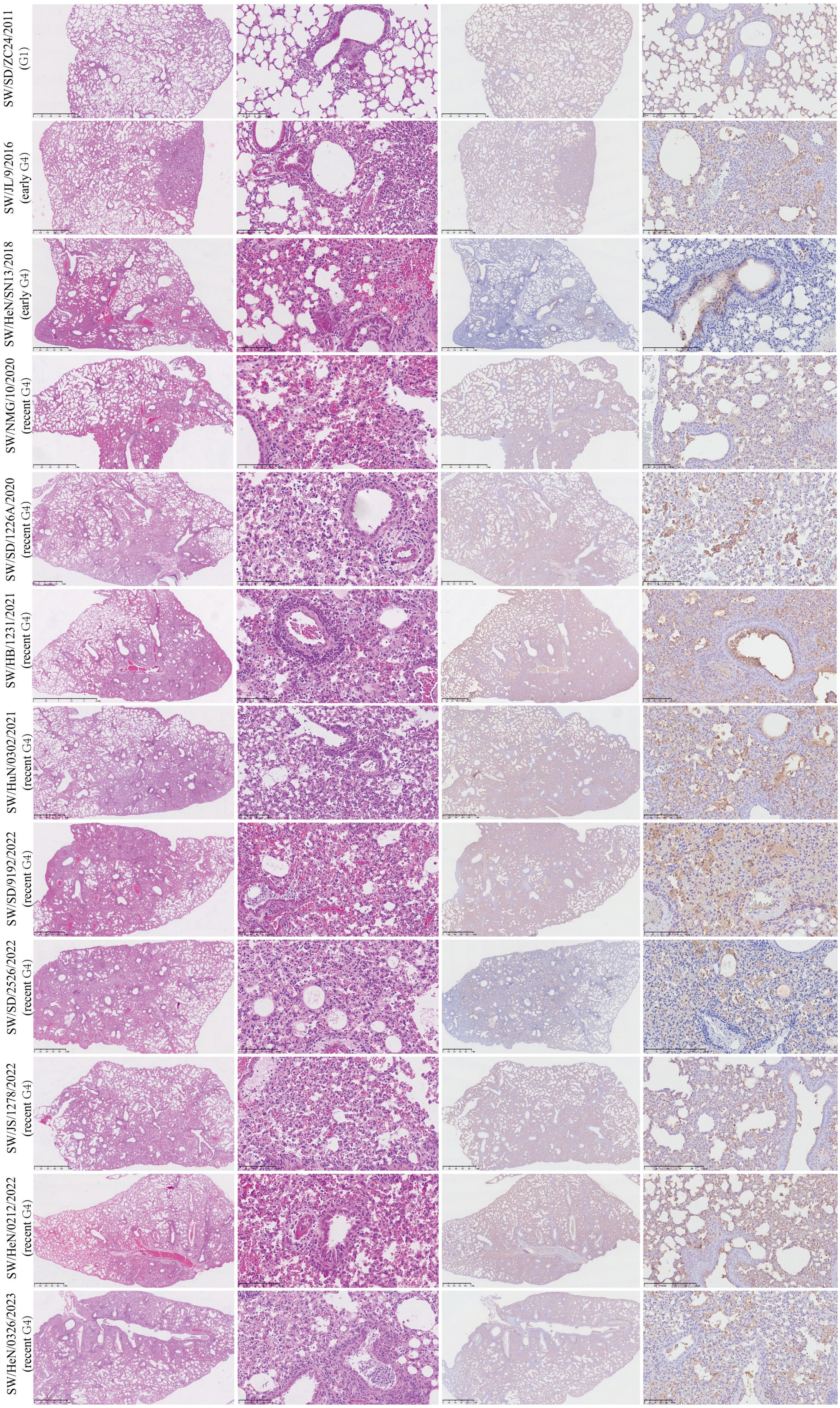
SW/SD/ZC24/2011
(G1)
SW/JL/9/2016
(early G4)
SW/HeN/SN13/2018
(early G4)
SW/NMG/10/2020
(recent G4)
SW/SD/1226A/2020
(recent G4)
SW/HB/1231/2021
(recent G4)
SW/HuN/0302/2021
(recent G4)
SW/SD/9192/2022
(recent G4)
SW/SD/2526/2022
(recent G4)
SW/JS/1278/2022
(recent G4)
SW/HeN/0212/2022
(recent G4)
SW/HeN/0326/2023
(recent G4)

Figure.S5. Histopathology of mice infected with G4 EA H1N1 viruses. Representative histopathological findings in the turbinate (A) and lung (B) of mice infected with the indicated viruses at 4 dpi. The turbinate and lung sections were stained with H&E (left) and by IHC against influenza viral NP antigen (right). Scale bars, 100 μm.

Table S1. Samples collected for influenza virus isolation from 2019 to 2023

Year	Number of samples	Number of virus-positive samples	Isolation rate (%)	Virus subtype			
				EA H1N1	H1(EA)N2	H1(pdm09)N2	H3N2
2019	899	9	10.01	9	0	0	0
2020	1840	11	0.59	5	2	1	3
2021	1286	4	0.31	3	0	0	1
2022	2946	14	0.47	14	0	0	0
2023	708	4	0.56	4	0	0	0
Total	7679	42	0.54	35	2	1	4

Table S2. Detailed information of SIVs isolated in this study

Strain name	Collection date	Collection place	Subtype	chicken embryonated egg	MDCK cell
A/Swine/Henan/0315/2019	2019-03-15	Henan	EA H1N1	+	+
A/Swine/Henan/0318/2019	2019-03-18	Henan	EA H1N1	+	+
A/Swine/Jiangsu/1010/2019	2019-10-10	Jiangsu	EA H1N1	+	+
A/Swine/Henan/15/2019	2019-11-15	Henan	EA H1N1	+	+
A/Swine/Henan/16/2019	2019-11-15	Henan	EA H1N1	+	+
A/Swine/Hebei/1125/2019	2019-11-25	Hebei	EA H1N1	+	+
A/Swine/Jiangsu/1213/2019	2019-12-13	Jiangsu	EA H1N1	+	+
A/Swine/Jiangsu/1215/2019	2019-12-15	Jiangsu	EA H1N1	+	+
A/Swine/Hebei/20/2019	2019-12-20	Hebei	EA H1N1	+	+
A/Swine/Shandong/105/2020	2020-01-05	Shandong	EA H1N2	+	+
A/Swine/Shandong/1226A/2020	2020-03-20	Shandong	EA H1N1	+	+
A/Swine/Liaoning/321/2020	2020-03-21	Liaoning	EA H1N2	+	+
A/Swine/Neimenggu/10/2020	2020-10-20	Neimenggu	EA H1N1	-	+
A/Swine/Hebei/11/2020	2020-10-25	Hebei	EA H1N1	-	+
A/Swine/Shandong/12/2020	2020-12-10	Shandong	pdm09 H1N2	-	+
A/Swine/Shandong/13/2020	2020-12-16	Shandong	EA H1N1	-	+
A/Swine/Shandong/S21/2020	2020-11-21	Shandong	Human like H3N2	-	+
A/Swine/Shandong/1224C/2020	2020-12-20	Shandong	Human like H3N2	-	+
A/Swine/Shandong/1224D/2020	2020-12-20	Shandong	Human like H3N2	-	+
A/Swine/Hebei/23/2020	2020-12-23	Hebei	EA H1N1	+	+
A/Swine/Shandong/ZYZ/2021	2021-01-01	Shandong	Human like H3N2	+	+
A/Swine/Hunan/0302/2021	2021-03-02	Hunan	EA H1N1	-	+
A/Swine/Hebei/Y10/2021	2021-10-10	Hebei	EA H1N1	+	+
A/Swine/Hebei/1231/2021	2021-12-31	Hebei	EA H1N1	-	+
A/Swine/Henan/0212/2022	2022-02-12	Henan	EA H1N1	+	+
A/Swine/Shandong/0328/2022	2022-03-28	Shandong	EA H1N1	-	+
A/Swine/Shandong/1018/2022	2022-10-18	Shandong	EA H1N1	+	+
A/Swine/Jiangsu/5354/2022	2022-10-20	Jiangsu	EA H1N1	+	+
A/Swine/Jiangsu/6162/2022	2022-10-21	Jiangsu	EA H1N1	-	+
A/Swine/Shandong/3940/2022	2022-10-22	Shandong	EA H1N1	+	+
A/Swine/Shandong/3738/2022	2022-10-22	Shandong	EA H1N1	+	+
A/Swine/Shandong/1067/2022	2022-10-24	Shandong	EA H1N1	+	+
A/Swine/Shandong/1378/2022	2022-11-10	Shandong	EA H1N1	-	+
A/Swine/Shandong/3536/2022	2022-11-15	Shandong	EA H1N1	-	+
A/Swine/Shandong/2526/2022	2022-11-15	Shandong	EA H1N1	-	+
A/Swine/Shandong/9596/2022	2022-11-15	Shandong	EA H1N1	-	+
A/Swine/Shandong/9192/2022	2022-11-15	Shandong	EA H1N1	-	+
A/Swine/Jiangsu/1278/2022	2022-11-25	Jiangsu	EA H1N1	-	+
A/Swine/Shandong/108/2023	2023-01-08	Shandong	EA H1N1	-	+
A/Swine/Jiangsu/114/2023	2023-01-14	Jiangsu	EA H1N1	-	+
A/Swine/Shandong/115/2023	2023-03-10	Shandong	EA H1N1	+	+
A/Swine/Neimenggu/0326/2023	2023-03-26	Neimenggu	EA H1N1	-	+

Table S3. Antigenic classification of EA H1N1 SIVs by HI assays

Test viruses (Genotype)	Antisera			
	SW/SD/ZC24/2011 (G1 EA H1N1)	SW/JS/J006/2018 (G4 EA H1N1)	SW/HeN/SN13/2018 (G4 EA H1N1)	BJ/0212/2019 (pdm09 H1N1)
BJ/0212/2019(pdm09 H1N1)	40	<20	80	**1280†**
A				
SW/HeN/08/2011 (G1)	**2560†**	160	80	40
SW/SD/ZC22/2011 (G1)	**2560†**	80	80	40
B				
SW/JL/9/2016 (G4)	80	640	640	<20
SW/JS/J006/2018 (G4)	80	**1280†**	640	<20
SW/HeN/SN13/2018 (G4)	80	640	**1280†**	<20
SW/SD/1226A/2020 (G4)	80	1280	640	<20
SW/NMG/10/2020 (G4)	40	1280	1280	<20
SW/HB/1231/2021 (G4)	80	640	640	<20
SW/HuN/0302/2021 (G4)	40	640	640	20
SW/SD/9192/2022 (G4)	80	1280	1280	<20
SW/SD/2526/2022 (G4)	80	640	640	<20
SW/SD/3536/2022 (G4)	80	640	640	<20
SW/SD/3940/2022 (G4)	80	1280	640	<20
SW/SD/0328/2022 (G4)	40	640	640	<20
SW/HeN/0212/2022 (G4)	80	640	640	<20
SW/JS/1278/2022 (G4)	40	640	640	20
SW/SD/108/2023 (G4)	80	640	640	<20
SW/NMG/0326/2023 (G4)	80	1280	1280	<20

*Virus names are abbreviated as isolation location, strain name and collection year. Data represent HI titers. High HI titers (≥ 640) are shaded blue, moderate HI titers (160, 320) are gray and low HI titers (<80) are not shaded.

†Homologous titer.

Table.S4 Mean evolutionary rate(substitutions/site/year) of H1N1 viruses (human pdm/09 H1N1 viruses and swine G4 EA H1N1 viruses), as estimated by BEAST analysis.

**Segment**	**Host**	**Mean rate**	**Fold change (Swine /human)**	**Lower 95% CI***	**Upper 95% CI***
PB2	Human	1.9402×10^{-3}	1.82	1.9369×10^{-3}	1.9434×10^{-3}
	Swine	3.5436×10^{-3}		3.5383×10^{-3}	3.5490×10^{-3}
PB1	Human	1.7441×10^{-3}	1.96	1.7409×10^{-3}	1.7472×10^{-3}
	Swine	3.4176×10^{-3}		3.4105×10^{-3}	3.4247×10^{-3}
PA	Human	2.2184×10^{-3}	1.41	2.2149×10^{-3}	2.2220×10^{-3}
	Swine	3.1352×10^{-3}		3.1300×10^{-3}	3.1405×10^{-3}
NP	Human	2.0279×10^{-3}	1.62	2.0241×10^{-3}	2.0316×10^{-3}
	Swine	3.2958×10^{-3}		3.2912×10^{-3}	3.3003×10^{-3}
M	Human	1.8748×10^{-3}	1.51	1.8707×10^{-3}	1.8791×10^{-3}
	Swine	2.8417×10^{-3}		2.8362×10^{-3}	2.8472×10^{-3}

*The 95% CI represents the upper and lower bounds of the highest posterior density. The CI is the shortest interval that contains 95% of the sampled values.
