# Supplementary material for: Continuous evolution of Eurasian avian-like H1N1 swine influenza viruses with pdm/09-derived internal genes enhances pathogenicity in mice
Source: J Virol. 2025 Sep 8;99(10):e00430-25. doi: 10.1128/jvi.00430-25 (PMC12548388; doi:10.1128/jvi.00430-25)
Supplement: Tables S5 and S6 — Parameters for Bayesian molecular clock analysis and parameters used for scoring of histopathology lesions in lung. [file jvi.00430-25-s0004.pdf]

Supplementary Materials

Table.S5 Parameters for Bayesian molecular clock analysis

Segment	ESS value						number of trees used
	joint	prior	likelihood	treeModel.rootHeight	age(root)	treeLength	
PB2	4684	4511	17250	15574	15574	767	72008
PB1	1317	1407	4365	5026	5026	265	27003
PA	866	914	1766	2111	2111	204	18002
NP	1896	1957	1509	2952	2952	255	9001
M	948	963	822	2182	2182	268	9001

Table.S6 Parameters used for scoring of histopathology lesions in lung.

**Score**	**0 (None)**	**1 (Minimal)**	**2 (Mild)**	**3 (Moderate)**	**4 (Severe)**
Epithelial necrosis, attenuation or disruption	None	Rare foci affecting 1-2 airways	Affecting more than 2 airways and up to one third of airways	Affecting more than one third and up to two thirds of airways	Affecting more than two thirds of airways
Level of inflammation	No inflammation	Sparsely scattered granulocytic inflammatory cells affecting occasional airways	More than a few scattered neutrophils or eosinophils (e.g. intraluminal aggregation(s)) affecting up to one third of airways	Inflammation as score 2 affecting more than one third, and up to two thirds, of airways	Inflammation as score 2 affecting more than two thirds of airways
Peribronchiolar and perivascular lymphocytic cuffing	No discernible peribronchiolar/ perivascular cuffing	Occasional incomplete, or loosely formed, cuffs or lymphocytic aggregations	Numerous cuffs, predominantly incomplete and loosely-formed with lesser well-formed complete cuffs	Numerous cuffs, approximately half or more well-formed, and may have a few broad, dense cuffs	Numerous cuffs, predominantly well-formed with numerous broad, dense cuffs
Alveolar cellular exudate/edema and interlobular edema	None	Occasional alveoli affected–eosinophilic fluid	Confluent alveoli and/or interlobular septal involvement affecting up to one third of lung	Confluent alveoli and/or interlobular septal involvement affecting more than one third and up to two thirds of lung	Confluent alveoli and/or interlobular septal involvement affecting more than two thirds of lung
Alveolar septal inflammatory cells and cellularity	Septae typically 1-2, or occasionally 3, nucleated cells wide and absence of inflammatory cells	As score 1 but with scattered inflammatory cells within alveolar walls- often granulocytes most likely within blood vessels	Focal or multifocal alveolar septal inflammation with regions of mild thickening of septae and increased mononuclear cells (affected septae 3-4 nuclei wide)	Focal or multifocal septal inflammation with regions of moderate thickening of septae and increased mononuclear cells (affected septae 5 or more nuclei wide).	Coalescing to diffuse alveolar septal inflammation (septae 3 or more cells wide)
